# FIRM image analysis: A machine learning workflow for quantifying extracellular matrix components from electron microscopy images

**DOI:** 10.1371/journal.pone.0312196

**Published:** 2025-02-06

**Authors:** Nicholas T. Gigliotti, Justin Lee, Emily H. Mang, Giancarlo R. Zambrano, Mitra L. Taheri

**Affiliations:** 1 Department of Materials Science and Engineering, Whiting School of Engineering, Johns Hopkins University, Baltimore, Maryland, United States of America; 2 Department of Mechanical Engineering, Whiting School of Engineering, Johns Hopkins University, Baltimore, Maryland, United States of America; 3 Department of Chemical and Biomolecular Engineering, Whiting School of Engineering, Johns Hopkins University, Baltimore, Maryland, United States of America; KIST: Korea Institute of Science and Technology, GERMANY

## Abstract

The extracellular matrix (ECM) is a complex network of biomolecules that plays an integral role in the structure, processes, and signaling mechanisms of cells and tissues. Identifying and quantifying changes in these matrix components provides insight into the mechanisms behind specific tissue remodeling processes; however, quantifying these changes is challenging due to difficult imaging conditions, complexity of the ECM, and the subtlety of these changes. Current imaging techniques allow us to visualize these critical remodeling events and developments in image analysis have employed a combination of analysis software and machine learning techniques to improve the efficiency and accuracy with which features are measured. Although image analysis has seen much improvement in recent years, there has been no technique developed to address ambiguity in feature edges in electron microscopy images. Presented here is a new machine learning-based workflow for the analysis of microscopy images named FIRM (Feature Identification from Raw Microscopy) that uses a random forest classifier to identify ECM features of interest and generate binary segmentation masks for quantification with ImageJ-FIJI. FIRM performed with an F1 score of 0.794 and greater than 80% accuracy for number and size of features detected. FIRM had similar deviation from the ground truth in the number of identified fibrils, fibril size, and size distributions when compared to human analyses. The results suggest that FIRM performs as well as manual analysis and requires a fraction of the time. This analysis technique is more efficient, eliminates user bias, and can be easily optimized to identify a variety of features, making it useful for any discipline requiring image analysis.

## Introduction

The extracellular matrix (ECM), which is a matrix rich in proteins and other biological molecules that provides structure and support to cells and tissues, has long been studied to understand the pathways and mechanisms by which cells and tissues signal each other, remodel, and respond to changes in their environment [1, 2]. Advancements in biological microscopy have made it possible to image the ECM at various length scales, providing information about the structure and composition and how it changes over time [3]. The ability to accurately identify and quantify features of interest in images of the ECM presents difficulties due to the complexity of the ECM and often poor imaging conditions. Due to inherent water content in biological samples, lack of heavy atoms, and short sample life, significant sample processing is required to achieve images in an electron microscope. While fixation techniques and the use of stains have made biological electron microscopy possible, it is difficult to manipulate delicate soft tissue samples without damaging the tissue. Stains, which are used to improve the contrast of features of interest, often leave staining artifacts, which impact contrast and the ability to threshold images for analysis. Additional difficulties arise with maintaining sample orientation through sectioning to achieve ideal imaging planes. In many previous studies, researchers used programs like ImageJ-FIJI to aid in image analysis [4–10]. In some studies, analysis software was used as a measurement tool for quantifying feature geometries [7–10]. Others used available software packages to segment the image based on a user defined threshold, separating the images into features and background [11–18]. Although ImageJ-FIJI greatly reduces time spent on manual analysis and has a variety of tools for different analysis methods, there are still sources of error due to inconsistent image processing and the need for human input to determine appropriate feature boundaries. Especially in cases of poor imaging conditions or sample preparation, high levels of noise can result in ambiguous feature edges, leaving their interpretation subjective to the researcher. This introduces bias and makes tracking feature evolution through various time points unreliable.

**Fig 1:**
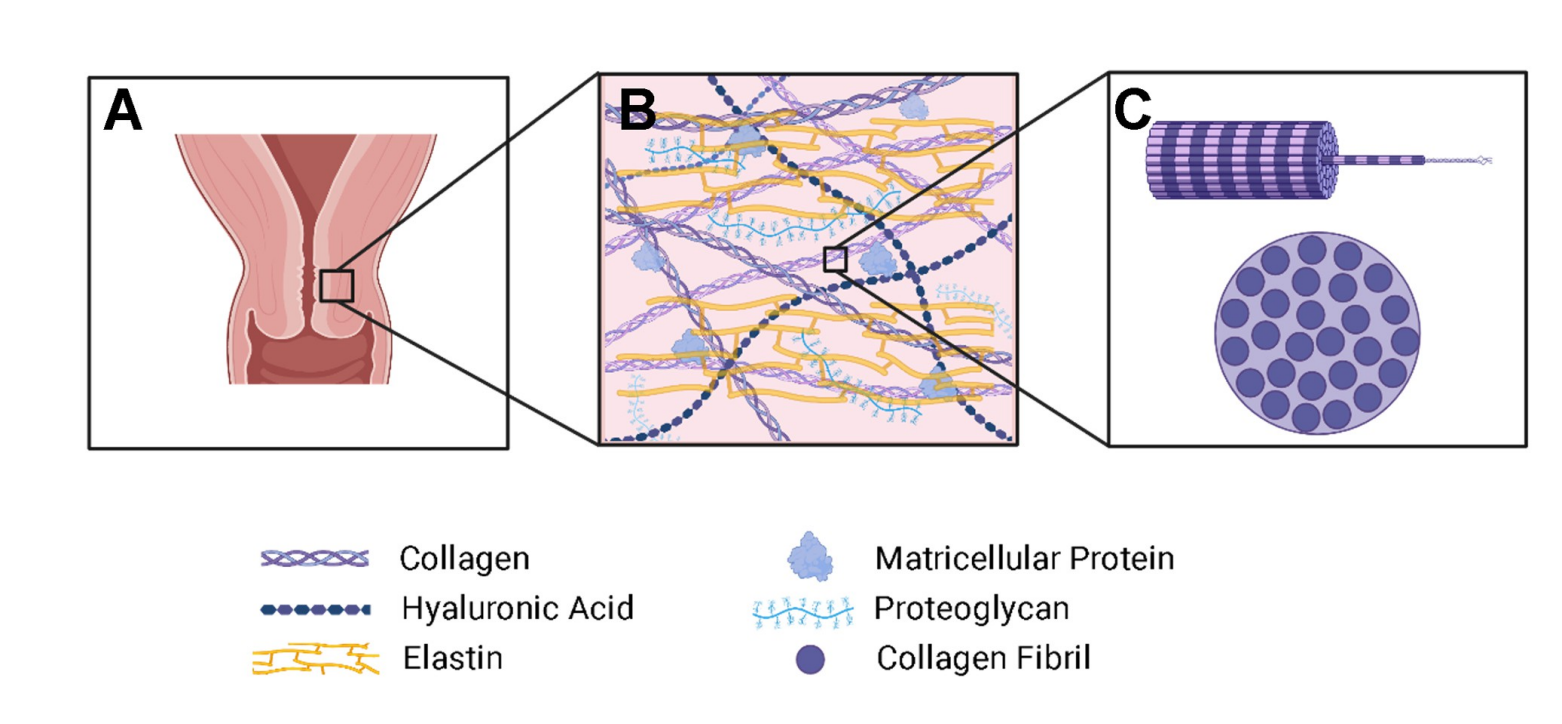
Schematic of the cervix ECM. The cervix (A) undergoes changes in the ECM (B) that contribute to its changing mechanical properties through pregnancy. Collagen is the main structural component of the ECM and is a primary feature of interest in understanding the remodeling process. (C) shows the cross-section of a collagen fiber, which is comprised of bundles of fibrils that undergo changes in size and density throughout pregnancy. Created with Biorender.com.

**Fig 2:**
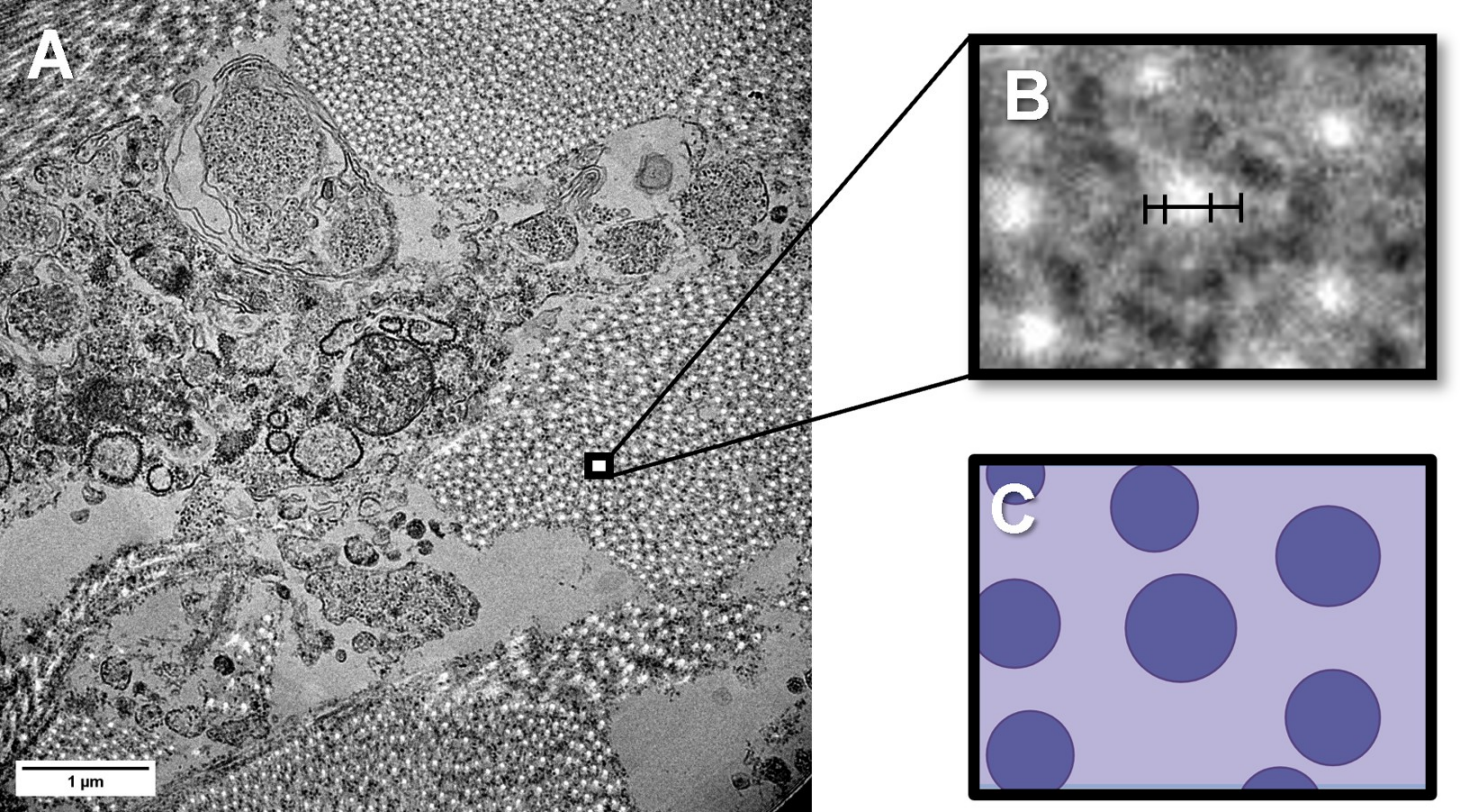
TEM of collagen in the murine cervix. (A) TEM micrograph of pregnant mouse cervix tissue showing collagen fibril cross-sections. (B) The magnified section demonstrates 2 ways to define ambiguous fibril edges based on either the bright centers or darker edges. (C) An idealized schematic of the magnified inset showing collagen fibril cross sections. Figure created with Biorender.com.

An apt example of difficult imaging conditions impacting ECM studies can be found in TEM micrographs of mouse cervix sections. The cervix (Fig 1), which undergoes significant remodeling during pregnancy, has been identified as a critical component that contributes to preterm birth [19, 20]. Since the primary structural component of the cervix is collagen, tracking collagen fibril evolution through pregnancy is necessary for understanding remodeling mechanisms. Due to limited samples and difficult imaging conditions, the fibril cross sections shown in Fig 2 appear to have two boundaries, neither of which are well-defined and are highly dependent on image contrast, making it nearly impossible to reliably determine the degree of swelling or shrinking from one image to another. To define features accurately and consistently in the ECM and reliably study their evolution in EM images, a new method for analysis is required. Using a machine learning model to identify the fibrils without bias or human error will allow for a more reliable view of the ECM changes in the cervix.

Machine learning (ML) has become a topic of targeted development in recent years to aid in image analysis, making research more efficient and accurate. Generalized toolkits that utilize ML provide solutions for a variety of applications and can be trained in real time as images are collected on a microscope [21–24]. Although programs such as this are powerful and pervasive, they are not specifically optimized for niche applications like fibril detection in TEM images and often require training input from the individual as they are imaging, which still introduces user bias in how the features are defined.

More specific tools have also been developed for analyzing biological images acquired through a variety of imaging modes, including but not limited to histological staining [25], electron microscopy (EM) [26–28], MRI [29], and polarized light microscopy [30, 31]. The two machine learning algorithms that are most commonly used are random forest classifiers (RFs) and convolutional neural networks (CNNs). In many of these examples, algorithms are used to classify a variety of ECM and other biological features based on manually annotated training data sets. Some of these ML tools rely on histological staining to classify collagen and other ECM components, using color values or hue as defining variables in the segmentation processes. This aids in defining features but is not applicable to all imaging modes. For example, EM images, which provide high resolution images on the nanometer scale, are grayscale, making them more challenging to classify accurately. Other ML tools have been developed that are designed for grayscale images, making them applicable for electron microscopy; however, these models are designed for specific applications that target classification [25–31], tracking objects through 3D image stacks [26–28], or identifying density [25, 30, 31] and do not emphasize distinct edge definition for quantitative size measurements.

To the best of our knowledge, no prior ML based workflow has been presented that addresses the segmentation and analysis of collagen fibrils in EM images. Here we present FIRM, a machine learning classification approach utilizing a random forest classifier to address this problem. RF classifiers are well established in the machine learning community and are commonly used for image classification problems. They also provide a distinct advantage over CNNs because they require minimal training data sets to accurately classify features. For applications that require tissue samples from animal trials, it is difficult to gather large training data sets due to limited sample collection.

FIRM utilizes manually annotated images as a training set for the RF classifier. Features are extracted from the training data set with a variety of image filters, and the classification model is fit to the training set. Once the model is trained, new images can be analyzed to identify features of interest. Output masks are generated that segment the features of interest, which can be quantitatively analyzed through ImageJ-FIJI. For collagen fibril identification and resulting measurements, the model shows sufficient agreement with the established ground truth, performing equal to individual human reviewers. Size and number determination by FIRM consistently presented with an error of less than 20%. Precision and recall accuracy were also determined which resulted in an F_1_ score across all test images of 0.794. This method eliminates human subjectivity in defining feature edges and allows for rapid iteration through large data sets with limited training images, limiting total image analysis time. FIRM can be tailored to identify a variety of features with different image filters, making it a powerful imaging tool not only for ECM quantification, but for any application requiring contrast-dependent object detection analysis in grayscale electron microscopy images.

## Methods

### Mouse cervix image generation

Pregnant and non-pregnant CD-1 mice were acquired from Charles River Laboratories (Wilmington, Massachusetts, United States) and sacrificed via CO_2_ inhalation. Cervix tissue samples were isolated from the mice via dissection and were fixed in 4% formaldehyde – 1% glutaraldehyde fixative. Following a wash with S-Collidine buffer, the samples were post-fixed with 1% osmium tetroxide for 1 hour, dehydrated in graded concentrations of acetone, and embedded in epoxy resin. Thick sections were cut at 2 microns using Leica 6 ultramicrotome (Wetzlar, Germany) and stained with 0.1% toluidine blue. Ultrathin 90 nm sections were placed on copper grids, stained with 3% uranyl acetate and 0.4% lead citrate, and examined with a JEOL JEM-1230 transmission electron microscope (Akishima, Tokyo, Japan) operated at 80 kV. Digital images were acquired using an Advanced Microscopy Techniques camera. Experiments were performed with approval from the University of Pennsylvania’s Institutional Animal Care and Use Committee (IACUC #805513) and adhered to the National Institute of Health Guidelines on Laboratory Animals.

### Random forest detection model

**Fig 3:**
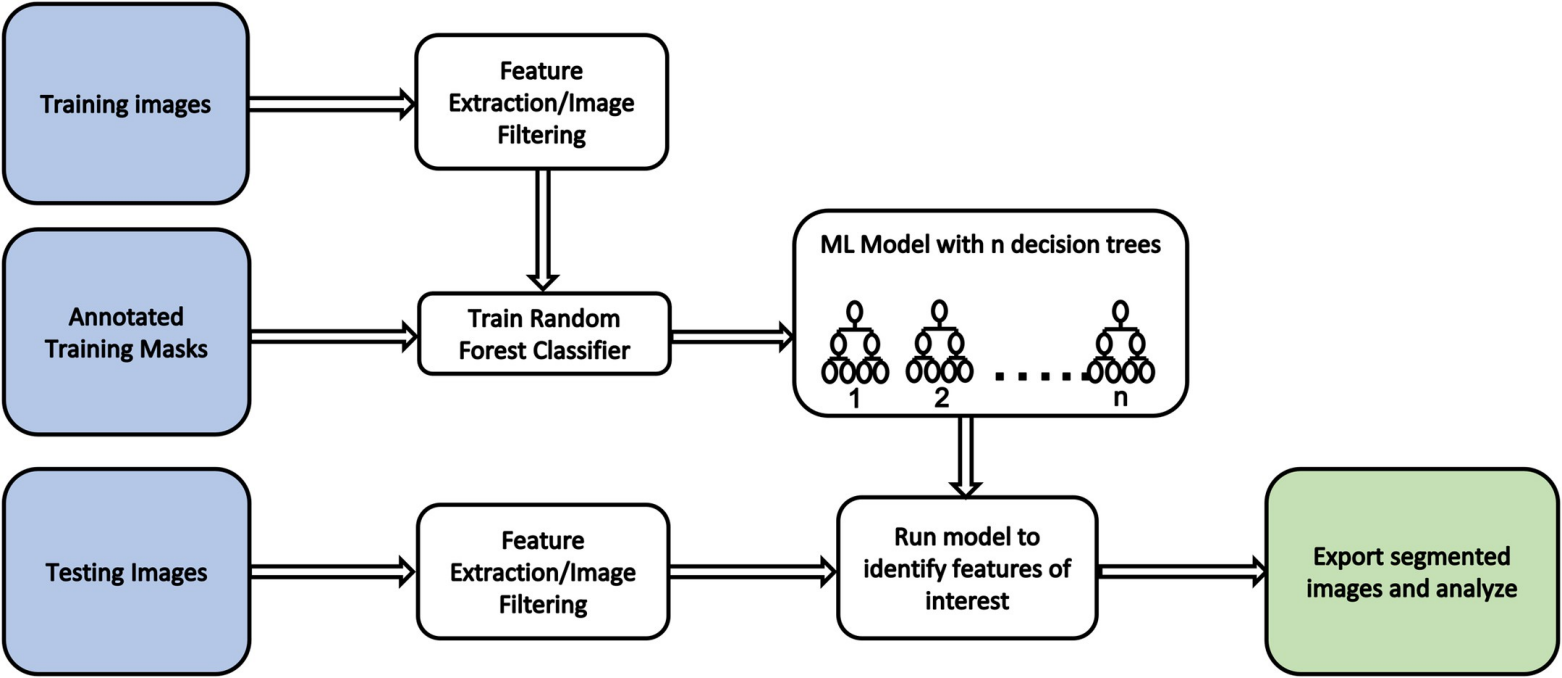
FIRM workflow. Flow chart showing the FIRM workflow used to train the random forest classifier and detect features from test images.

Image segmentation for FIRM is performed using a random forest classifier in Python and was built with dependencies on the following components: scikit-learn [32], scikit-image [33], pandas [34], numPy [35], OpenCV [36], PIL [37], matplotlib [38], joblib [39], and sciPy [40]. To train the classifier, 5 TEM images of mouse cervix sections were chosen based on image quality and fibril orientation. To create segmentation masks for training, the 5 training images were manually annotated using APEER annotation software [41] by 3 reviewers who were instructed to identify fibril cross-sections and background regions, resulting in 3 separate annotations for each image. Only one annotated mask was chosen at random for each image to account for variability in how fibril edges were defined between reviewers. Masks were exported to ImageJ-FIJI to define pixel values for each class. Training image pixel values were read using Python and feature extraction was performed by generating new images with filters optimized for collagen fibril detection based on edge contrast effects. Filters included Canny Edge, Roberts Edge, Sobel, Scharr, Prewitt, Gaussian, and Median filters. The minimum and maximum pixel values used for the Canny filter were 100 and 200 respectively. For Gaussian filters, sigma values of 1 and 3 were used. For the Median filter, a sigma value of 3 was used. The random forest classifier model was imported from the scikit-learn package and fitted to the training image sets and corresponding annotated masks with n = 8 decision trees. The data extracted from the training images was randomly split so that 80% of the dataset is used to train the model while 20% is reserved for testing model accuracy. This model was then loaded into a new script to perform feature detection. To test the model’s ability to detect fibrils, 11 new TEM images with a variety of collagen packing scenarios were selected for testing. The same filters were applied to the test images for feature extraction and the images were fed into the model for classification. Image post-processing was performed using a Gaussian blur with a kernel size of 7 to remove Gaussian noise and a threshold blur with minimum and maximum pixel values set to 100 and 255. Features were labelled and exported as a segmented mask. The FIRM workflow is outlined in Fig 3. All images used are available in the S2 Dataset.

### Quantitative measurements of fibril size

ImageJ-FIJI was used to quantify the size of the fibrils from the segmented mask obtained from both the random forest classifier and the manually annotated masks. Mean fibril diameter, size distribution, and number of fibrils were determined for both manually annotated masks and model-generated masks using the particle analysis tool. Ellipses were fit to each fibril and the minor axis was taken as the diameter to account for non-normal fibril orientations. Size filtering was applied to eliminate detection of background features. Features with areas greater than 5000 nm^2^ or less than 10 nm^2^ were discarded. Size distributions were determined by counting the number of fibrils in designated bins based on fibril diameter. Bin size was 10nm with 14 bins ranging from 10nm to 150nm.

### Evaluation of FIRM identification and segmentation of fibrils

FIRM’s ability to properly identify and segment fibrils was evaluated by comparing model-generated masks to designated “ground truth” masks of the same TEM micrographs. To establish a “ground truth” for a given micrograph, 3 reviewers were asked to annotate the fibrils in the image and generate binary segmentation masks. The three resulting masks were merged and only areas where at least two of three reviewers indicated a fibril were kept, removing any outlier annotations. Precision and recall metrics were evaluated to determine how accurately the model identifies fibrils in the correct location. To achieve this, model-generated masks were stacked with the corresponding ground truth mask and each fibril was classified as a true positive (TP) or false positive (FP). This determination was made by setting a threshold for the intersection over union (IoU) value, or the area of the feature overlap divided by the area of the union of the features, for each fibril. IoU values equal to or exceeding 0.33 were considered true positives. Values below 0.33 resulted in false positives, indicating that the model incorrectly identified the location of the fibril. This threshold was chosen instead of the conventional IoU = 0.5 because in some cases, fibril edge ambiguity may result in differences in area of up to 200% depending on edge definition. Consequently, identified fibrils may be wrongfully assigned as false positives even if the model-generated and ground truth fibrils are completely overlapped due to differences in area. Precision was calculated with Eq 1 and indicates what fraction of the model-identified fibrils were identified correctly.


$$P=\frac{TP}{TP+FP}$$ (Eq 1)

Recall indicates how many of the fibrils the model accurately identified compared to the total number in the ground truth (N_GT_) and is calculated using Eq 2.


$$R=\frac{TP}{N_{GT}}$$ (Eq 2)

Using these two metrics, the F_1_ score for the model was calculated using Eq 3 and provides a single value representing the harmonic mean of the precision and recall.


$$F_{1}=2\frac{P*R}{P+R}$$ (Eq 3)

### Evaluation of FIRM performance vs other methods

The number of fibrils identified, average fibril size, and the fibril size distribution were identified as key parameters to evaluate the performance of the model compared to other methods. Ilastik, a RF based toolkit for image segmentation and analysis, was used to analyze the dataset to compare the performance of FIRM against Ilastik, another open-source RF classifier [21]. Human analyses were also compared to FIRM to assess performance compared to traditional manual analysis. The results for each method were plotted in a parity plot against “ground truth” values, and the coefficient of determination statistic, R^2^, was reported as a measure of agreement between measured data and the ground truth. To account for variability in human performance, 3 individual reviewers were asked to manually annotate the fibrils, generate binary segmentation masks, and analyze the fibril count, size, and size distribution via the particle analysis tool in ImageJ-FIJI. Individual R^2^ values as well as an average R^2^ value from all 3 reviewers were reported. Additionally, precision, recall, and F1 scores were determined for Ilastik to further compare the accuracy of the two ML-based methods.

### Evaluation of model training on FIRM performance

Further investigation into the impact of training data on segmentation accuracy was also performed. Although FIRM has been designed to utilize only a few training images (n = 5) to limit computational costs and annotation requirements, training sets of 7 and 10 images were also considered. Fibril count, size, and distribution were measured and compared to assess whether the model improved with more training images. Training sets above 10 images were computationally expensive and were not considered. In addition to training set size, the number of annotations included in each training image were examined since RF classifiers may be adequately trained with sparse annotations. Segmentation masks were generated using FIRM with 25%, 50%, 75%, and 100% fibril annotations in the training set. Segmented masks were overlain with the original TEM images and qualitatively compared.

## Results and discussion

**Fig 4:**
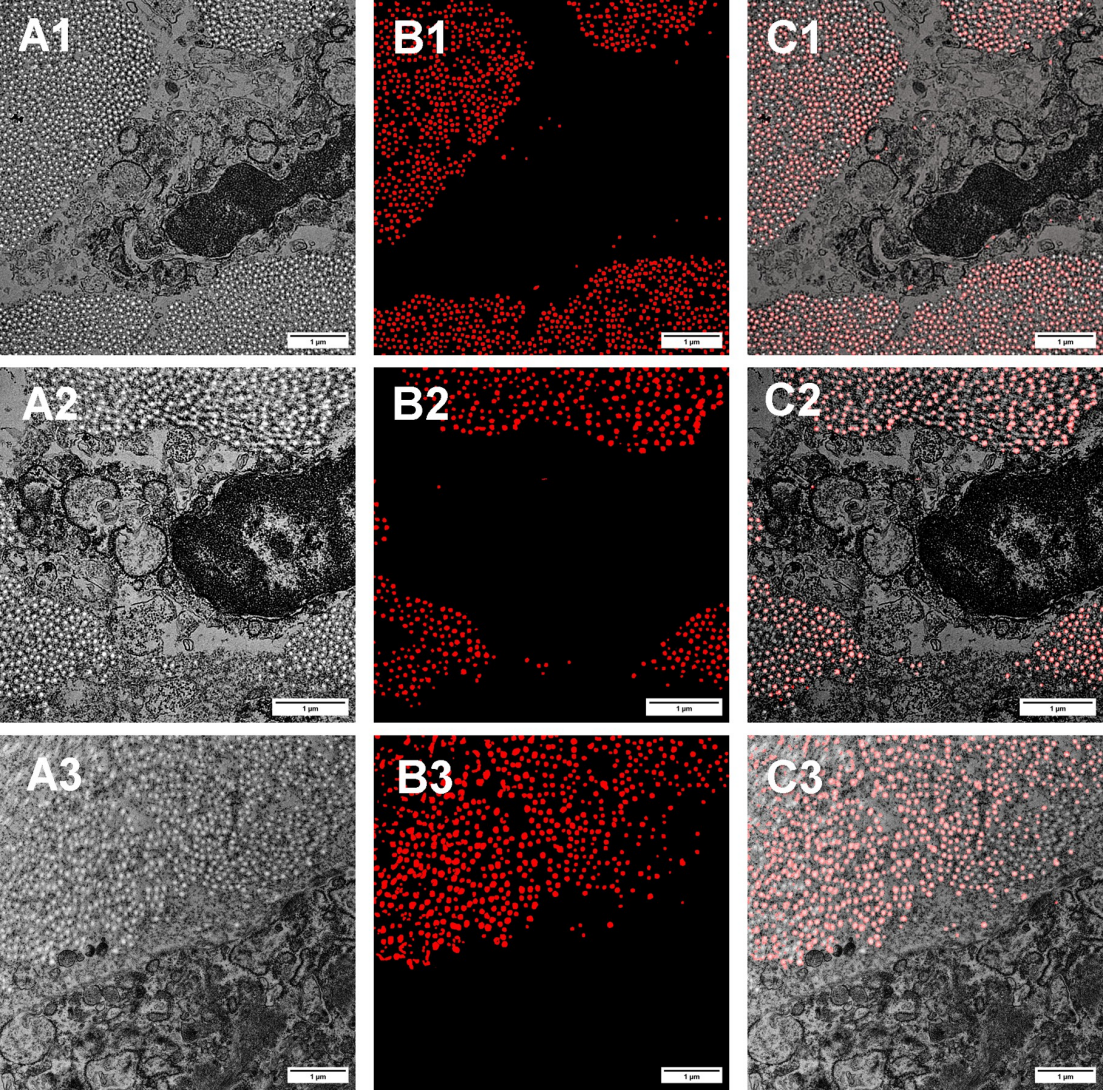
TEM micrographs with FIRM segmentation. Column A) Original TEM images of mouse cervix tissue. Column B) FIRM-generated segmentation masks of identified collagen fibrils. Column C) FIRM-detected fibrils (red) overlain with the corresponding TEM micrographs to demonstrate accuracy of feature identification.

Fig 4 shows a subset of the original TEM micrographs with corresponding model-generated masks and an overlay showing a visual representation of the agreement between the model and original images. The overlay demonstrates that a majority of the identified fibrils appear in the same locations as the fibrils from the original image, qualitatively showing FIRM’s ability to identify features of interest.

**Fig 5:**
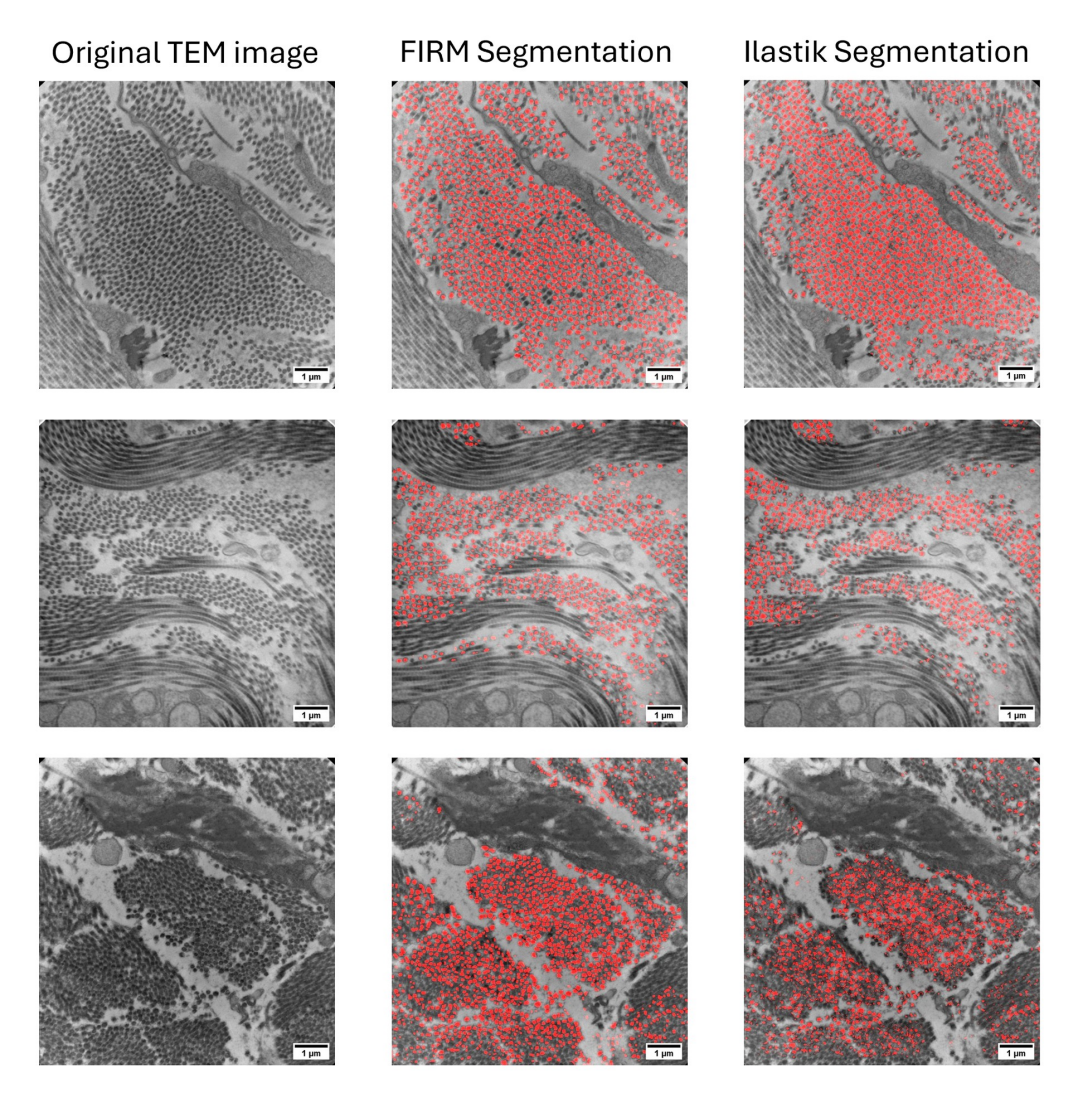
Visual comparison of FIRM and Ilastik segmentation of collagen fibrils in TEM images. The left column contains original TEM images, the middle column contains overlays of the FIRM segmentation masks, and the right column contains overlays of the Ilastik segmentation masks. ML-segmented collagen fibrils appear in red.

Precision and recall evaluations are shown in Table 1. Precision scores varied between 0.739 and 0.986 and recall scores varied between 0.578 and 0.939 depending on the image selected. Across all images, the model has a precision of 0.821 and recall of 0.769, resulting in an F_1_ score of 0.794 which demonstrates similar performance to other machine learning classification models developed for biological imaging applications. Chen et al. presented four models for segmentation-based feature extraction for 3D cryo-electron imaging of proteins. Precision ranged from 0.61 to 0.79, recall ranged from 0.59–0.81, and F_1_ scores ranged from 0.66 to 0.80 [26]. Another example, Touma et al., utilized Google AutoML to classify cataract phases from surgical videos. They reported an F_1_ score of 0.79 with a precision of 0.81 and a recall of 0.77 [42]. Sheneman et al. explored the use of machine learning methods to segment lipid droplets imaged via quantitative phase imaging. The random forest classifier they employed performed with an F_1_ score of 0.86, precision of 0.89, and recall of 0.82 [43]. Comparing the values from these studies with FIRM, FIRM performs comparably with other machine learning segmentation-based tools. Table 2 contains precision and recall evaluations for Ilastik, another RF classifier tool available for segmentation and analysis [21]. When performed on a subset of the same data, FIRM consistently outperformed Ilastik, which achieved F1 scores ranging from 0.384–0.78 with an overall F_1_ score of 0.547. Although both Ilastik and FIRM rely on RF classification, FIRM uses a greater variety of edge detection filters, whereas Ilastik only incorporates Gaussian based edge filters. Fig 5 shows a visual comparison of FIRM and Ilastik segmentation. Although FIRM’s performance metrics achieve reasonable success compared to both manual and other ML-based methods, the performance metrics are inconsistent from image to image. Comparing the values for precision and recall with a visual assessment of the image quality and collagen packing density suggests that the accuracy of the model is dependent on both the quality of the test image and how densely packed the collagen fibrils are. Despite this limitation, average performance metrics all fall within the ranges reported in literature.

**Table 1:** Summary of FIRM performance: Precision, recall, and F_1_ score.

Image ID	GT # of Fibrils	FIRM # of Fibrils	# of TP	# of FP	Precision	Recall	F_1_ Score
1	1356	1204	946	258	0.786	0.698	0.739
2	475	422	416	6	0.986	0.876	0.928
3	618	588	490	98	0.833	0.793	0.812
4	898	962	711	251	0.739	0.792	0.765
5	1936	2160	1818	342	0.842	0.939	0.888
6	1363	1492	1215	277	0.814	0.891	0.851
7	1920	1835	1356	479	0.739	0.706	0.722
8	1544	1297	1130	167	0.871	0.732	0.795
9	1174	798	679	119	0.851	0.578	0.689
10	875	793	589	204	0.743	0.673	0.706
11	1701	1437	1311	126	0.912	0.771	0.836
**Combined**	**13860**	**12988**	**10661**	**2327**	**0.821**	**0.769**	**0.794**

**Table 2:** Summary of Ilastik performance: Precision, recall, and F1 score.

Image ID	GT # of Fibrils	FIRM # of Fibrils	# of TP	# of FP	Precision	Recall	F_1_ Score
4	898	739	497	242	0.673	0.553	0.607
5	1936	1910	1038	872	0.543	0.536	0.540
6	1363	1367	1065	302	0.779	0.781	0.780
7	1920	1546	686	860	0.444	0.357	0.396
8	1544	1077	503	574	0.467	0.326	0.384
9	1174	756	565	191	0.747	0.481	0.585
10	875	544	322	222	0.592	0.368	0.454
11	1701	1063	902	161	0.849	0.530	0.653
**Combined**	**11411**	**9002**	**5578**	**3424**	**0.620**	**0.489**	**0.547**

**Fig 6:**
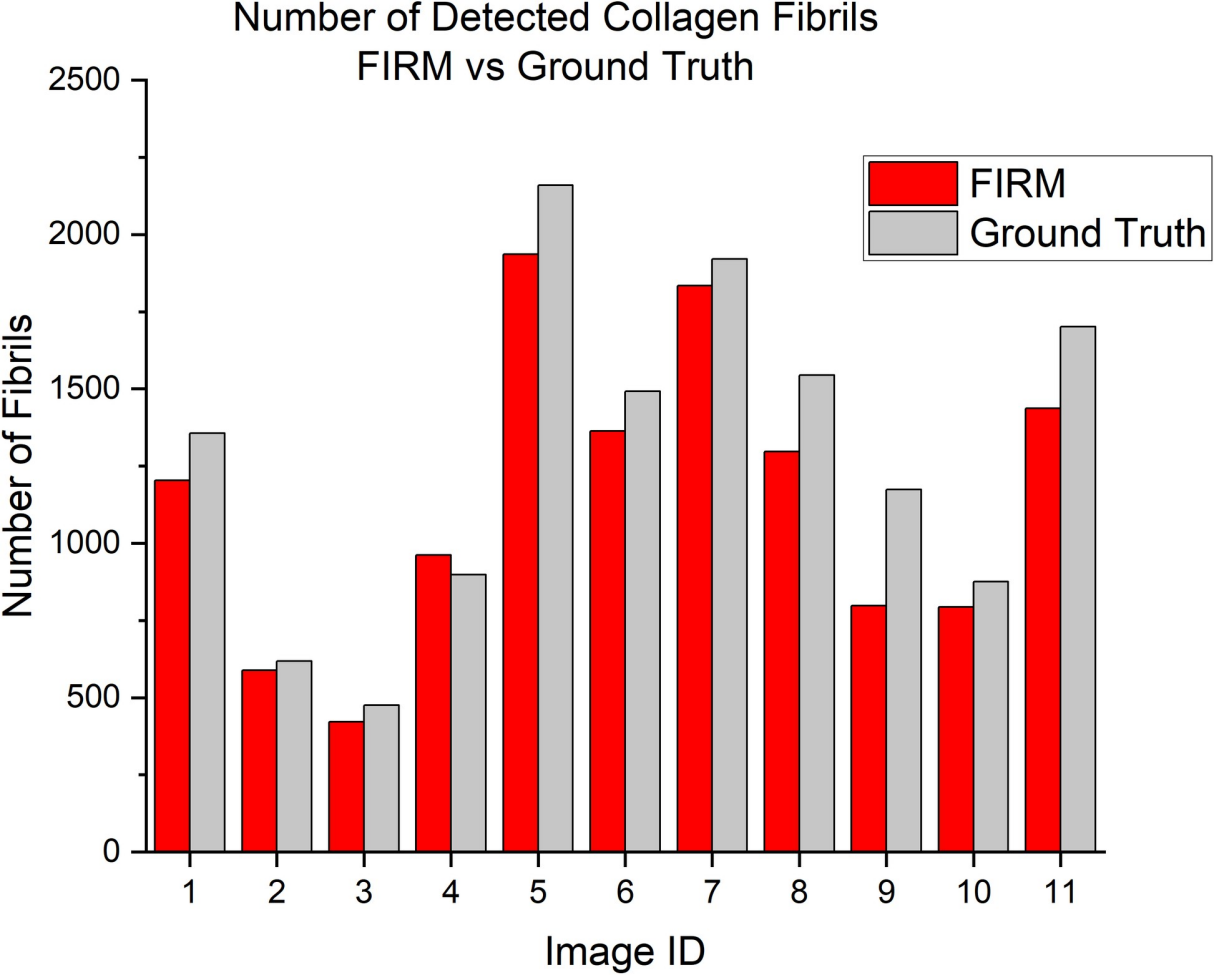
Number of collagen fibrils detected by FIRM compared to the ground truth for each test image.

**Fig 7:**
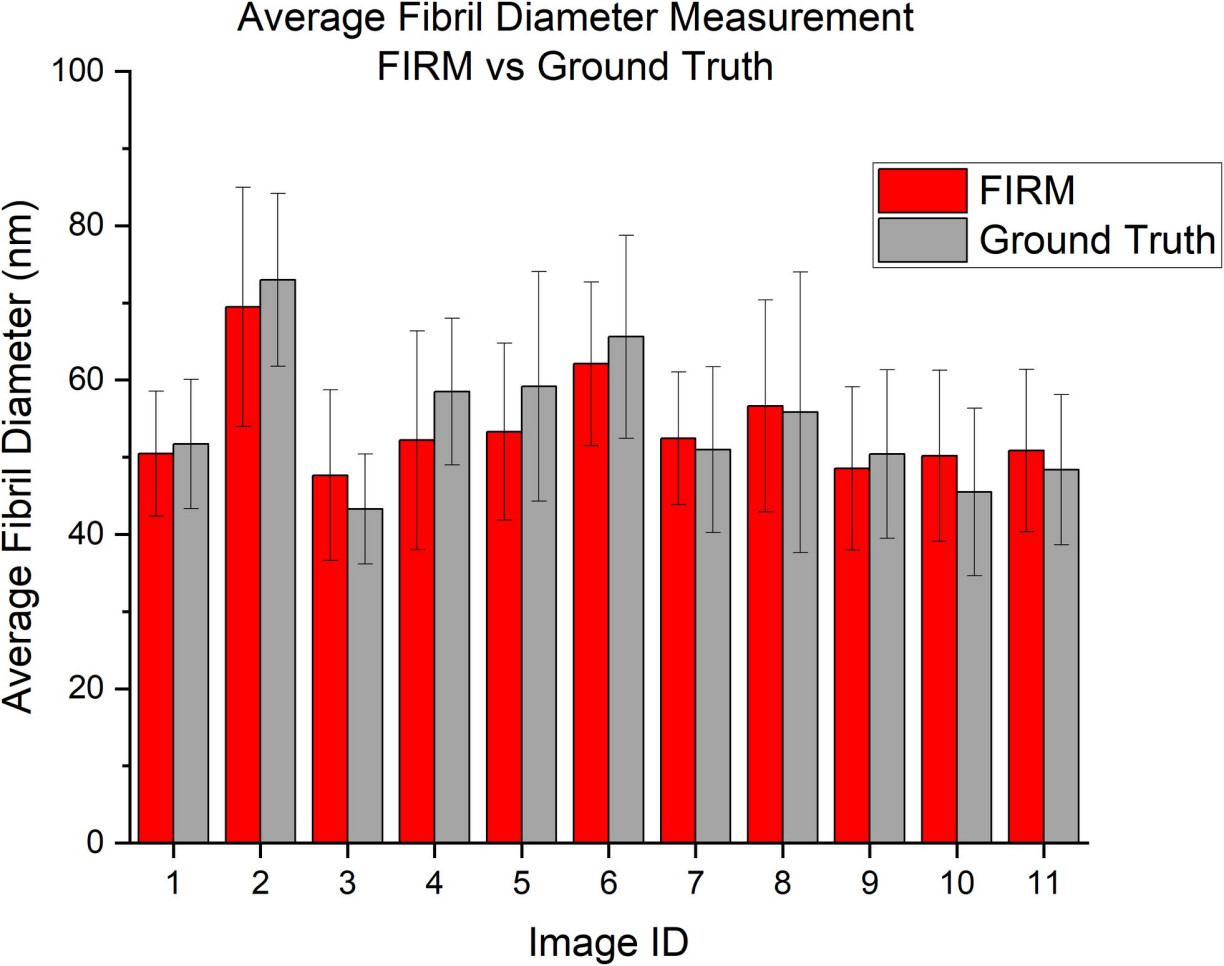
Average fibril diameter measured by FIRM compared to the ground truth for each image. Error bars represent standard deviation.

The number of identified fibrils and the average size of the fibrils are shown in Figs 6 and 7. Percent error calculations for number of detected fibrils and average fibril diameter (Table 3) show that the model consistently reports with less than 20% error compared to the ground truth, except for one outlier, Image 9.

**Table 3:** % Error calculations for FIRM compared to ground truth.

	Number of Detected Fibrils	Average Fibril Diameter
Image 1	11.21	2.46
Image 2	4.85	4.82
Image 3	11.16	10.07
Image 4	7.13	10.76
Image 5	10.37	9.93
Image 6	8.65	5.36
Image 7	4.43	2.86
Image 8	16.00	1.51
Image 9	32.03	3.74
Image 10	9.37	10.33
Image 11	15.52	5.14

**Fig 8:**
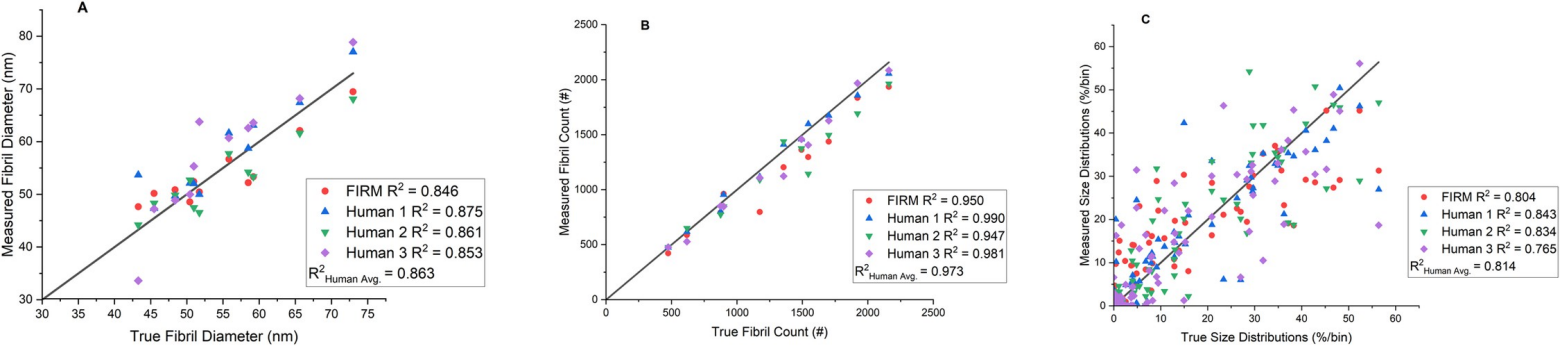
FIRM vs human analysis parity plots. Plots show ground truth and measured values of (A) average fibril size, (B) number of fibrils, and (C) fibril size distribution for FIRM and human analyses. Coefficient of determination (R^2^) is displayed for each dataset. The R^2^ = 1 line, which corresponds to the ground truth, is displayed for reference.

**Fig 9:**
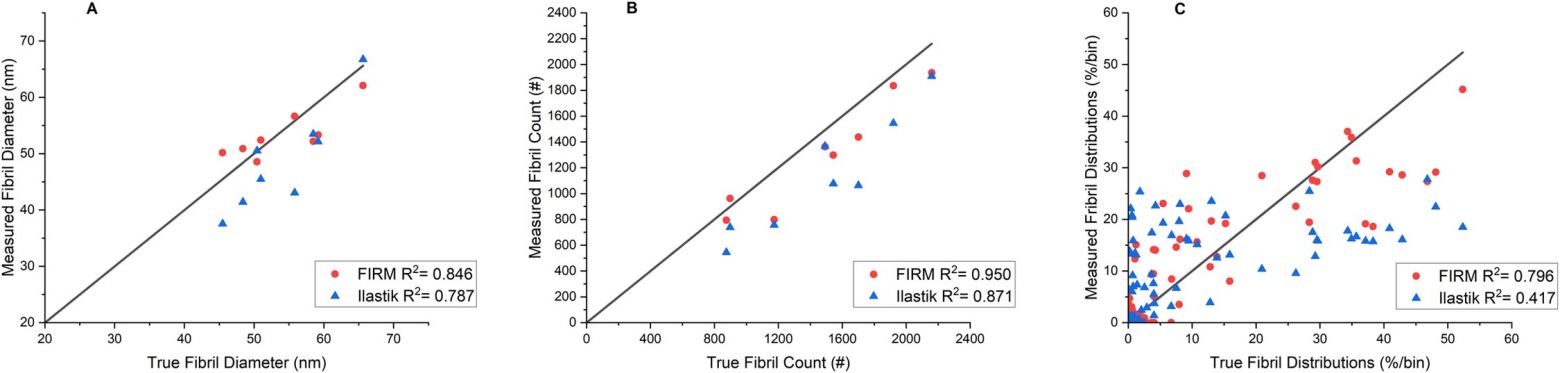
FIRM vs Ilastik parity plots. Plots show ground truth and measured values of (A) average fibril size, (B) number of fibrils, and (C) fibril size distribution for FIRM and Ilastik analyses. Coefficient of determination (R^2^) is displayed for each dataset. The R^2^ = 1 line, which corresponds to the ground truth, is displayed for reference.

Parity plots comparing FIRM to manual feature identification are shown in Fig 8 and comparing FIRM to Ilastik in Fig 9. R^2^ values indicate the deviation from the ground truth. For average fibril size across all 11 test images, R^2^ = 0.846 for FIRM while the average for human reviewers via manual methods was 0.863. When looking at how many fibrils were identified, R^2^ = 0.950 for FIRM and 0.973 for manual methods. To compare size distributions, each data point on the parity plot represents the percentage of fibrils that falls into a specified bin for a given image. All images were compiled into a single parity plot. R^2^ = 0.804 for FIRM and 0.814 for manual methods. For all three metrics, FIRM consistently performs similarly to manual methods. When comparing FIRM to Ilastik across 8 different images, FIRM achieved R^2^ values of 0.846, 0.950, and 0.796 for size, count, and distributions, respectively, whereas Ilastik achieved R^2^ values of 0.787, 0.871, and 0.417, demonstrating that FIRM outperforms Ilastik in each performance metric.

**Fig 10:**
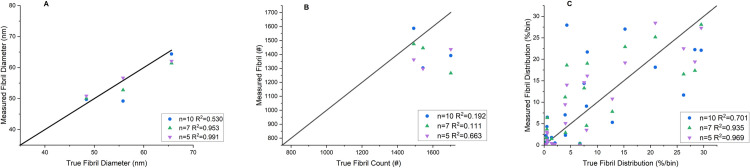
FIRM increased training set parity plots. Plots show ground truth and measured values for (A) average fibril size, (B) number of fibrils, and (C) fibril size distribution for FIRM with training sets of n = 5, 7, and 10 images. Coefficient of determination (R^2^) is displayed for each dataset. The R^2^ = 1 line, which corresponds to the ground truth, is displayed for reference.

To further investigate the use of the FIRM workflow for collagen analysis, the size of the training datasets and the number of annotations supplied were also examined. One of the advantages of the RF classifier employed in FIRM is that segmentation can be achieved with reasonable success using relatively small sets of training data. Fig 10 shows parity plots of fibril size, count, and distributions of FIRM operating with 5, 7, and 10 training images to assess the impact of larger training datasets on performance. It was found that the smaller training dataset outperformed the larger training sets in each performance metric, maintaining that operating FIRM with 5 training images balances performance and computational costs while avoiding overfitting.

**Fig 11:**
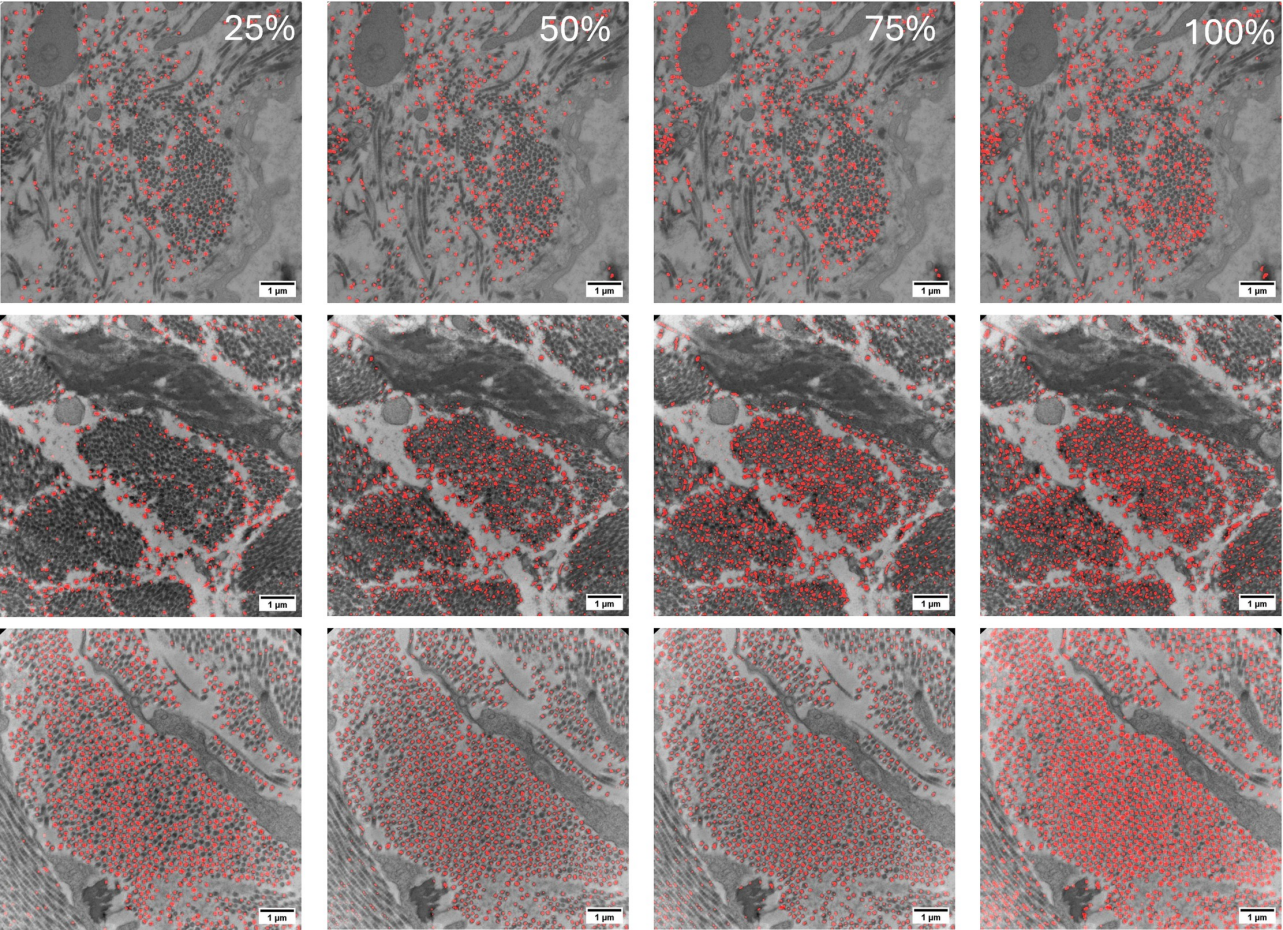
TEM micrographs with FIRM RF classifier overlays. From left to right, images show FIRM fibril detection using 25%, 50%, 75%, and 100% annotated images for model training.

Another advantage of RF classifiers is that in some instances they can be trained on sparse annotations rather than meticulously generated training masks and still achieve successful segmentation. Fig 11 shows a qualitative analysis of varying degrees of annotating for the training dataset. When only annotating 25% of the fibrils, the RF classifier still achieves moderate success at identifying fibrils on the image with clear, high contrast, moderately packed collagen fibrils (bottom). However, the sparse annotations lead to difficulties identifying fibrils in the images of densely packed fibrils (middle) or loosely packed collagen with non-fibril features scattered throughout (top). As the number of annotations increases, fibril detection continuously improves in all 3 images. While sparse training annotations can be sufficient and time-efficient for optimal images, in most cases it is recommended to fully annotate the training images for best results.

With a limited training data set of 5 images, FIRM was able to identify collagen fibril cross-sections in TEM images. The RF classifier identified the number and average size of the fibrils within a reasonable margin of error across all test images and had an F_1_ score that outperformed similar RF classifier-based tools on the same dataset and fell within the range for acceptable ML-based image segmentation presented in literature. Although the performance of FIRM is dependent on image quality and collagen packing, this workflow demonstrates that classification of ECM features in electron microscopy images can be achieved with accuracy and improved efficiency compared to manual methods despite a limited training data set. Furthermore, removing subjectivity from a reviewer’s interpretation of feature boundaries results in more consistent analyses from image to image, eliminating bias in studies where image analysis may not be completed consistently between two images.

Depending on the goals for a specific study, prioritization of specific performance metrics may be necessary. For tracking ECM component evolution through a remodeling process, a higher emphasis is placed on the size distribution, average fibril diameter, and F_1_ score. The number of fibrils detected is not as important as minimizing false positives. False positives can skew size related data, making it difficult to accurately compare fibril sizes throughout the remodeling process. False negatives are less detrimental to size measurements since they only serve to reduce sample size instead of adding incorrect data points. For the application presented in this work, it was concluded that FIRM performs sufficiently to replace manual analysis of EM images with the added benefits of reduced variation due to subjectivity in fibril edge definition and rapid iteration through large datasets. For other applications, it may be necessary to optimize the model for specific evaluation metrics so that the resulting classification is more relevant.

## Conclusion

The machine learning approach proposed in this study is a promising method for image quantification that can be used to identify and extract a variety of features for any application. FIRM-generated segmentation masks showed agreement in collagen fibril number, size, and distribution with the ground truth. Further analysis of the precision, recall, and F_1_ score showed that the model’s performance is dependent on image quality but is nonetheless comparable with similar attempts at ML based segmentation reported in literature. With high quality images, the model has achieved an F_1_ score as high as 0.928, whereas a lower quality image lowers the F_1_ score to 0.689. For the application explored in this study, the model performs sufficiently to analyze fibril cross-sections with similar agreement with ground truth compared to individual human reviewers with the added benefit of removing user bias from the analysis process. Rapid iteration through image sets with large numbers of fibrils made the image analysis faster and more efficient than manual methods, even with the use of software aids like ImageJ-FIJI.

This workflow allows for quick, accurate segmentation of features from grayscale images that facilitates relevant analyses of the ECM or other components in any biological tissue system. Furthermore, this model’s applicability spans beyond biological imaging. Any discipline requiring feature detection and consistent edge definition from any imaging mode can benefit from FIRM, improving the reliability and consistency of feature quantification and the speed at which these measurements are acquired. Many other tools have been developed to aid in feature quantification but fail to address the challenges presented in electron microscopy analysis including user bias, lack of color staining for feature identification, ambiguous feature edges from poor imaging conditions, and contrast variability between images that makes batch thresholding inconsistent. FIRM addresses these concerns and can accurately identify and segment fibrils without human bias while only requiring a small training set of annotated grayscale images.

Future studies will be aimed at quantifying image quality using signal to noise ratio and determining a threshold for minimum required image quality for reliable classification. Further analysis with larger training data sets (>10 images) and increased number of decision trees will be performed to increase accuracy and broaden the applicability of FIRM for scenarios where large training sets are available.

## Supporting information

S1 File Access to FIRM code. (DOCX)

S1 Dataset All raw data. (ZIP)

S2 Dataset All training and testing images. (ZIP)
