# Supplementary material for: FIRM image analysis: A machine learning workflow for quantifying extracellular matrix components from electron microscopy images
Source: PLoS One. 2025 Feb 6;20(2):e0312196. doi: 10.1371/journal.pone.0312196 (PMC11801620; doi:10.1371/journal.pone.0312196)
Supplement: S1 File — (DOCX) [file pone.0312196.s001.docx]

**Supporting Information**

The FIRM image analysis code and all training and test image sets can be accessed on Github at <https://github.com/ngiglio2/FIRM-image-analysis>.
