# Supplementary figures and images for: FIRM image analysis: A machine learning workflow for quantifying extracellular matrix components from electron microscopy images
Source: PLoS One. 2025 Feb 6;20(2):e0312196. doi: 10.1371/journal.pone.0312196 (PMC11801620; doi:10.1371/journal.pone.0312196)

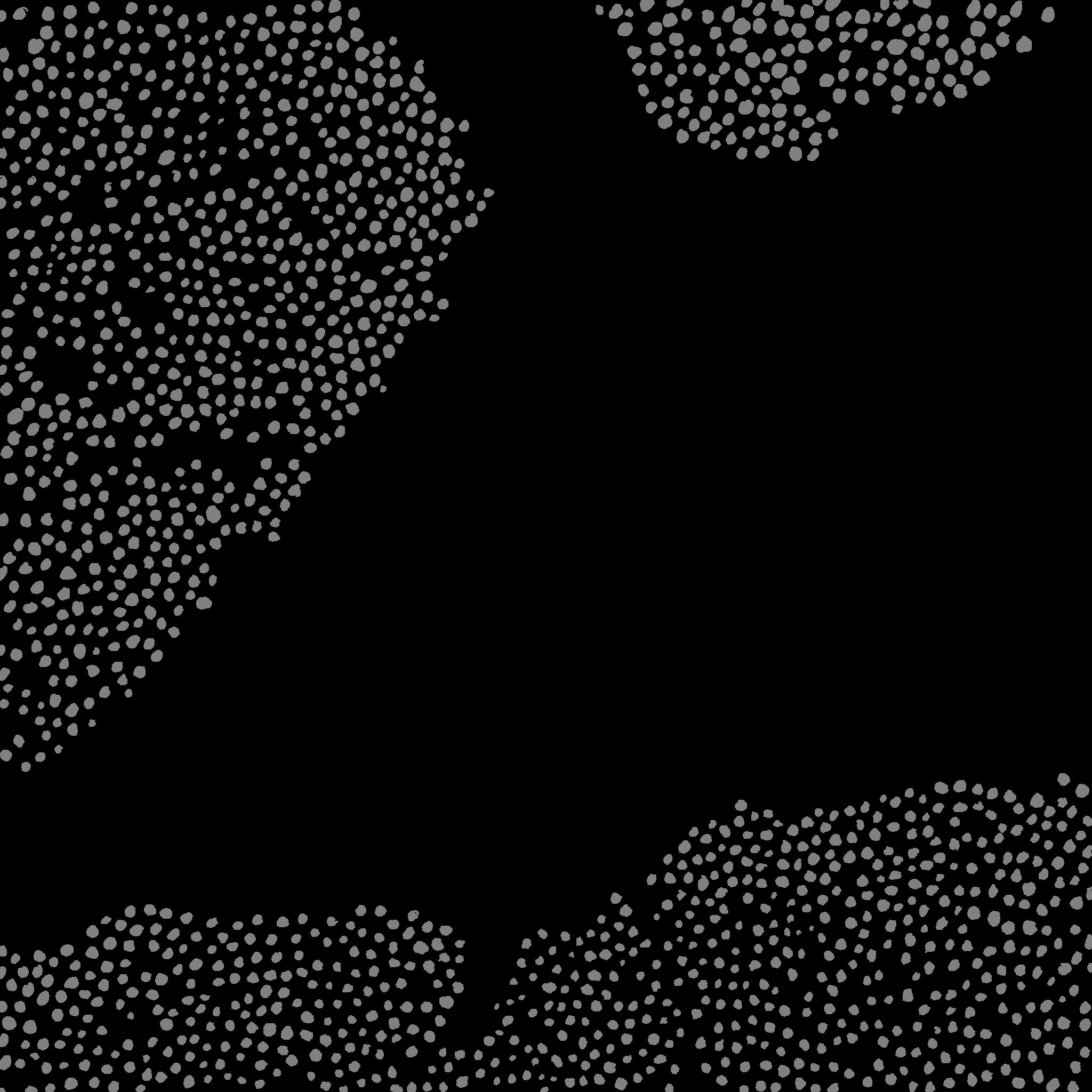

Supplement: S1 Dataset — (ZIP) [file pone.0312196.s002.zip › S2 Dataset/annotations/human 1/US1000XP_4000X_2970_Fibrils.ome.jpg]

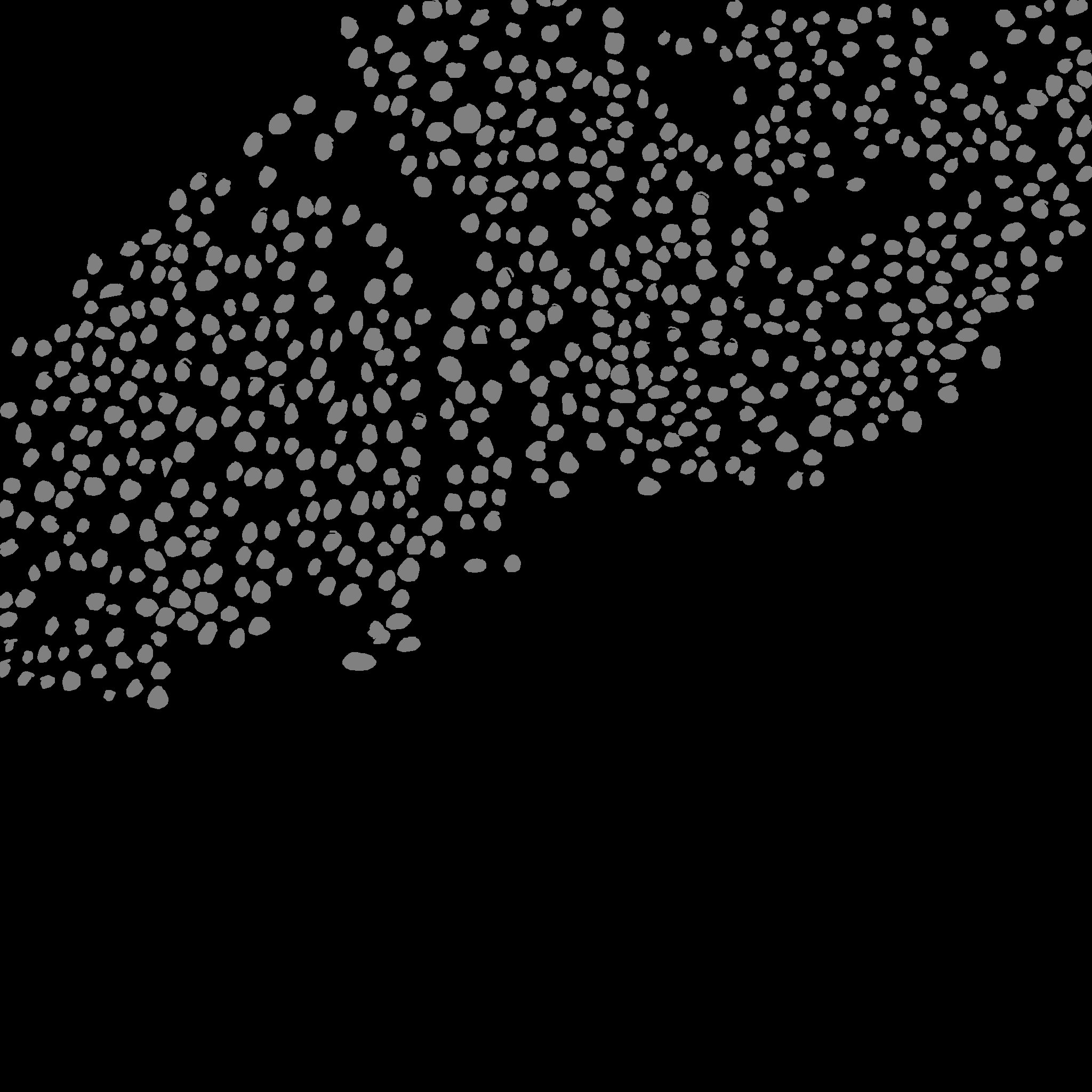

Supplement: S1 Dataset — (ZIP) [file pone.0312196.s002.zip › S2 Dataset/annotations/human 1/US1000XP_4000X_3773_Fibrils.ome.jpg]

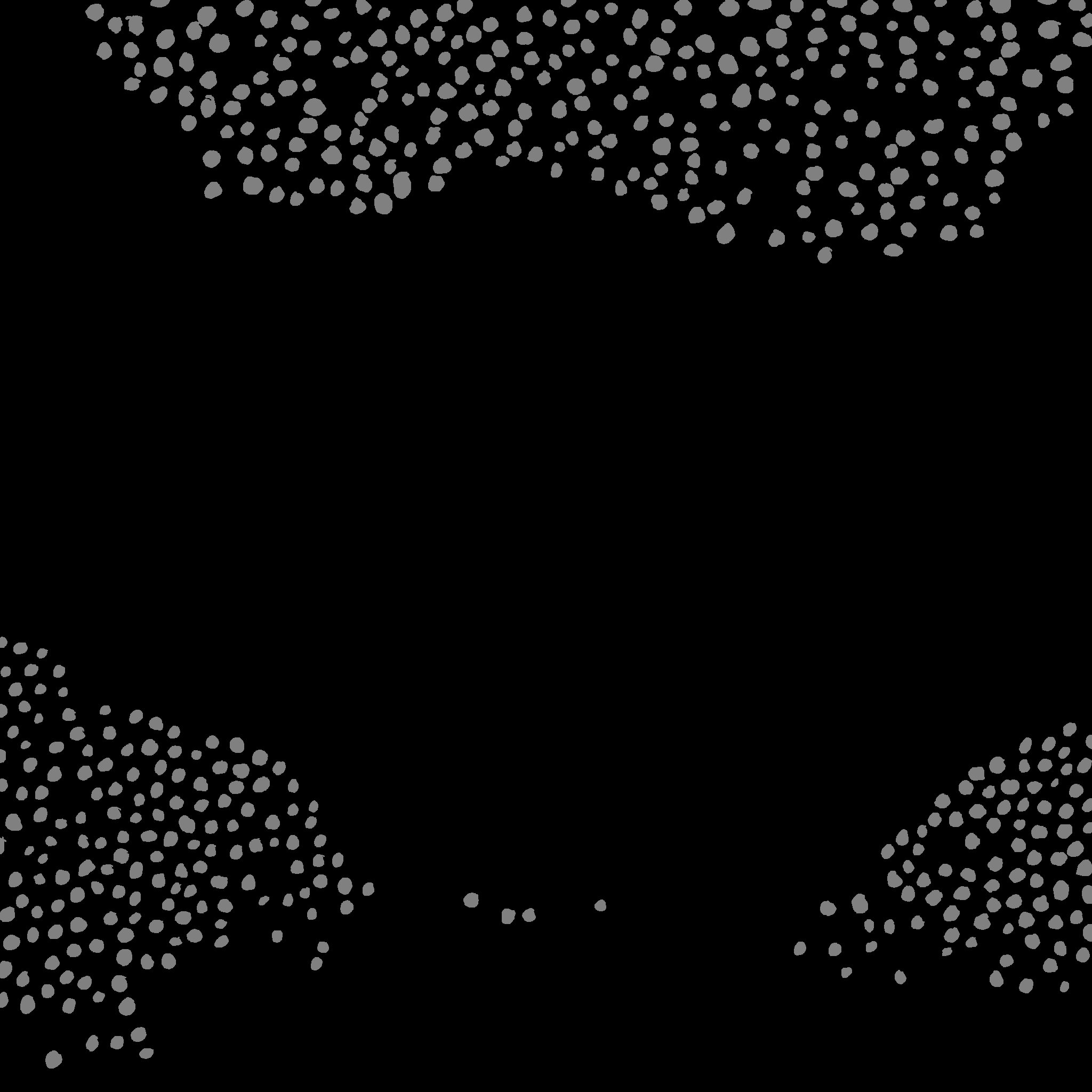

Supplement: S1 Dataset — (ZIP) [file pone.0312196.s002.zip › S2 Dataset/annotations/human 1/US1000XP_5000X_2972_Fibrils.ome.jpg]

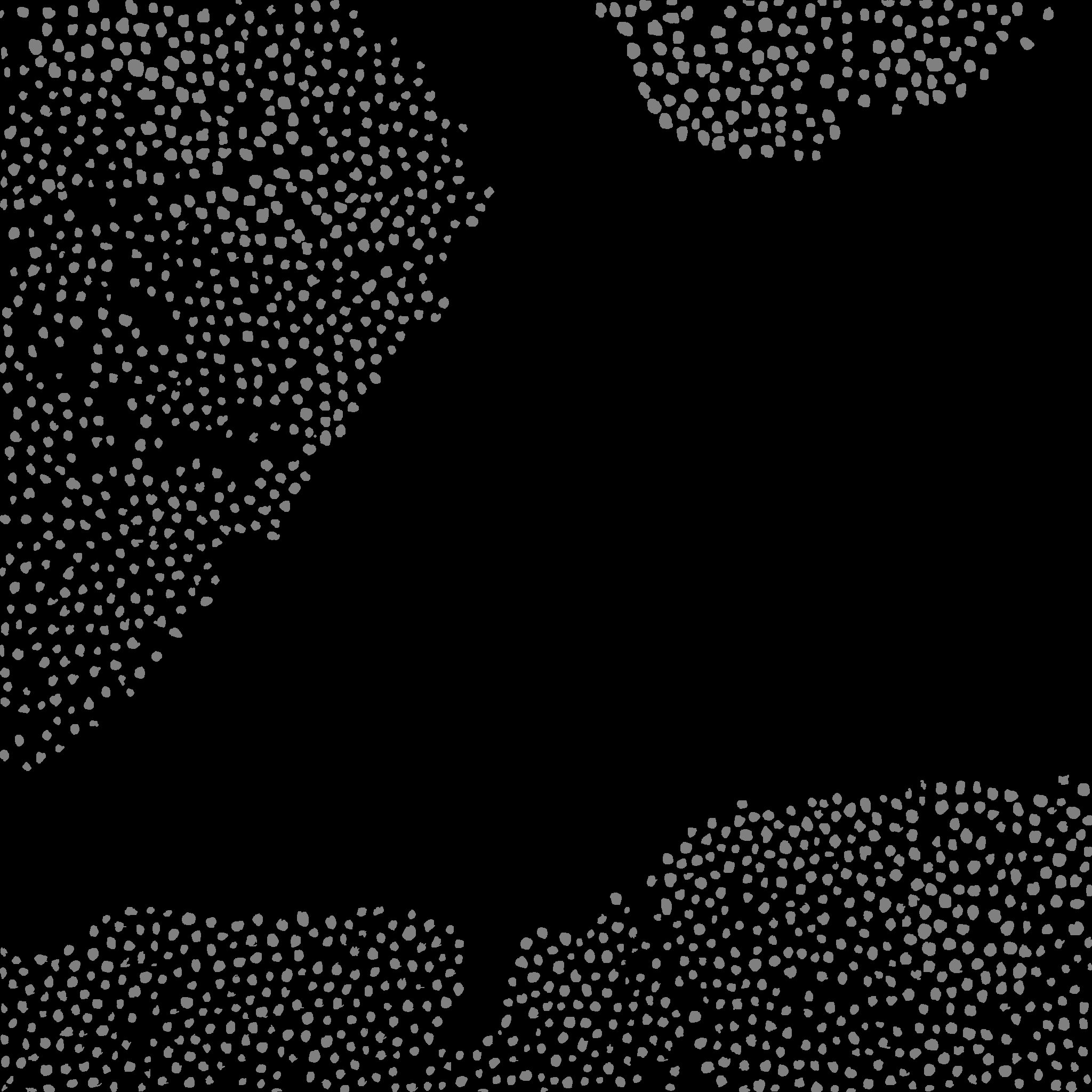

Supplement: S1 Dataset — (ZIP) [file pone.0312196.s002.zip › S2 Dataset/annotations/human 2/US1000XP_4000X_2970_Fibrils.ome.jpg]

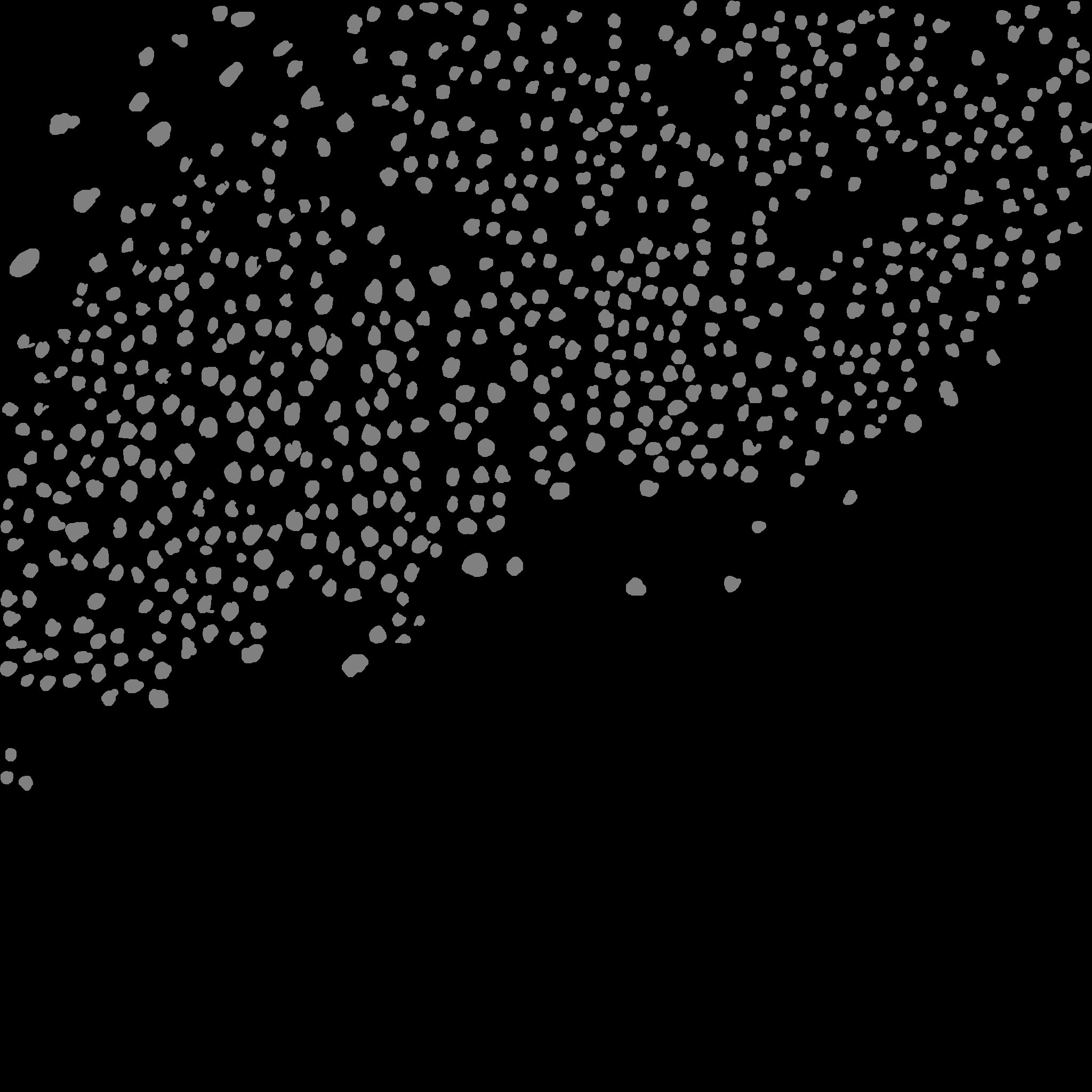

Supplement: S1 Dataset — (ZIP) [file pone.0312196.s002.zip › S2 Dataset/annotations/human 2/US1000XP_4000X_3773_Fibrils.ome.jpg]

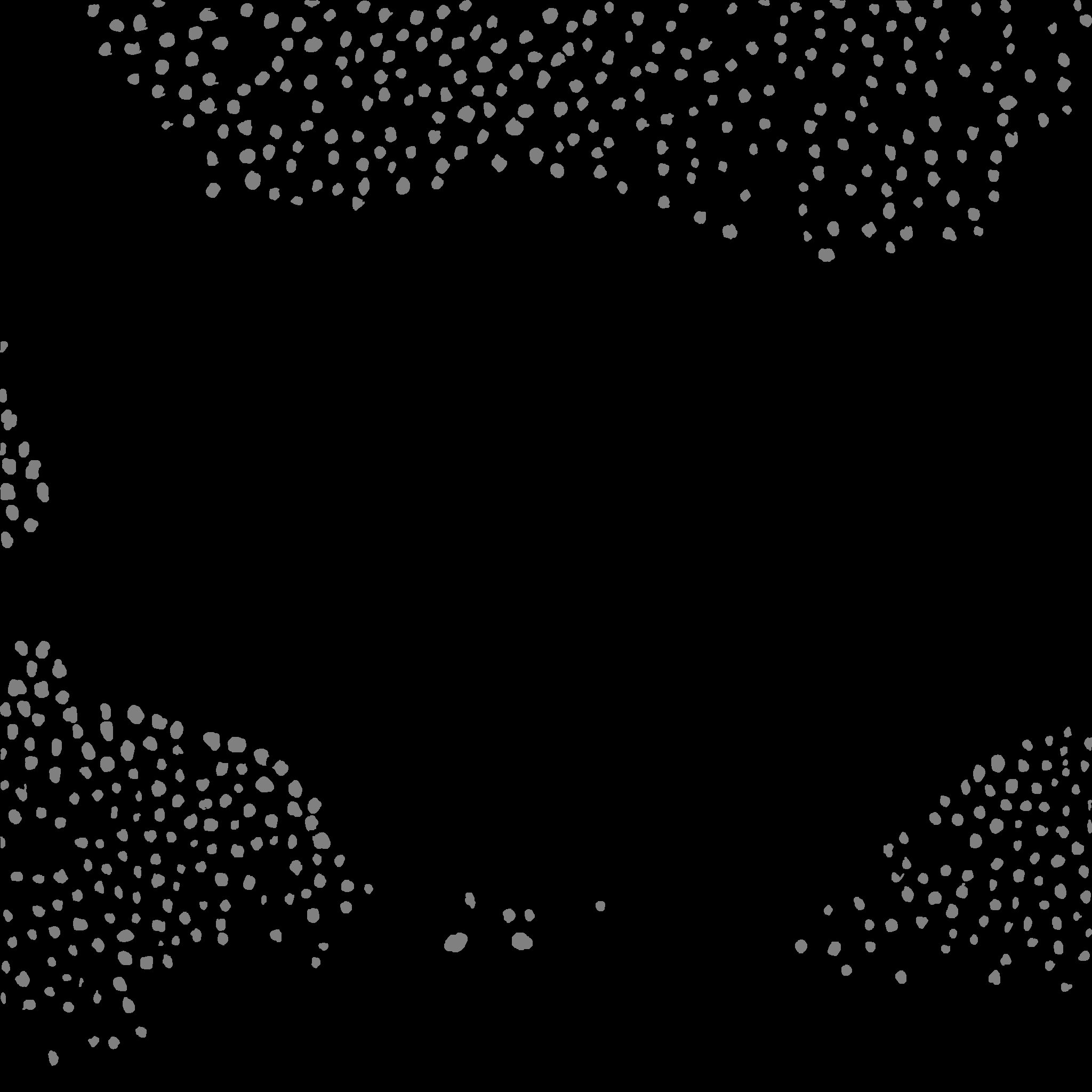

Supplement: S1 Dataset — (ZIP) [file pone.0312196.s002.zip › S2 Dataset/annotations/human 2/US1000XP_5000X_2972_Fibrils.ome.jpg]

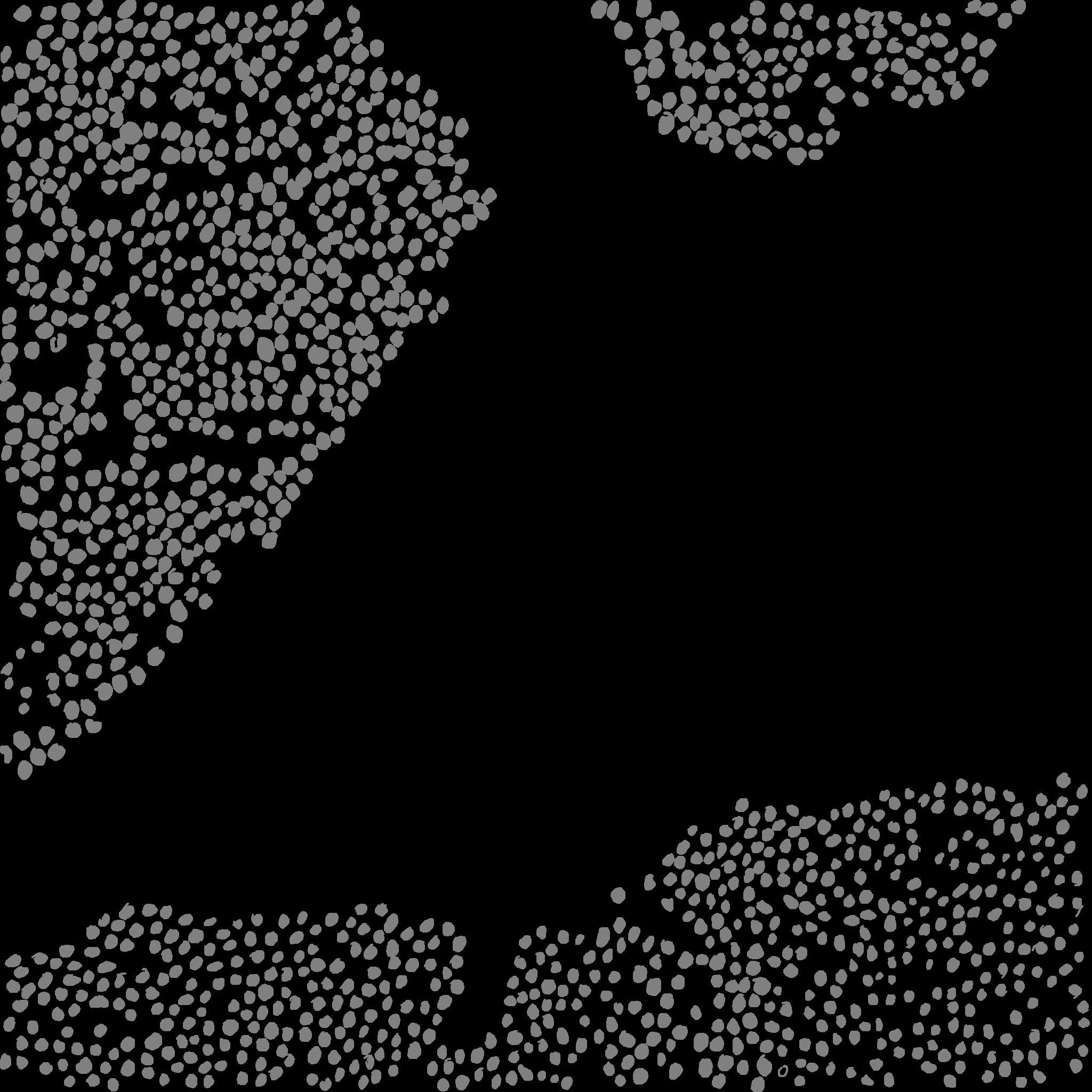

Supplement: S1 Dataset — (ZIP) [file pone.0312196.s002.zip › S2 Dataset/annotations/human 3/US1000XP_4000X_2970_fibril.ome.jpg]

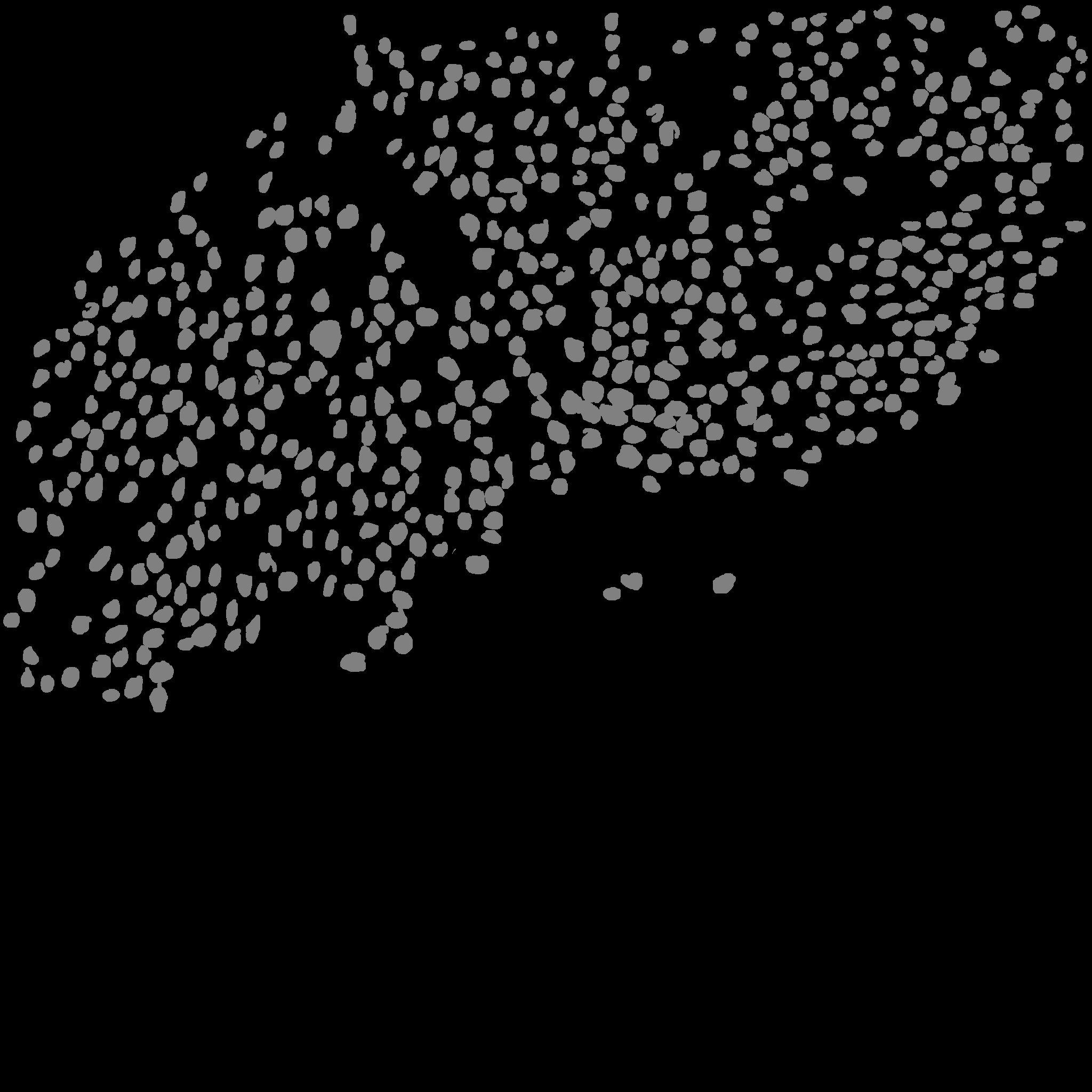

Supplement: S1 Dataset — (ZIP) [file pone.0312196.s002.zip › S2 Dataset/annotations/human 3/US1000XP_4000X_3773_fibril.ome.jpg]

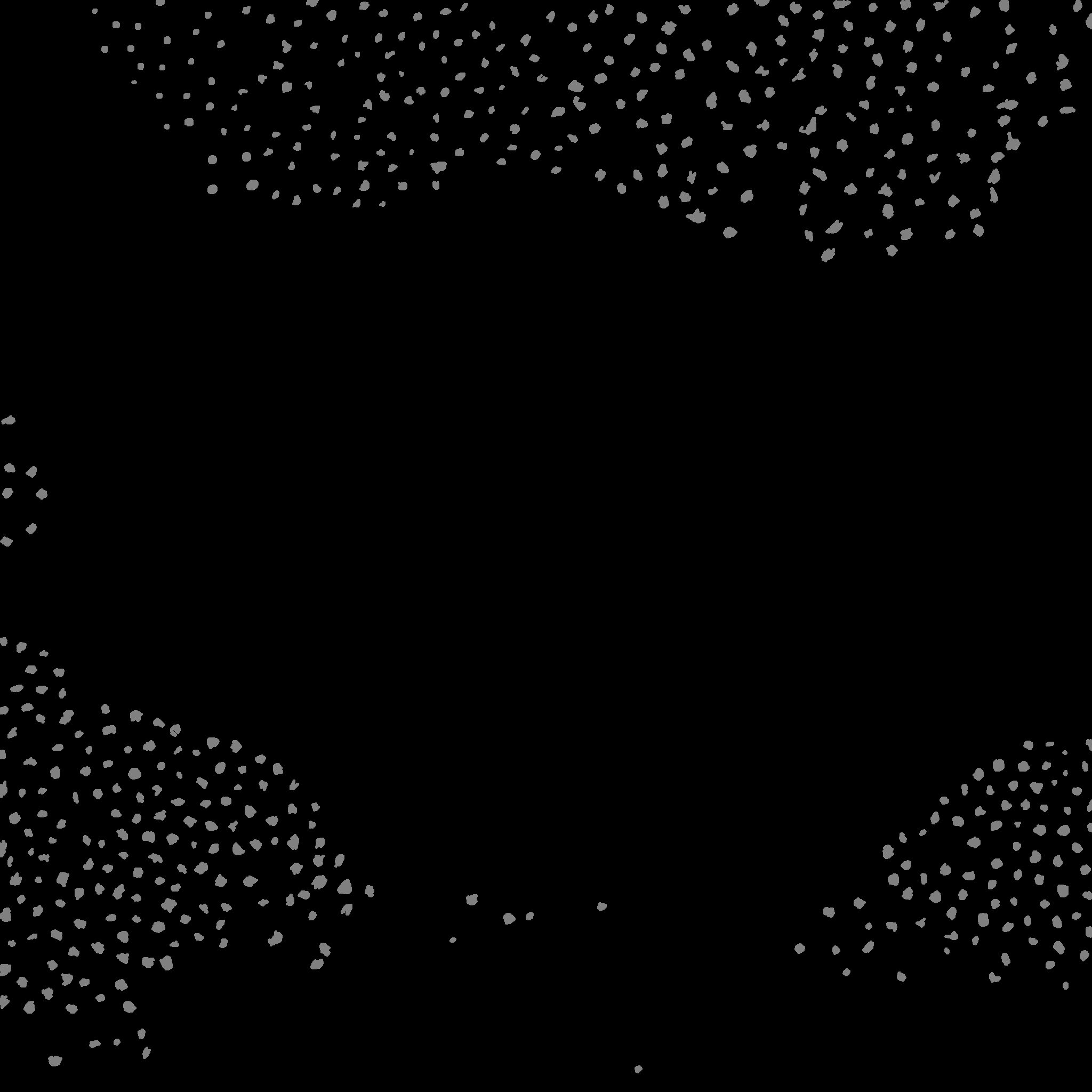

Supplement: S1 Dataset — (ZIP) [file pone.0312196.s002.zip › S2 Dataset/annotations/human 3/US1000XP_5000X_2972_fibril.ome.jpg]

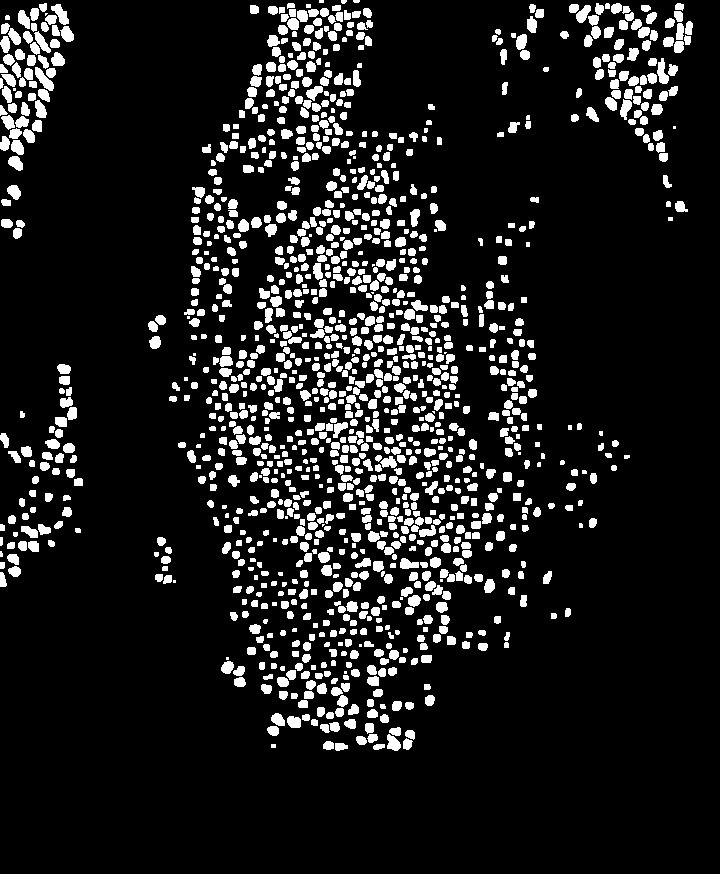

Supplement: S1 Dataset — (ZIP) [file pone.0312196.s002.zip › S2 Dataset/FIRM_increased training set study/FIRM Masks with 10 training images/final.jpgNTG_E8_3_Jun_3_2024_022_16_cleaned.jpg]

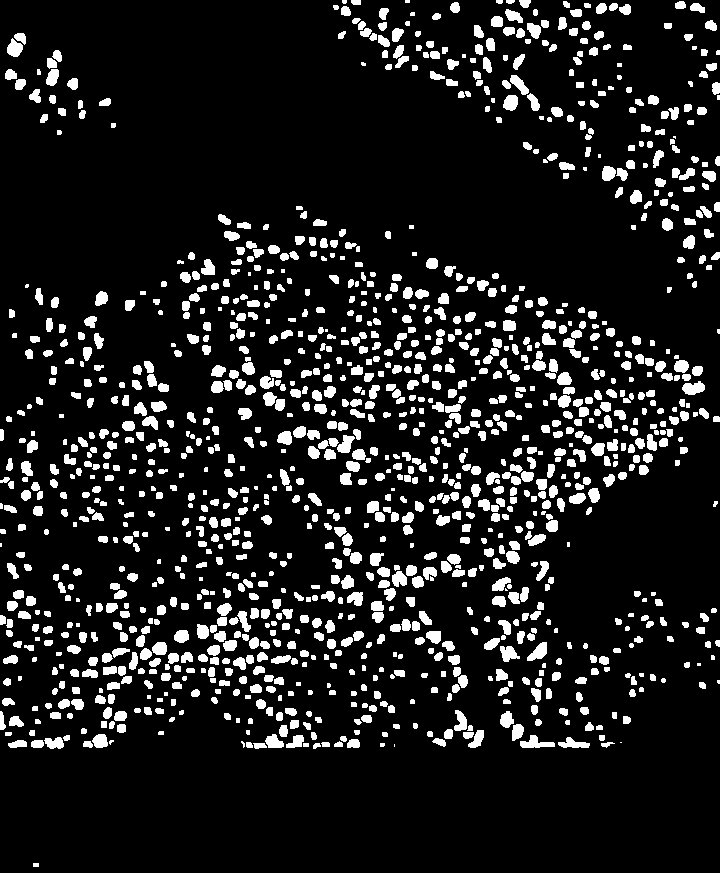

Supplement: S1 Dataset — (ZIP) [file pone.0312196.s002.zip › S2 Dataset/FIRM_increased training set study/FIRM Masks with 10 training images/final.jpgS5_E1_21Feb24_011_16_cleaned.jpg]

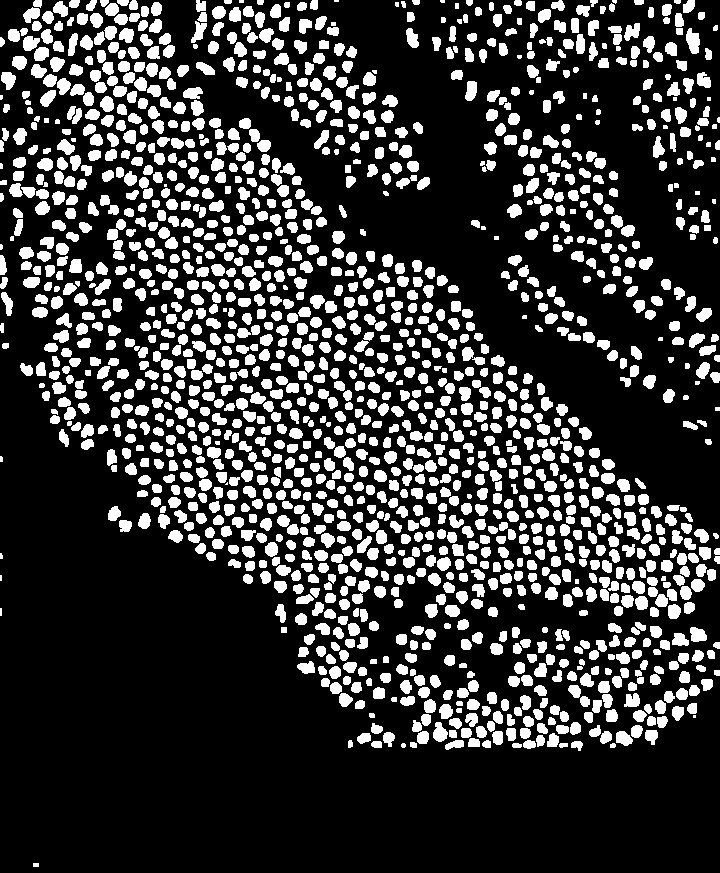

Supplement: S1 Dataset — (ZIP) [file pone.0312196.s002.zip › S2 Dataset/FIRM_increased training set study/FIRM Masks with 10 training images/final.jpgS8_C6_21Feb24_010_16_cleaned.jpg]

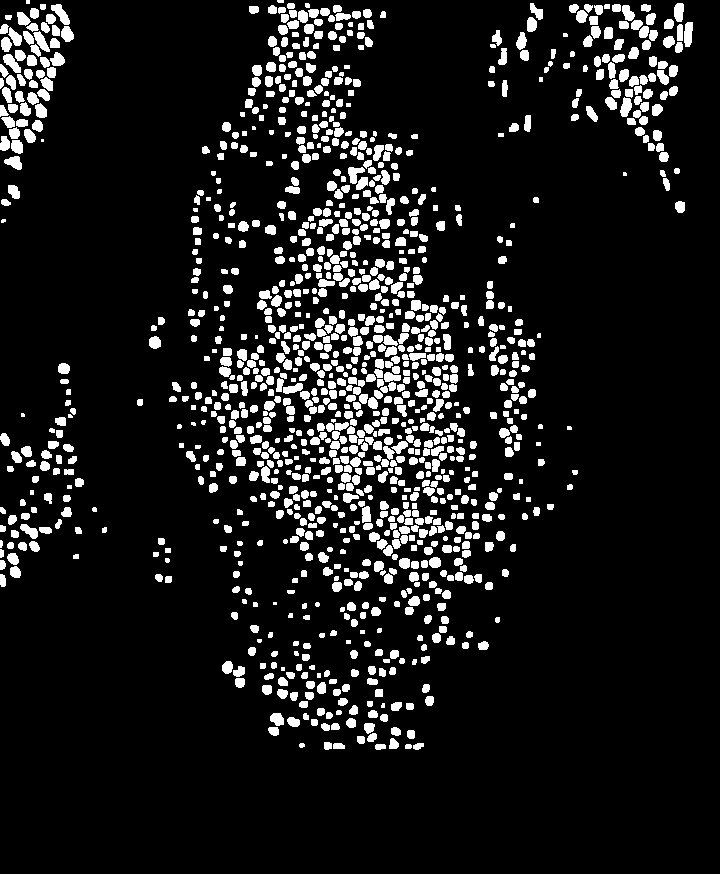

Supplement: S1 Dataset — (ZIP) [file pone.0312196.s002.zip › S2 Dataset/FIRM_increased training set study/FIRM Masks with 7 training images/final.jpgNTG_E8_3_Jun_3_2024_022_16_cleaned.jpg]

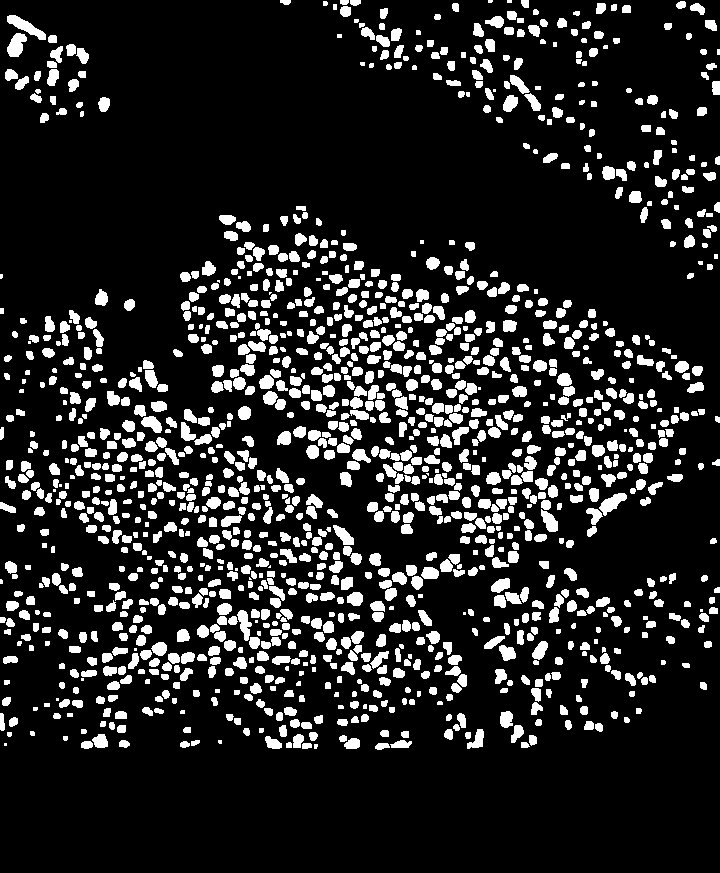

Supplement: S1 Dataset — (ZIP) [file pone.0312196.s002.zip › S2 Dataset/FIRM_increased training set study/FIRM Masks with 7 training images/final.jpgS5_E1_21Feb24_011_16_cleaned.jpg]

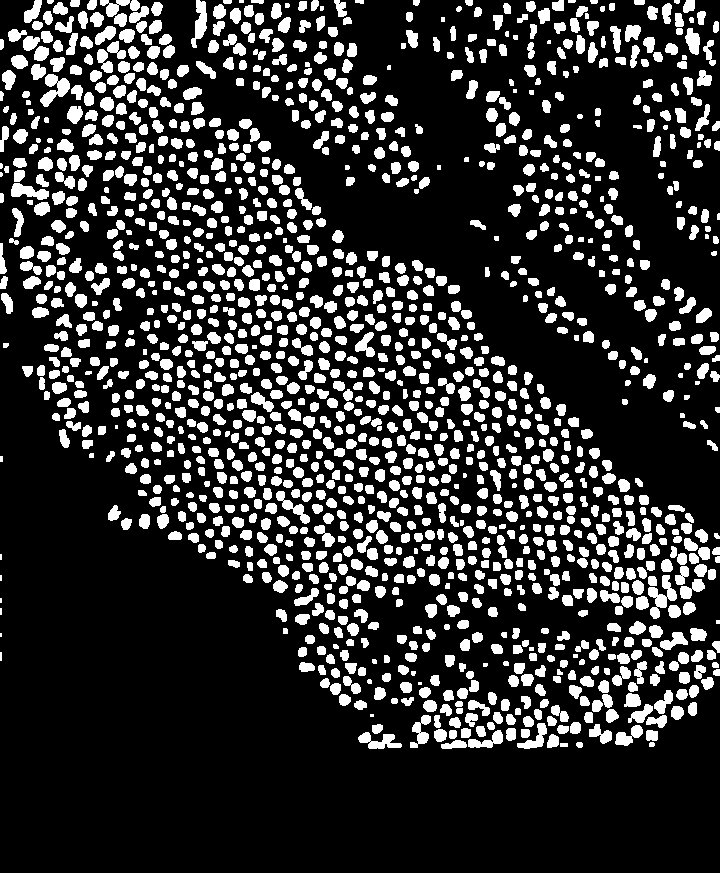

Supplement: S1 Dataset — (ZIP) [file pone.0312196.s002.zip › S2 Dataset/FIRM_increased training set study/FIRM Masks with 7 training images/final.jpgS8_C6_21Feb24_010_16_cleaned.jpg]

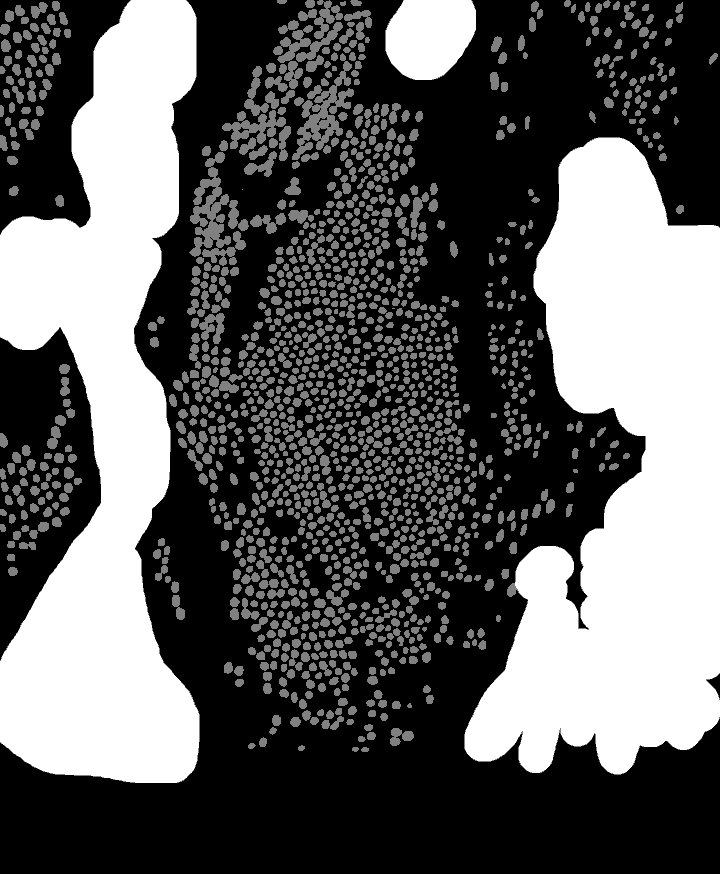

Supplement: S1 Dataset — (ZIP) [file pone.0312196.s002.zip › S2 Dataset/FIRM_increased training set study/Increased Training Set/Training masks/Result of NTG_E8_3_Jun_3_2024_022_16_Fibrils.ome.jpg]

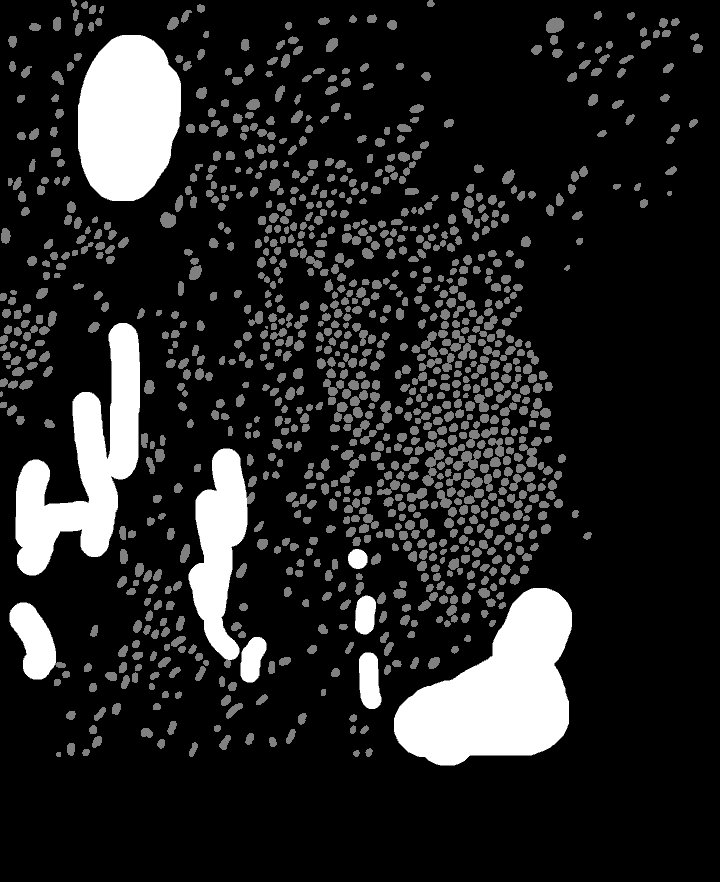

Supplement: S1 Dataset — (ZIP) [file pone.0312196.s002.zip › S2 Dataset/FIRM_increased training set study/Increased Training Set/Training masks/Result of NTG_E8_6_3_2024_003_16_background.ome.jpg]

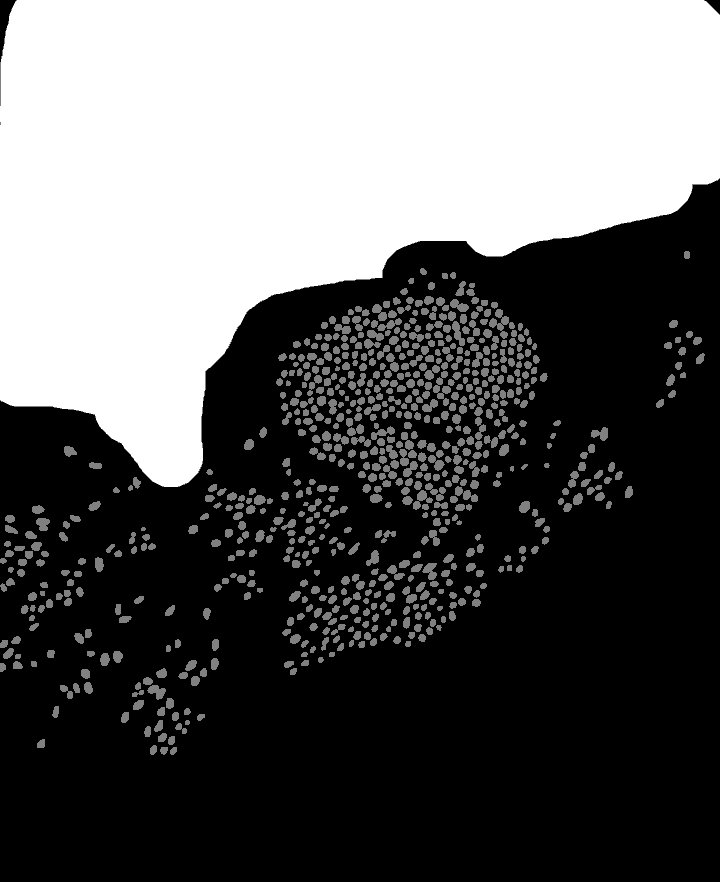

Supplement: S1 Dataset — (ZIP) [file pone.0312196.s002.zip › S2 Dataset/FIRM_increased training set study/Increased Training Set/Training masks/Result of NTG_E8_6_3_2024_008_16_background.ome.jpg]

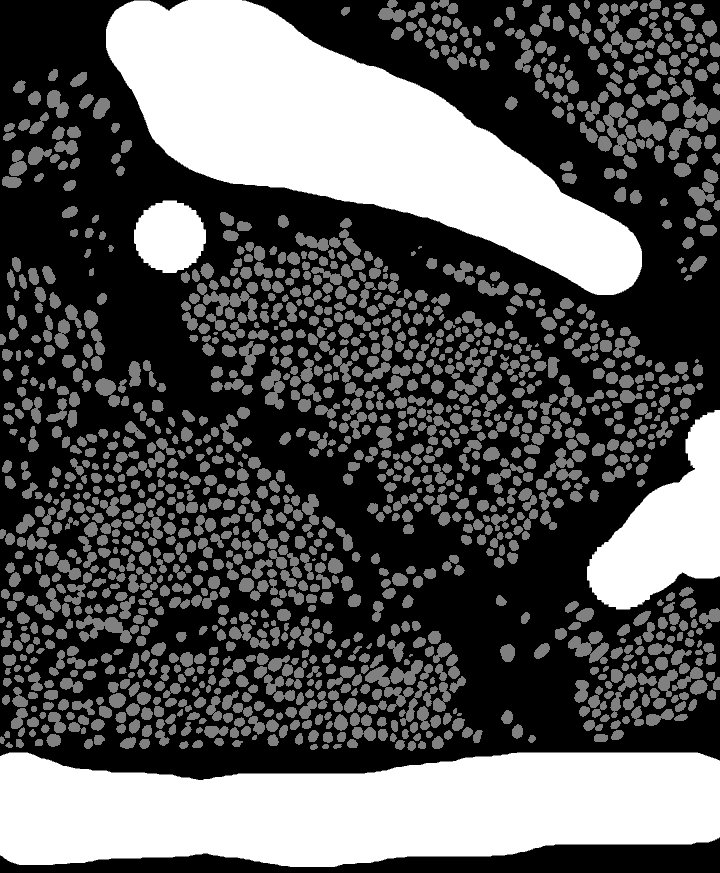

Supplement: S1 Dataset — (ZIP) [file pone.0312196.s002.zip › S2 Dataset/FIRM_increased training set study/Increased Training Set/Training masks/Result of S5_E1_21Feb24_011_16_background.ome.jpg]

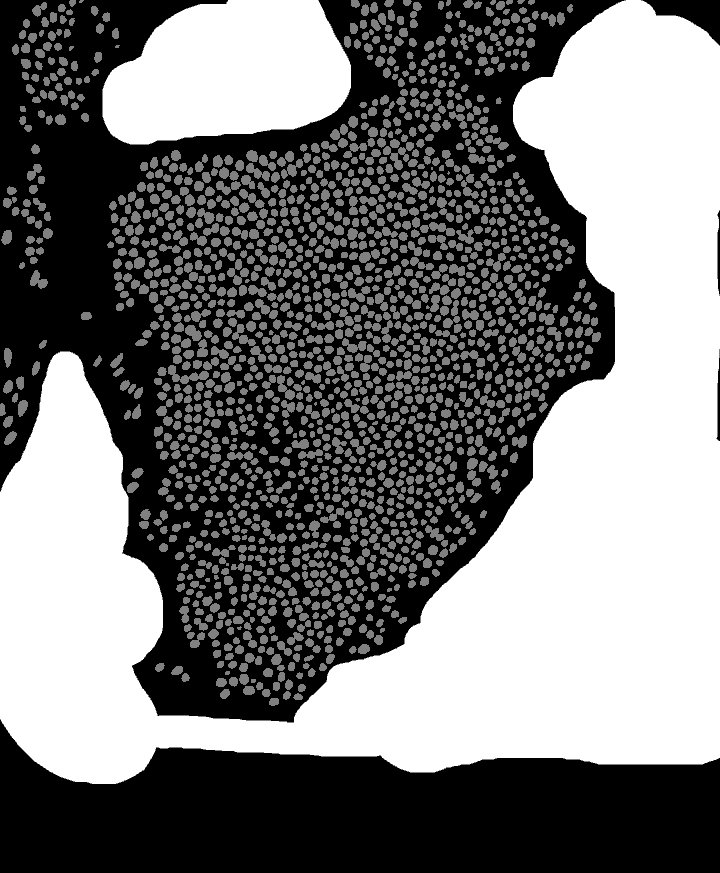

Supplement: S1 Dataset — (ZIP) [file pone.0312196.s002.zip › S2 Dataset/FIRM_increased training set study/Increased Training Set/Training masks/Result of S6_A1_020224_011_16_background.ome.jpg]

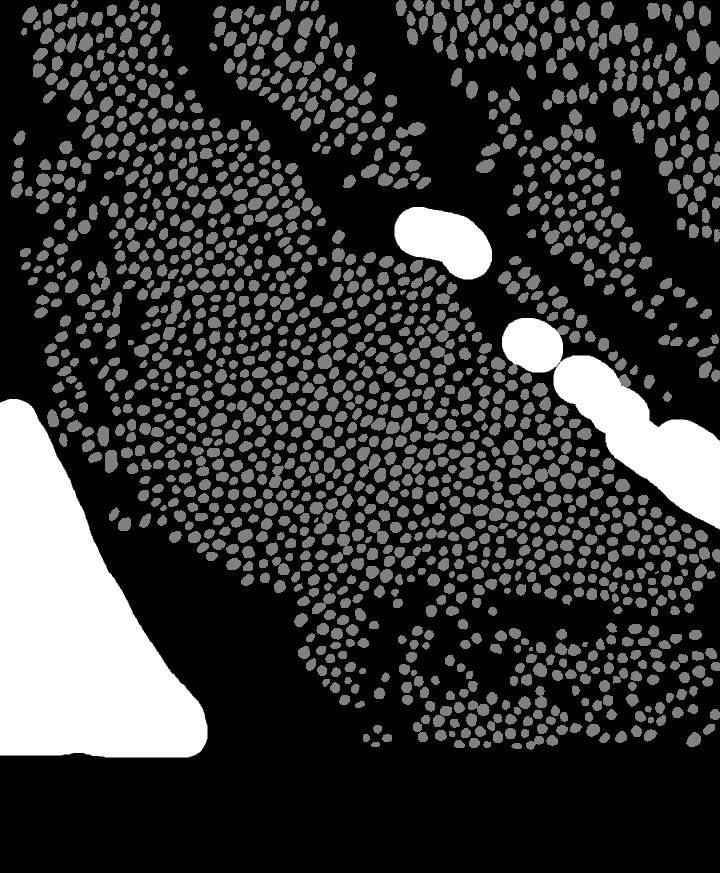

Supplement: S1 Dataset — (ZIP) [file pone.0312196.s002.zip › S2 Dataset/FIRM_increased training set study/Increased Training Set/Training masks/Result of S8_C6_21Feb24_010_16_background.ome.jpg]

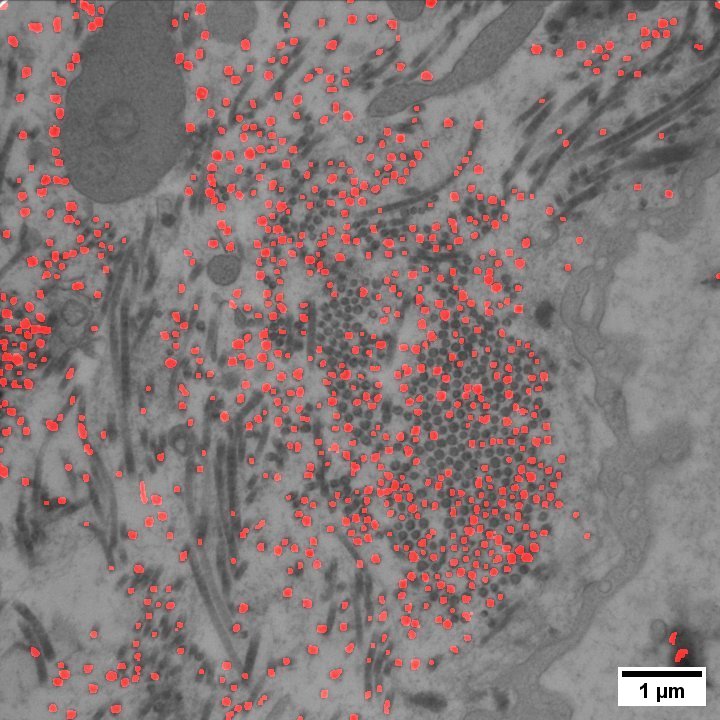

Supplement: S1 Dataset — (ZIP) [file pone.0312196.s002.zip › S2 Dataset/FIRM_scarce training study/100%/Composite_E8.jpg]

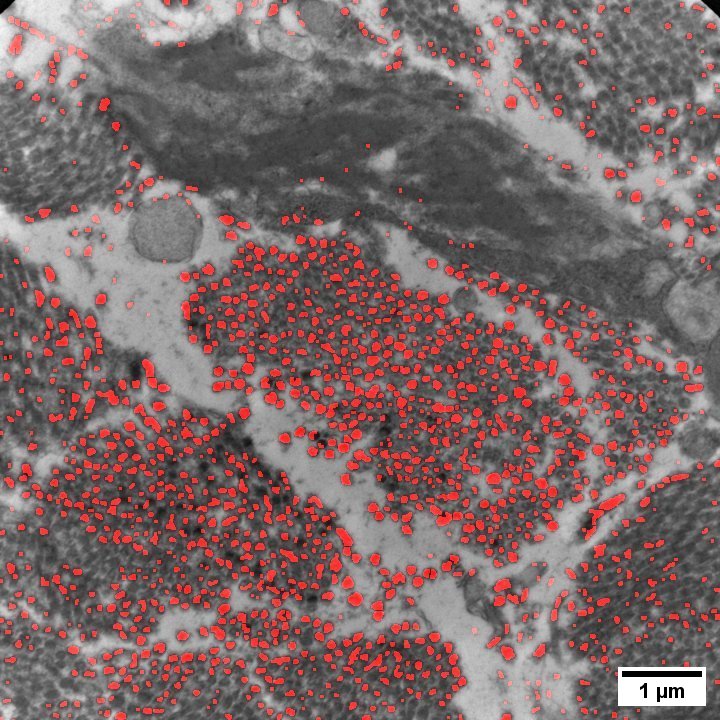

Supplement: S1 Dataset — (ZIP) [file pone.0312196.s002.zip › S2 Dataset/FIRM_scarce training study/100%/Composite_S5.jpg]

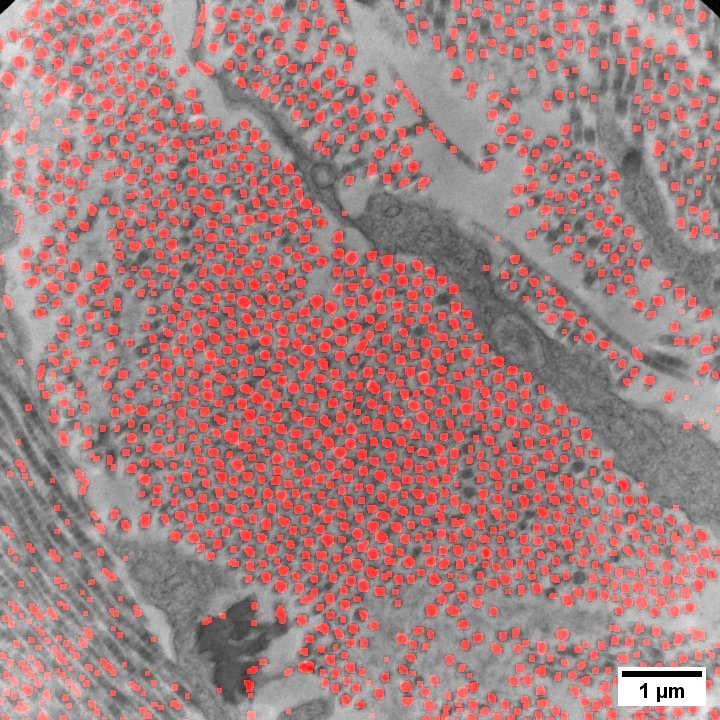

Supplement: S1 Dataset — (ZIP) [file pone.0312196.s002.zip › S2 Dataset/FIRM_scarce training study/100%/Composite_S8.jpg]

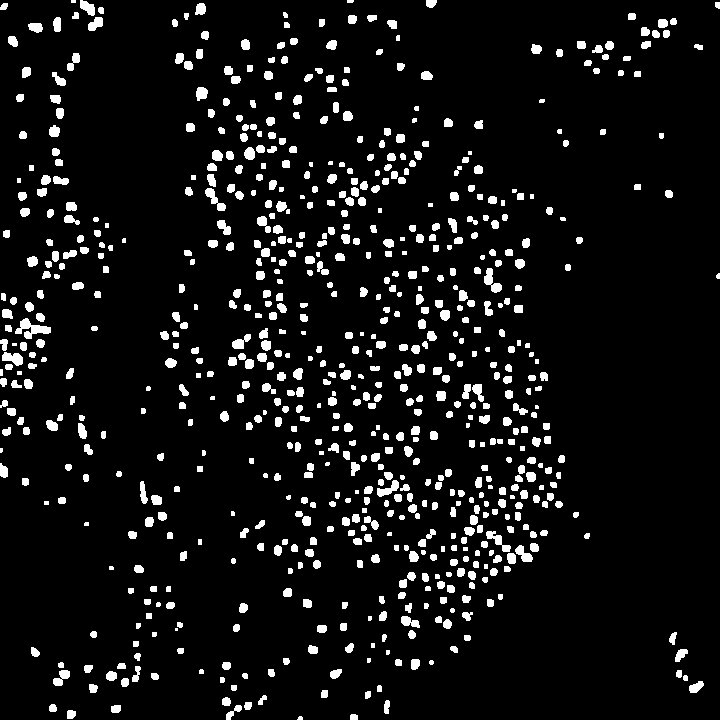

Supplement: S1 Dataset — (ZIP) [file pone.0312196.s002.zip › S2 Dataset/FIRM_scarce training study/100%/final.jpgNTG_E8_6_3_2024_003_16_clean.jpg]

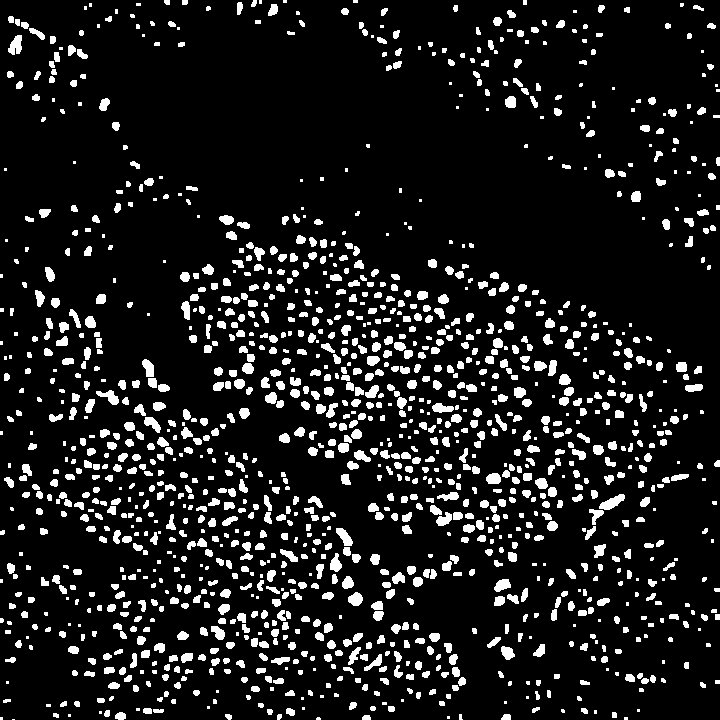

Supplement: S1 Dataset — (ZIP) [file pone.0312196.s002.zip › S2 Dataset/FIRM_scarce training study/100%/final.jpgS5_E1_21Feb24_011_16_cleaned.jpg]

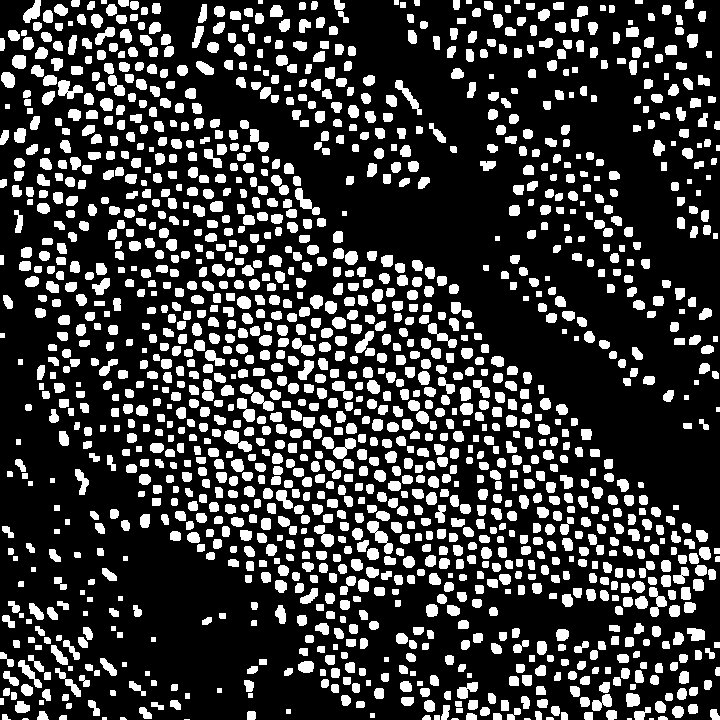

Supplement: S1 Dataset — (ZIP) [file pone.0312196.s002.zip › S2 Dataset/FIRM_scarce training study/100%/final.jpgS8_C6_21Feb24_010_16_cleaned.jpg]

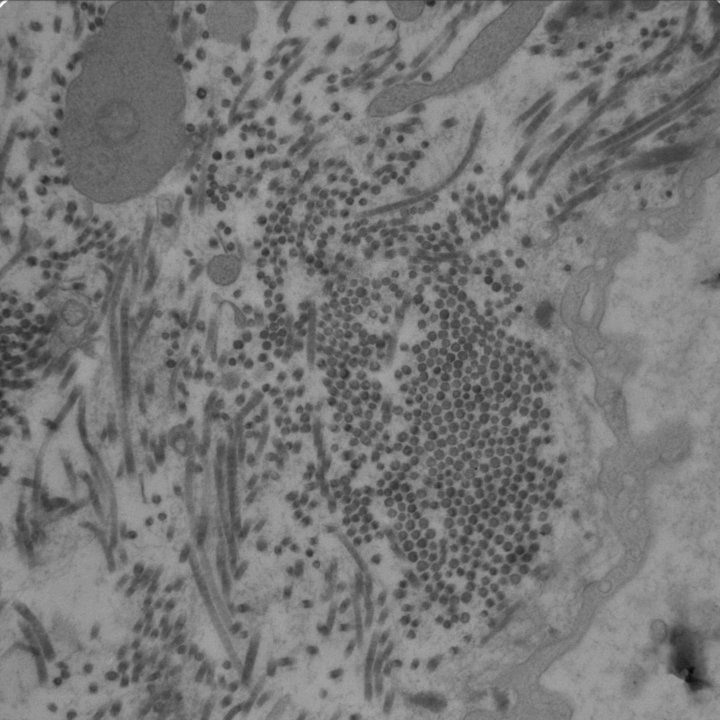

Supplement: S1 Dataset — (ZIP) [file pone.0312196.s002.zip › S2 Dataset/FIRM_scarce training study/100%/NTG_E8_6_3_2024_003_16.jpg]

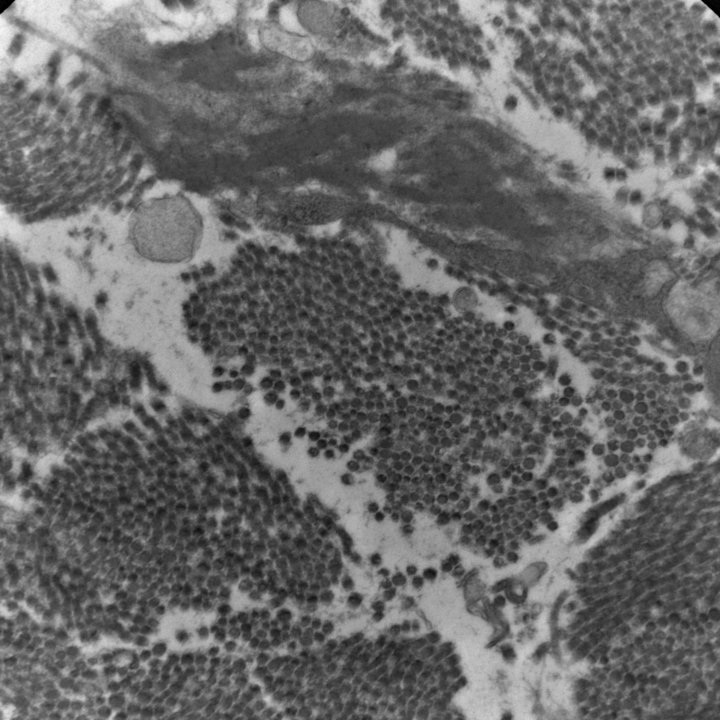

Supplement: S1 Dataset — (ZIP) [file pone.0312196.s002.zip › S2 Dataset/FIRM_scarce training study/100%/S5_E1_21Feb24_011_16.jpg]

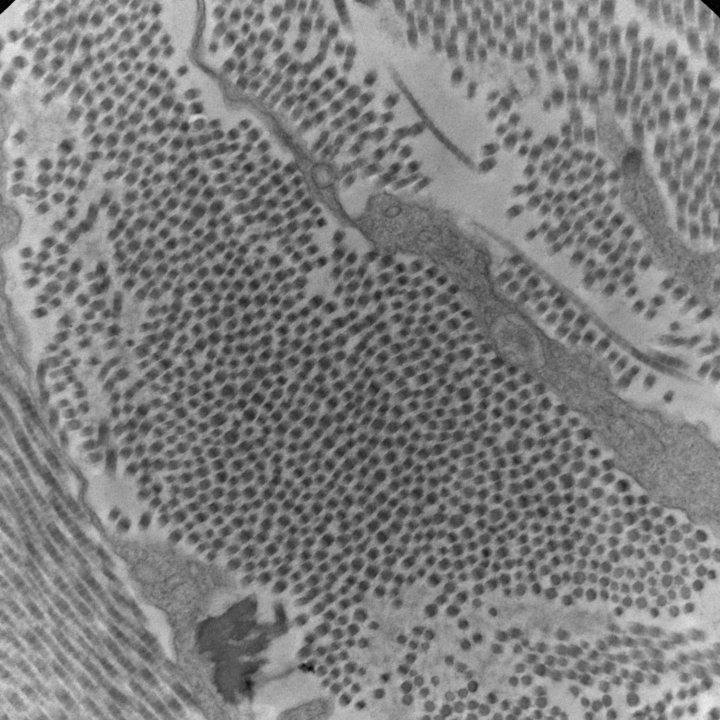

Supplement: S1 Dataset — (ZIP) [file pone.0312196.s002.zip › S2 Dataset/FIRM_scarce training study/100%/S8_C6_21Feb24_010_16.jpg]

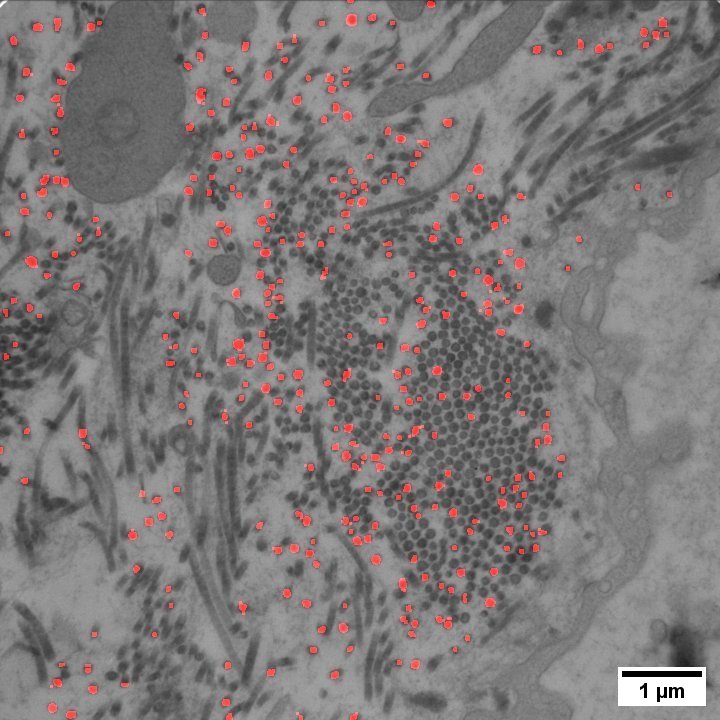

Supplement: S1 Dataset — (ZIP) [file pone.0312196.s002.zip › S2 Dataset/FIRM_scarce training study/25%/Composite_E8.jpg]

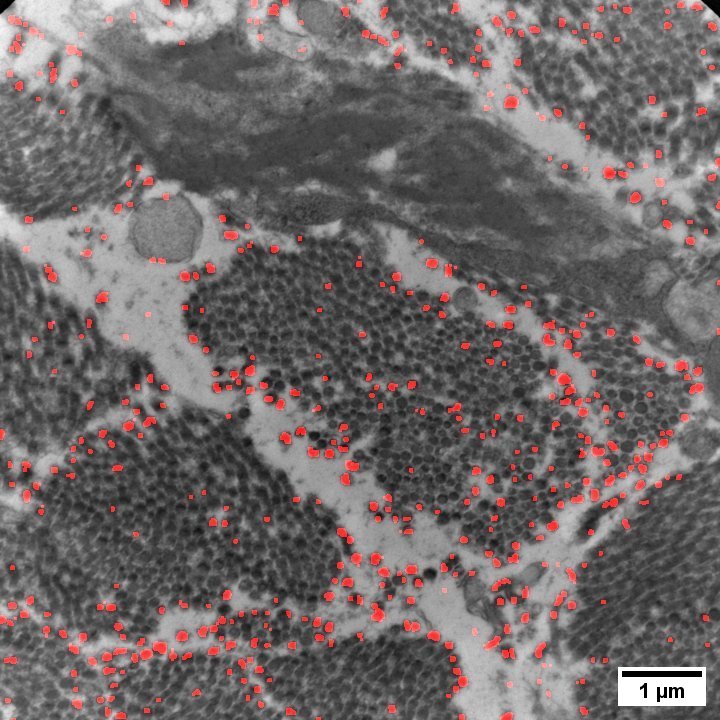

Supplement: S1 Dataset — (ZIP) [file pone.0312196.s002.zip › S2 Dataset/FIRM_scarce training study/25%/Composite_S5.jpg]

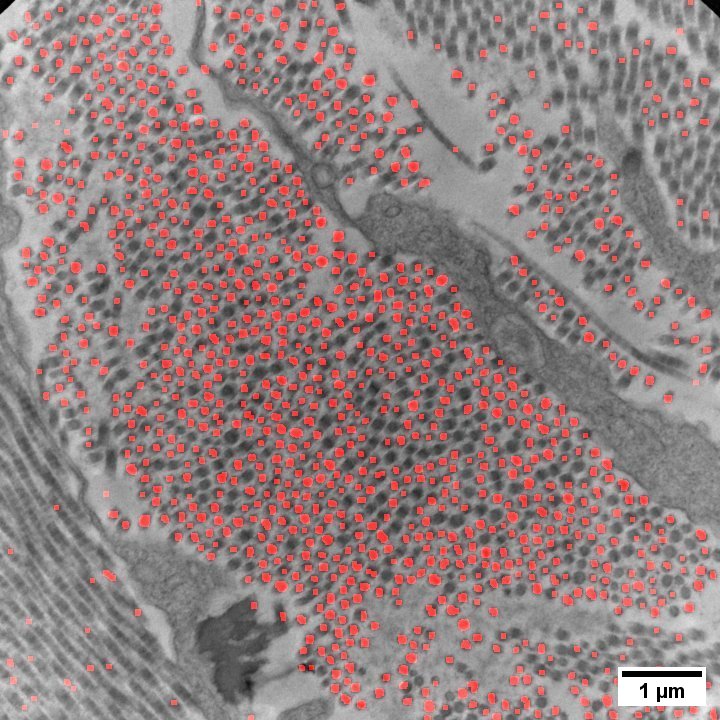

Supplement: S1 Dataset — (ZIP) [file pone.0312196.s002.zip › S2 Dataset/FIRM_scarce training study/25%/Composite_S8.jpg]

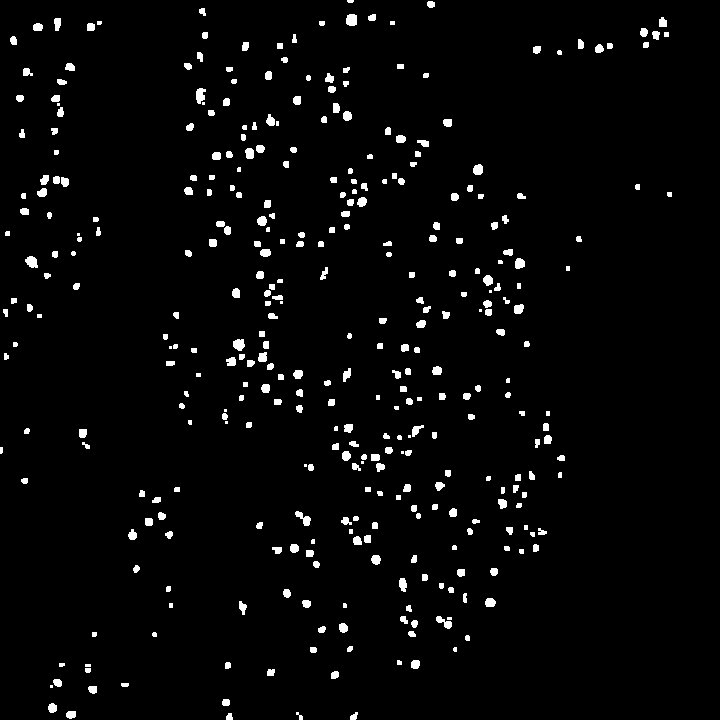

Supplement: S1 Dataset — (ZIP) [file pone.0312196.s002.zip › S2 Dataset/FIRM_scarce training study/25%/final.jpgNTG_E8_6_3_2024_003_16_clean.jpg]

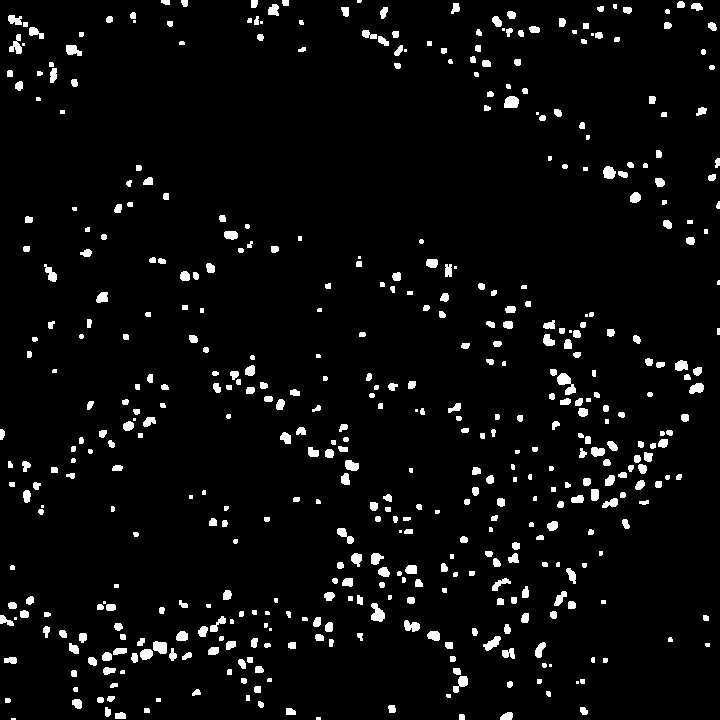

Supplement: S1 Dataset — (ZIP) [file pone.0312196.s002.zip › S2 Dataset/FIRM_scarce training study/25%/final.jpgS5_E1_21Feb24_011_16_clean.jpg]

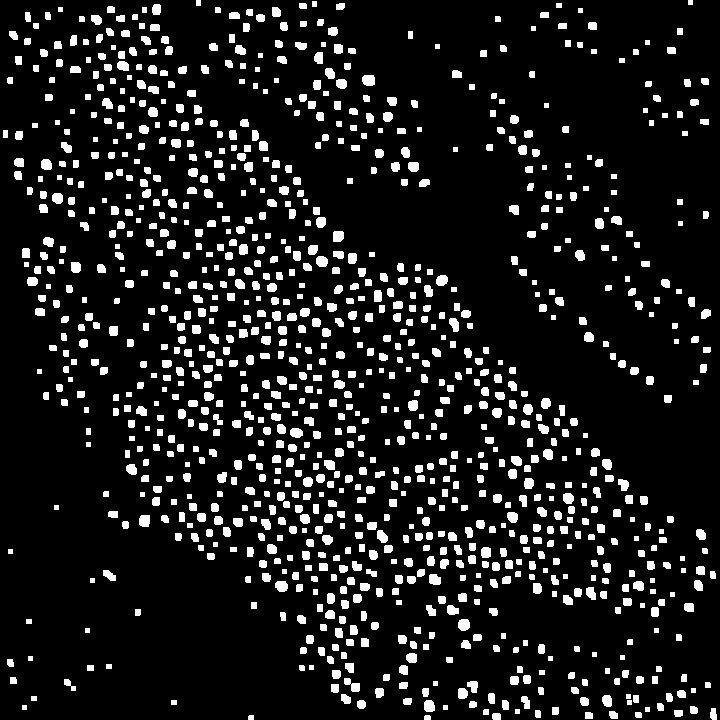

Supplement: S1 Dataset — (ZIP) [file pone.0312196.s002.zip › S2 Dataset/FIRM_scarce training study/25%/final.jpgS8_C6_21Feb24_010_16_clean.jpg]

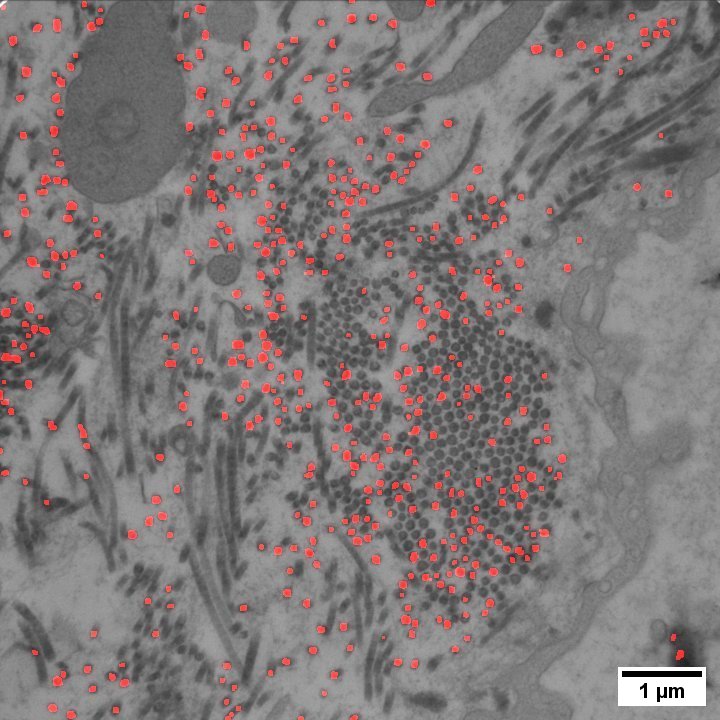

Supplement: S1 Dataset — (ZIP) [file pone.0312196.s002.zip › S2 Dataset/FIRM_scarce training study/50%/Composite_E8.jpg]

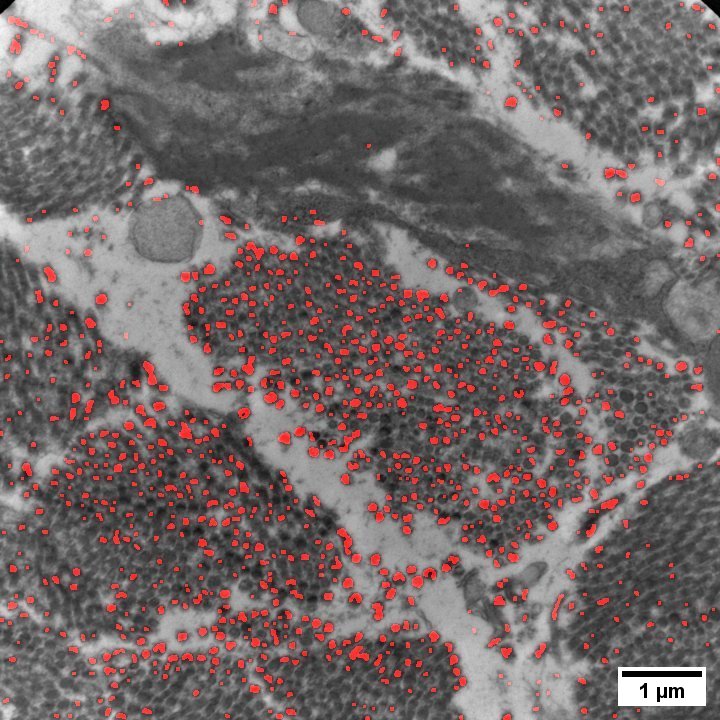

Supplement: S1 Dataset — (ZIP) [file pone.0312196.s002.zip › S2 Dataset/FIRM_scarce training study/50%/Composite_S5.jpg]

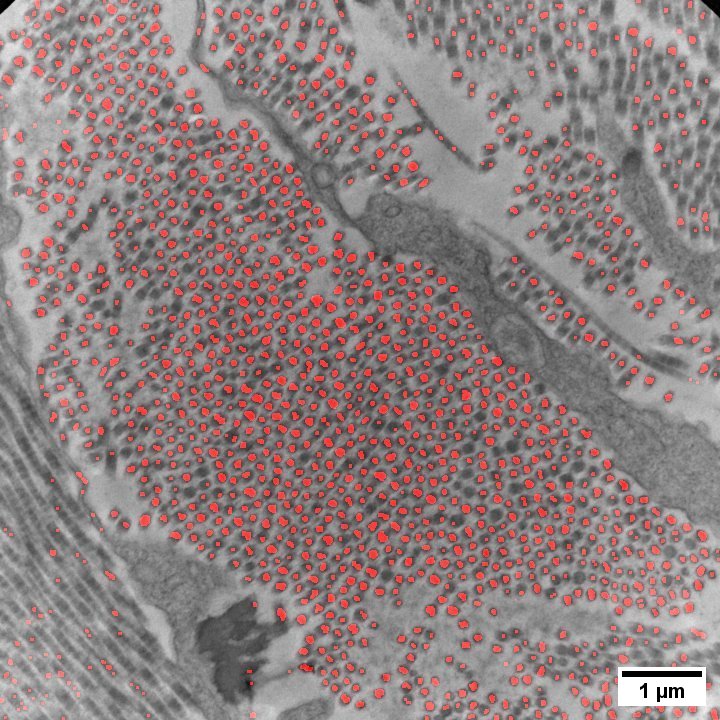

Supplement: S1 Dataset — (ZIP) [file pone.0312196.s002.zip › S2 Dataset/FIRM_scarce training study/50%/Composite_S8.jpg]

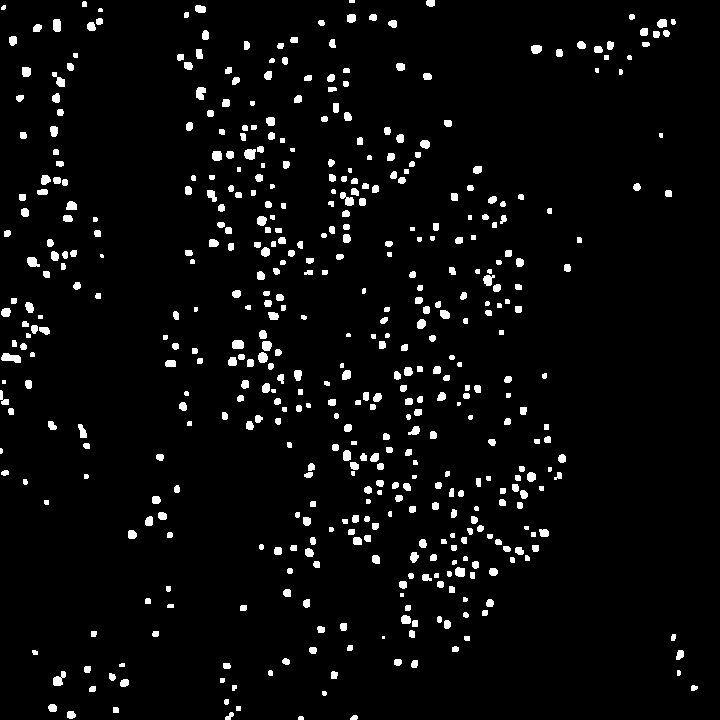

Supplement: S1 Dataset — (ZIP) [file pone.0312196.s002.zip › S2 Dataset/FIRM_scarce training study/50%/final.jpgNTG_E8_6_3_2024_003_16_clean.jpg]

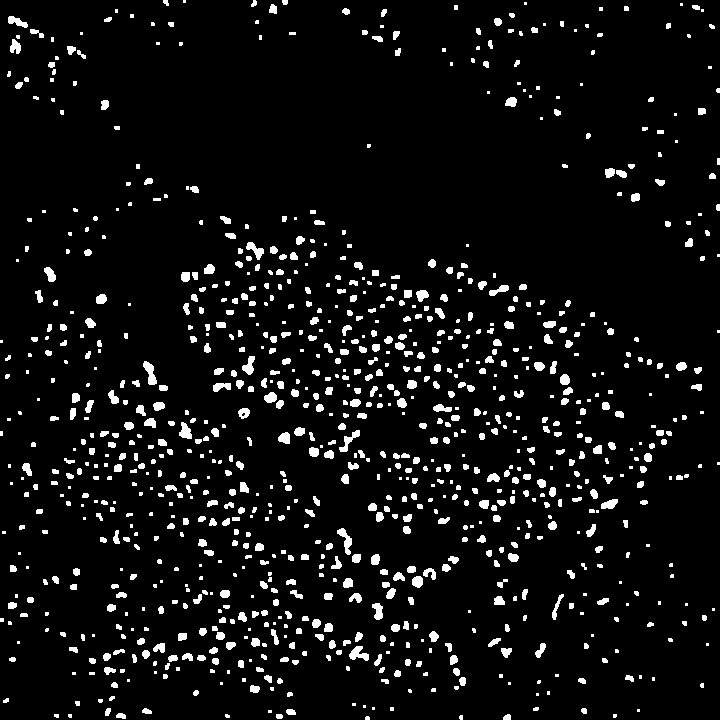

Supplement: S1 Dataset — (ZIP) [file pone.0312196.s002.zip › S2 Dataset/FIRM_scarce training study/50%/final.jpgS5_E1_21Feb24_011_16_clean.jpg]

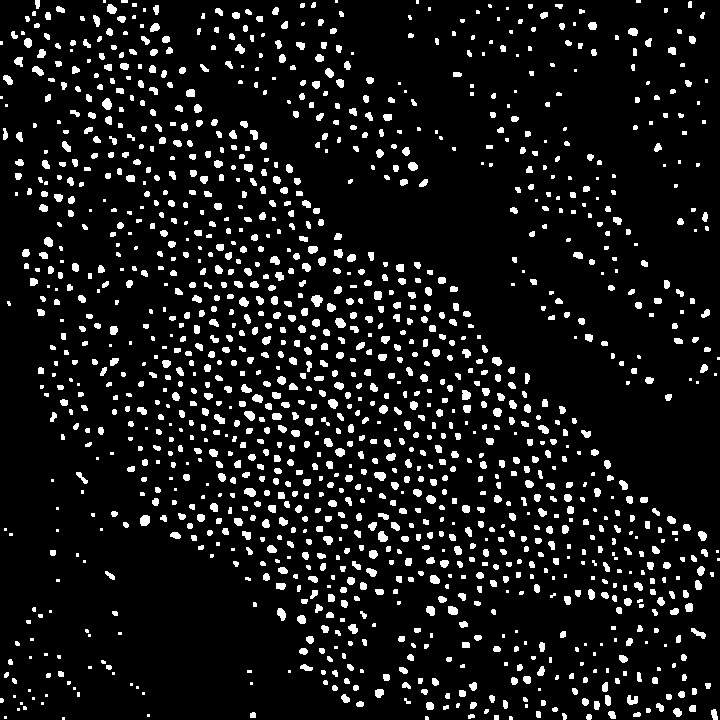

Supplement: S1 Dataset — (ZIP) [file pone.0312196.s002.zip › S2 Dataset/FIRM_scarce training study/50%/final.jpgS8_C6_21Feb24_010_16_clean.jpg]

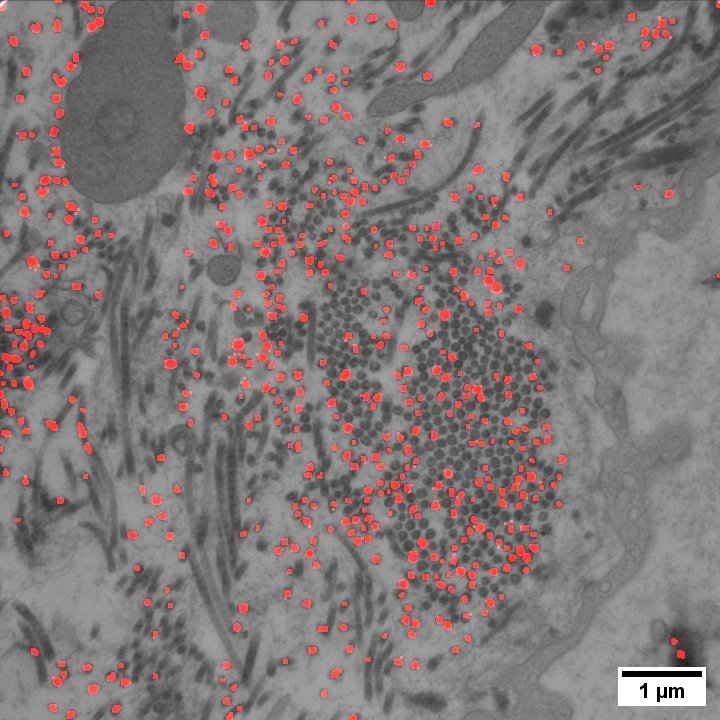

Supplement: S1 Dataset — (ZIP) [file pone.0312196.s002.zip › S2 Dataset/FIRM_scarce training study/75%/Composite_ntgE8.jpg]

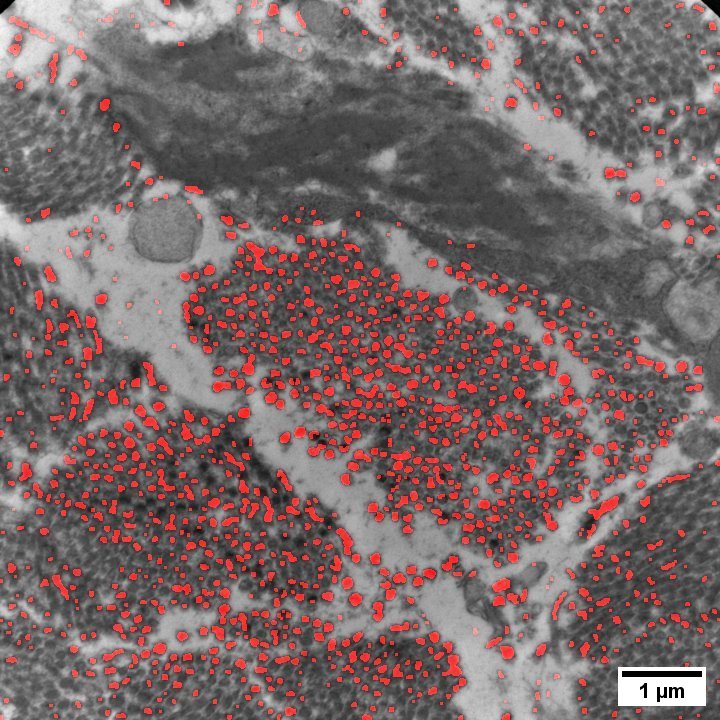

Supplement: S1 Dataset — (ZIP) [file pone.0312196.s002.zip › S2 Dataset/FIRM_scarce training study/75%/Composite_S5.jpg]

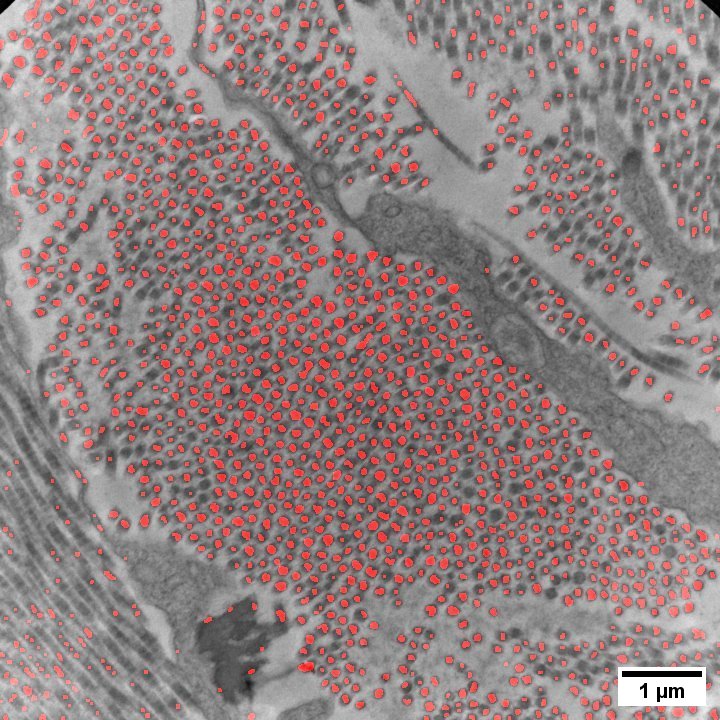

Supplement: S1 Dataset — (ZIP) [file pone.0312196.s002.zip › S2 Dataset/FIRM_scarce training study/75%/Composite_S8.jpg]

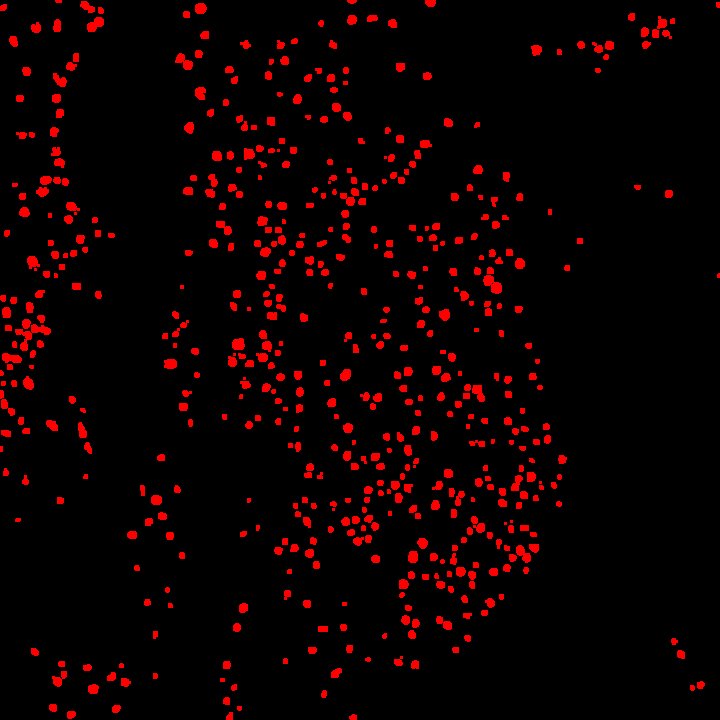

Supplement: S1 Dataset — (ZIP) [file pone.0312196.s002.zip › S2 Dataset/FIRM_scarce training study/75%/final.jpgNTG_E8_6_3_2024_003_16_clEANED.jpg]

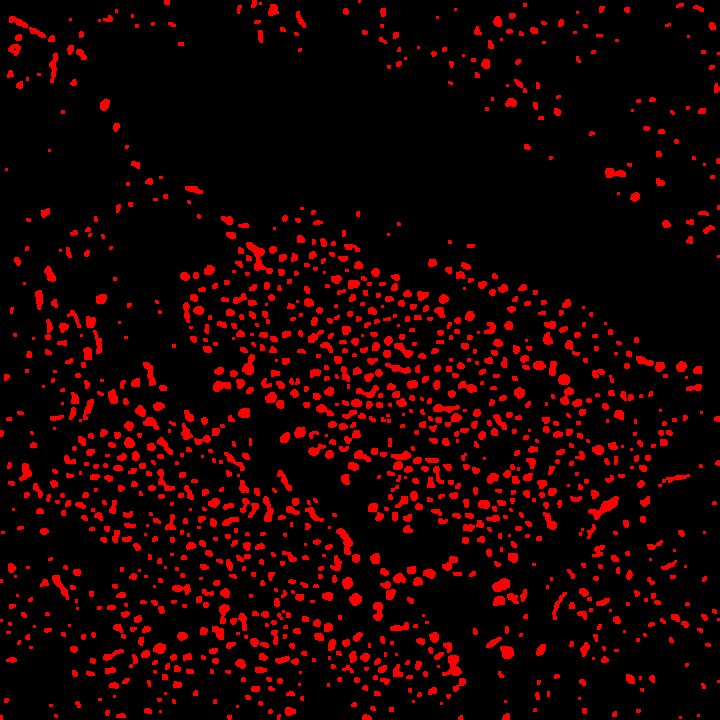

Supplement: S1 Dataset — (ZIP) [file pone.0312196.s002.zip › S2 Dataset/FIRM_scarce training study/75%/final.jpgS5_E1_21Feb24_011_16_cleaned.jpg]

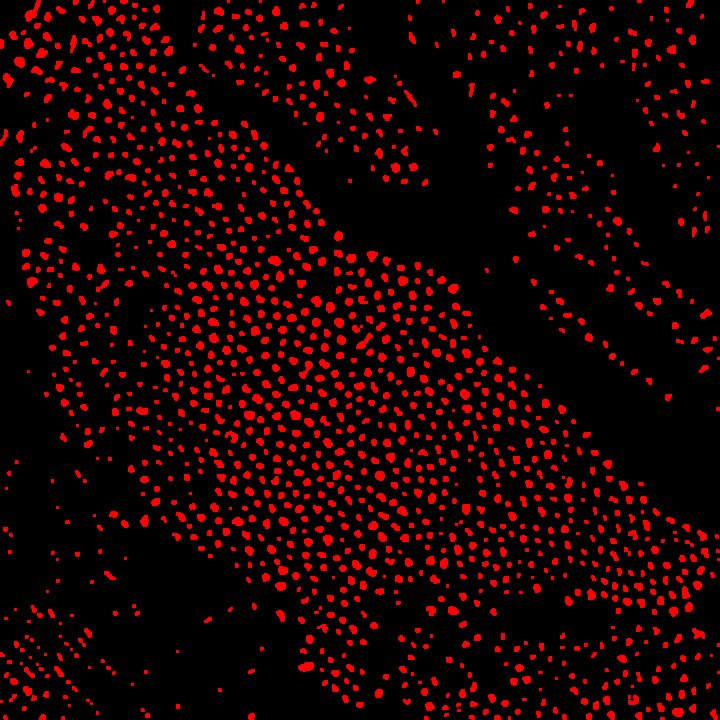

Supplement: S1 Dataset — (ZIP) [file pone.0312196.s002.zip › S2 Dataset/FIRM_scarce training study/75%/final.jpgS8_C6_21Feb24_010_16_cleaned.jpg]

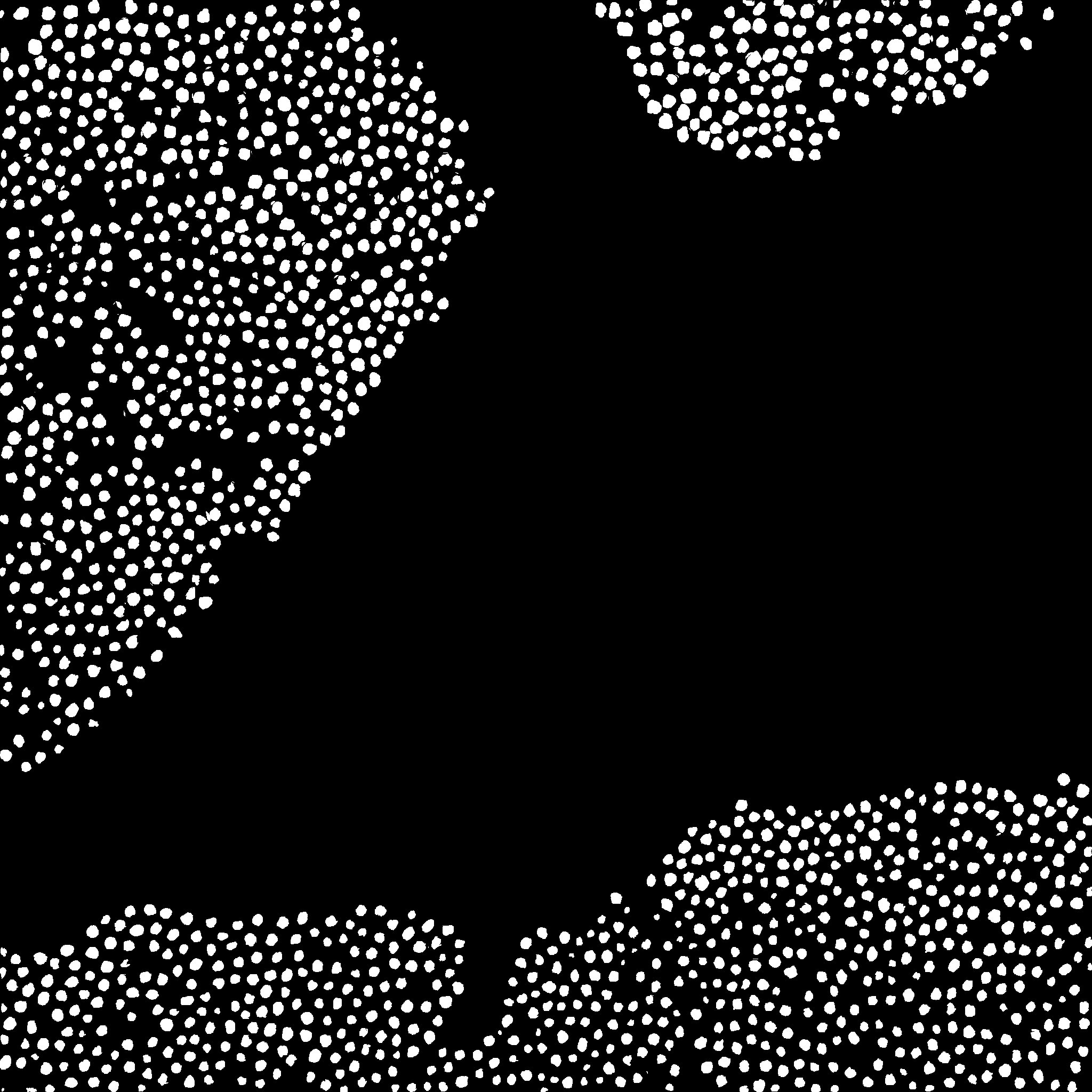

Supplement: S1 Dataset — (ZIP) [file pone.0312196.s002.zip › S2 Dataset/Ground Truth masks/2970 layered and median stacked_majority vote.jpg]

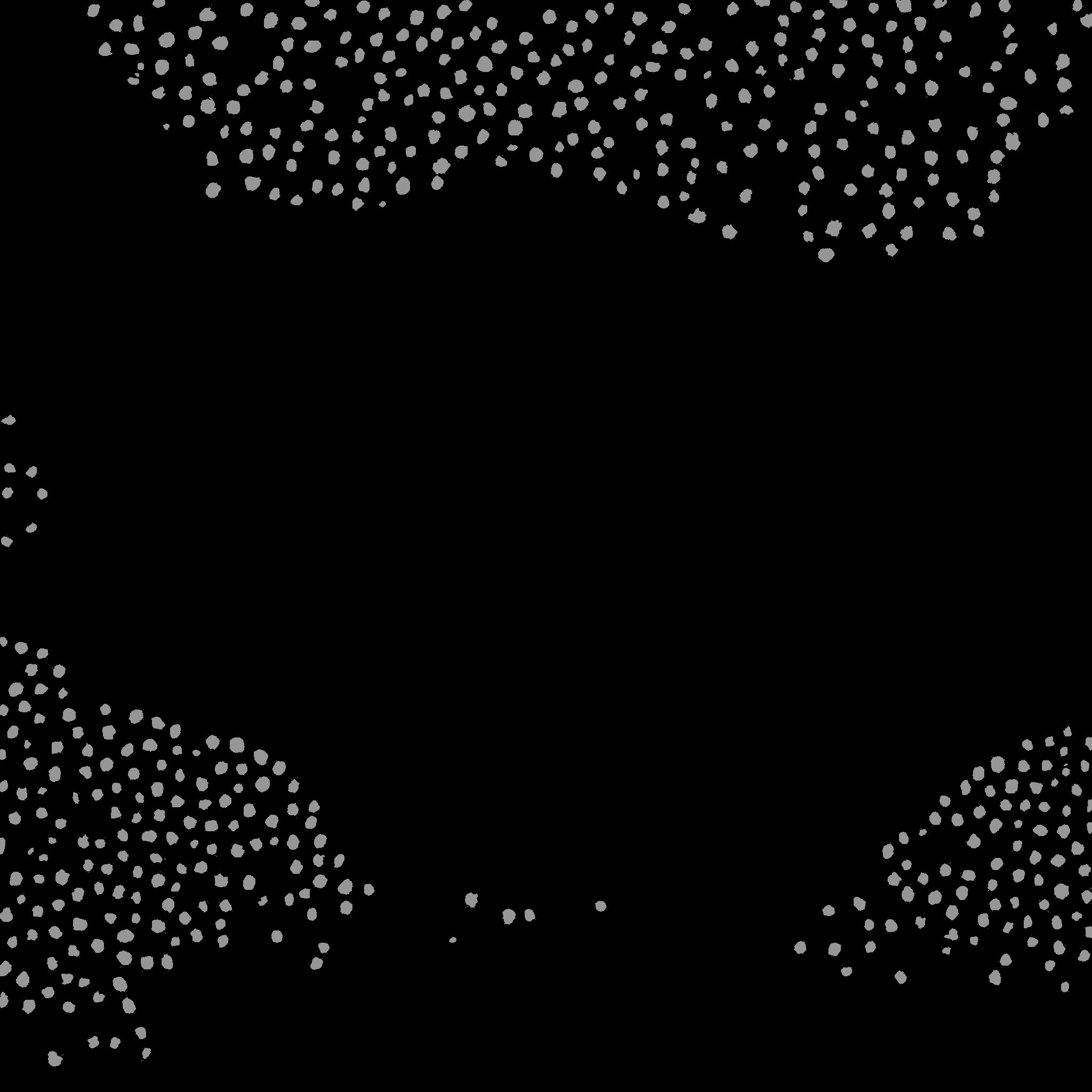

Supplement: S1 Dataset — (ZIP) [file pone.0312196.s002.zip › S2 Dataset/Ground Truth masks/2972 layered and median stacked_majority vote_mask from analysis.jpg]

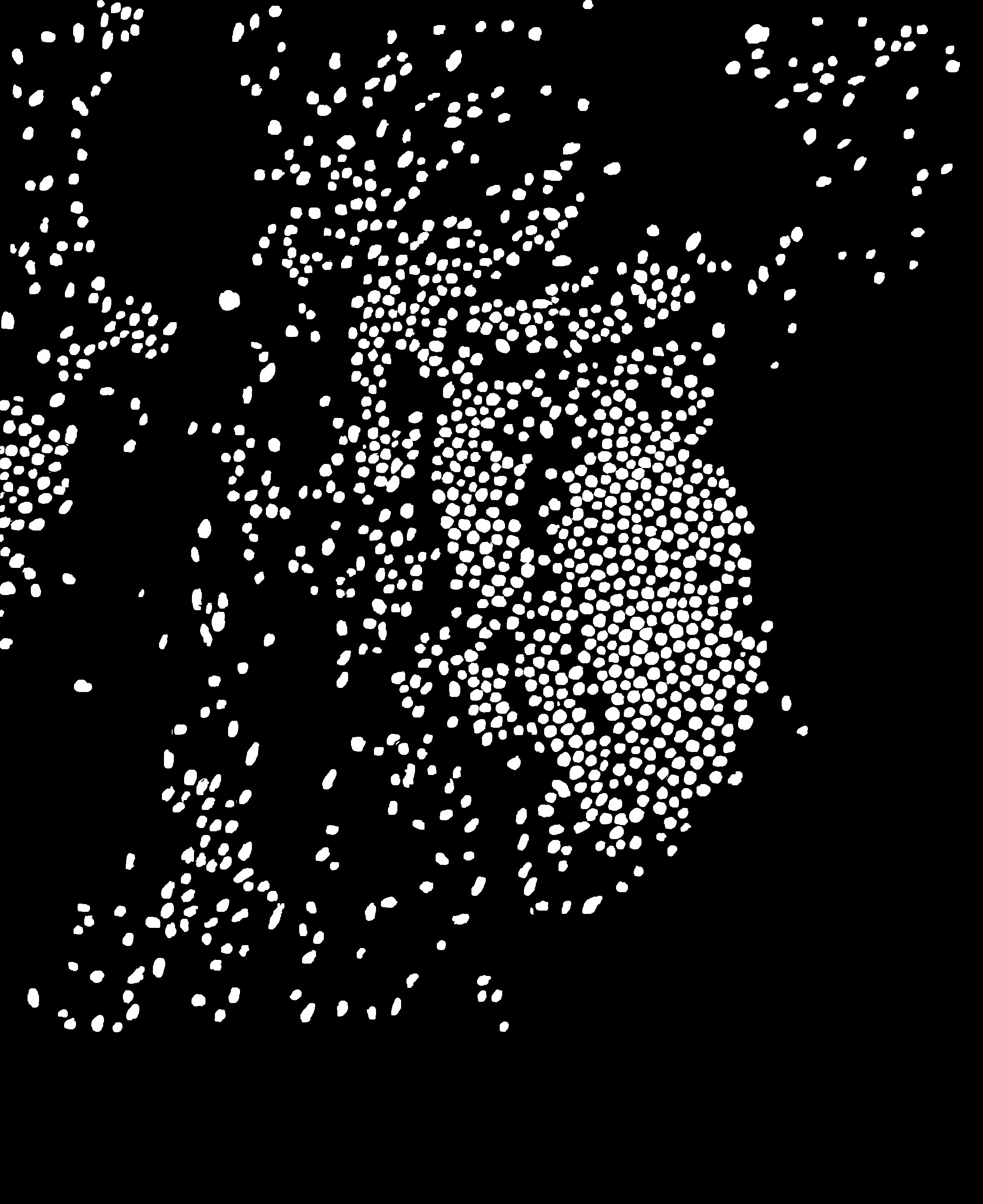

Supplement: S1 Dataset — (ZIP) [file pone.0312196.s002.zip › S2 Dataset/Ground Truth masks/GT NTG_E8_6_3_2024_003_16_fibrils.ome_2.jpg]

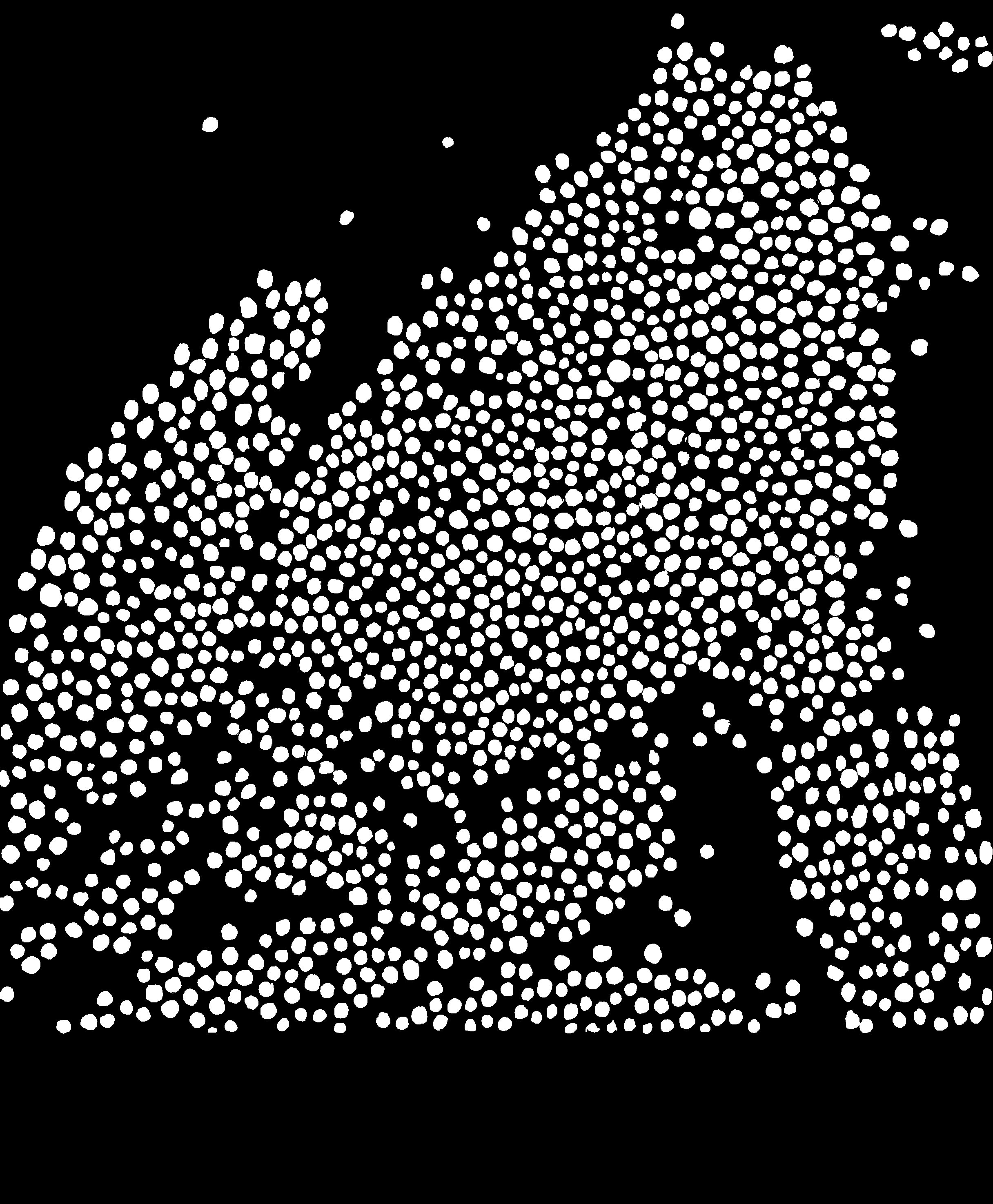

Supplement: S1 Dataset — (ZIP) [file pone.0312196.s002.zip › S2 Dataset/Ground Truth masks/GT S10_E6_21Feb24_012_16_fibrils.ome_3.jpg]

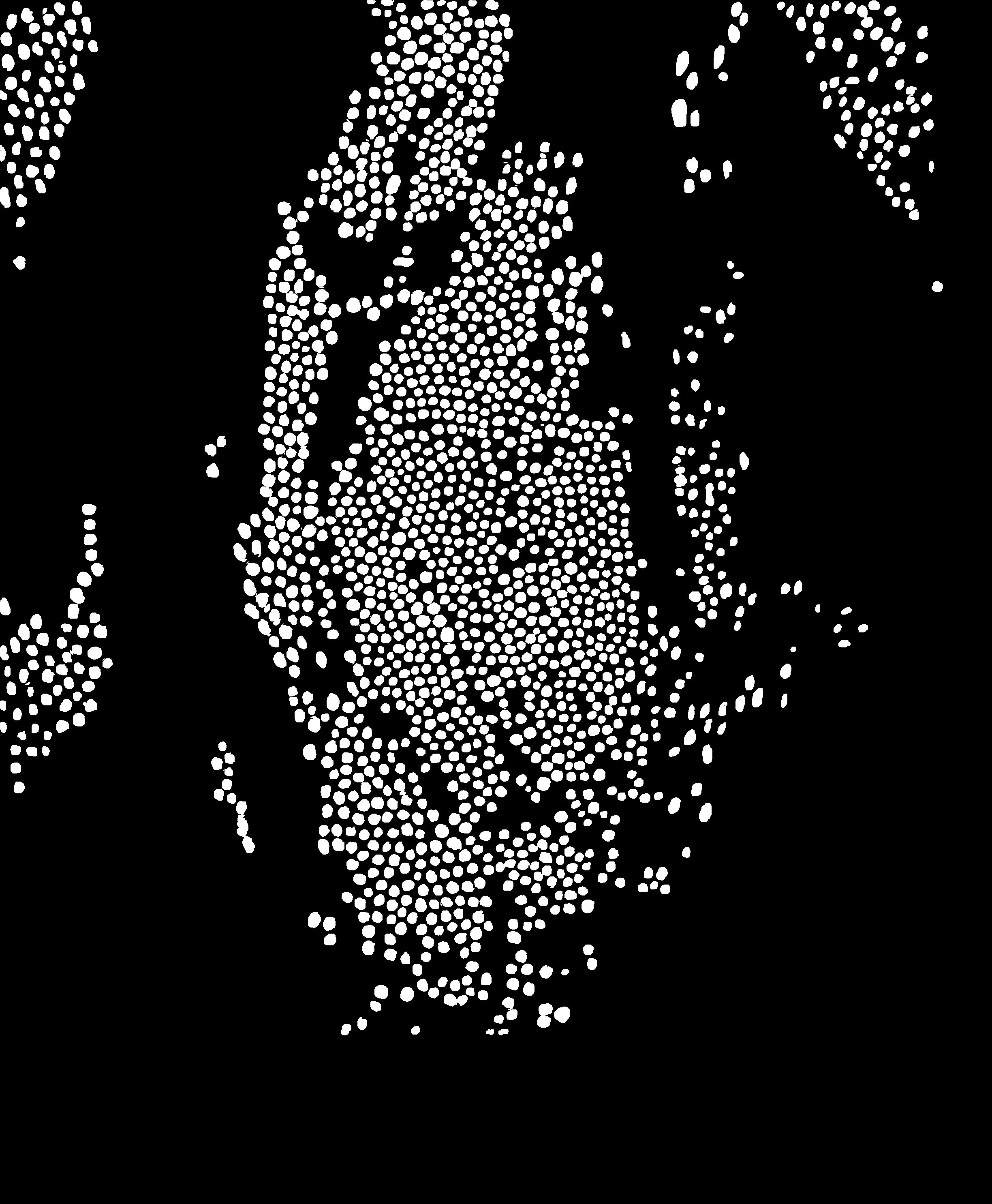

Supplement: S1 Dataset — (ZIP) [file pone.0312196.s002.zip › S2 Dataset/Ground Truth masks/GT_ NTG_E8_3_Jun_3_2024_022_16_background.ome_2.jpg]

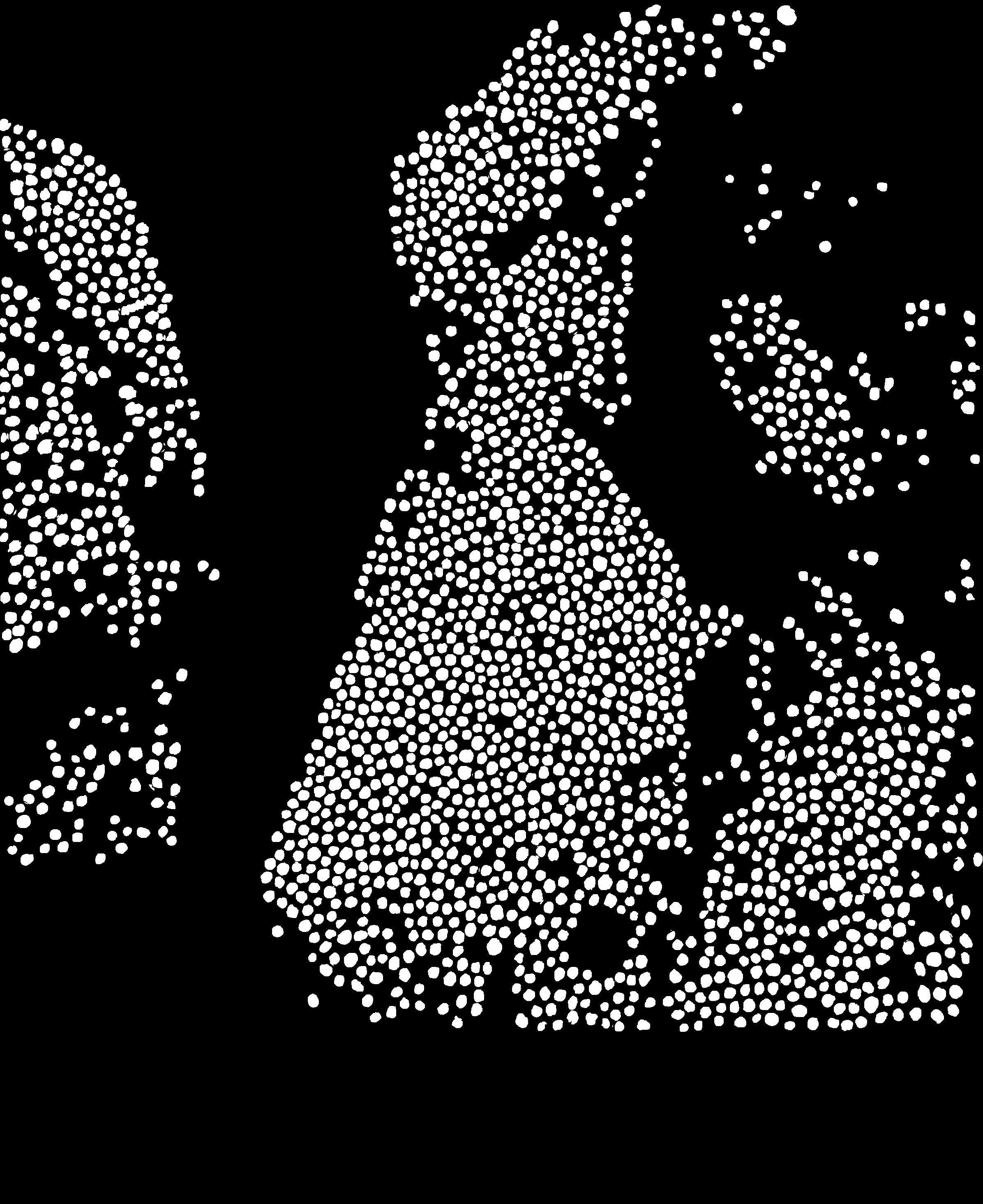

Supplement: S1 Dataset — (ZIP) [file pone.0312196.s002.zip › S2 Dataset/Ground Truth masks/GT_NTG_E8_2_Jun_3_2024_004_16_Fibrils.ome_2.jpg]

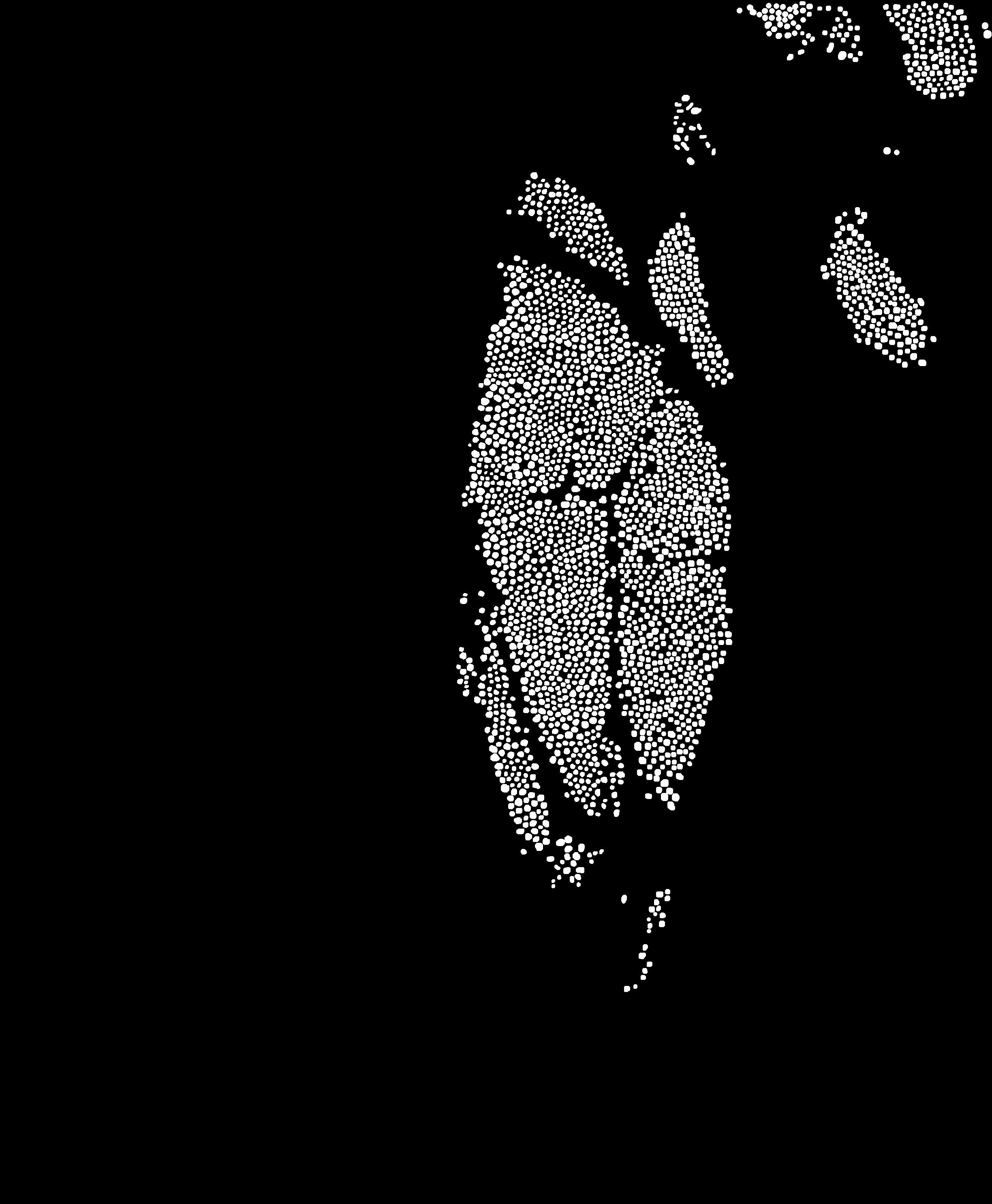

Supplement: S1 Dataset — (ZIP) [file pone.0312196.s002.zip › S2 Dataset/Ground Truth masks/GT_NTG_E8_3_Jun_3_2024_014_16_fibrils.jpg]

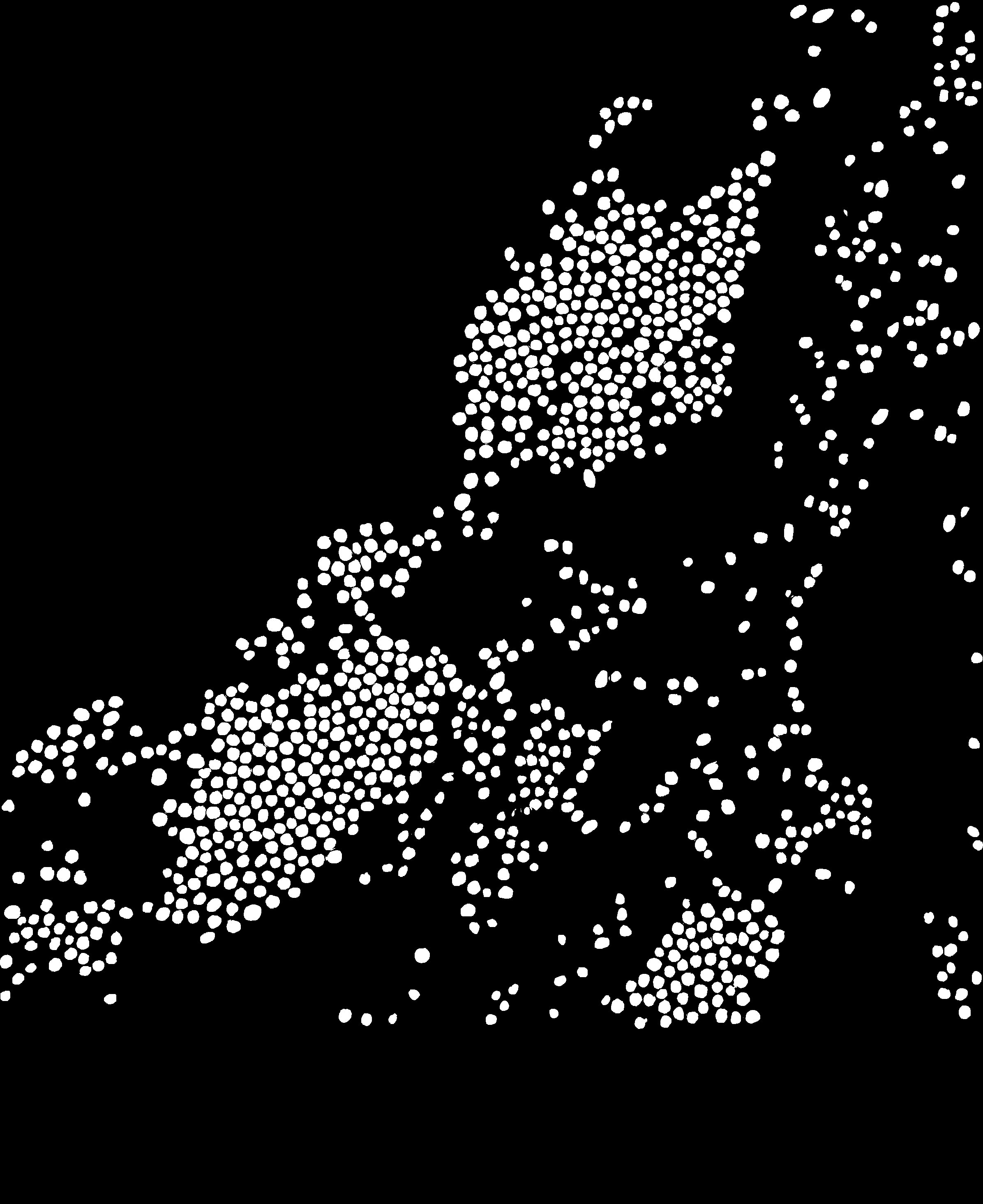

Supplement: S1 Dataset — (ZIP) [file pone.0312196.s002.zip › S2 Dataset/Ground Truth masks/GT_NTG_E8_6_3_2024_005_16_fibrils.ome_3.jpg]

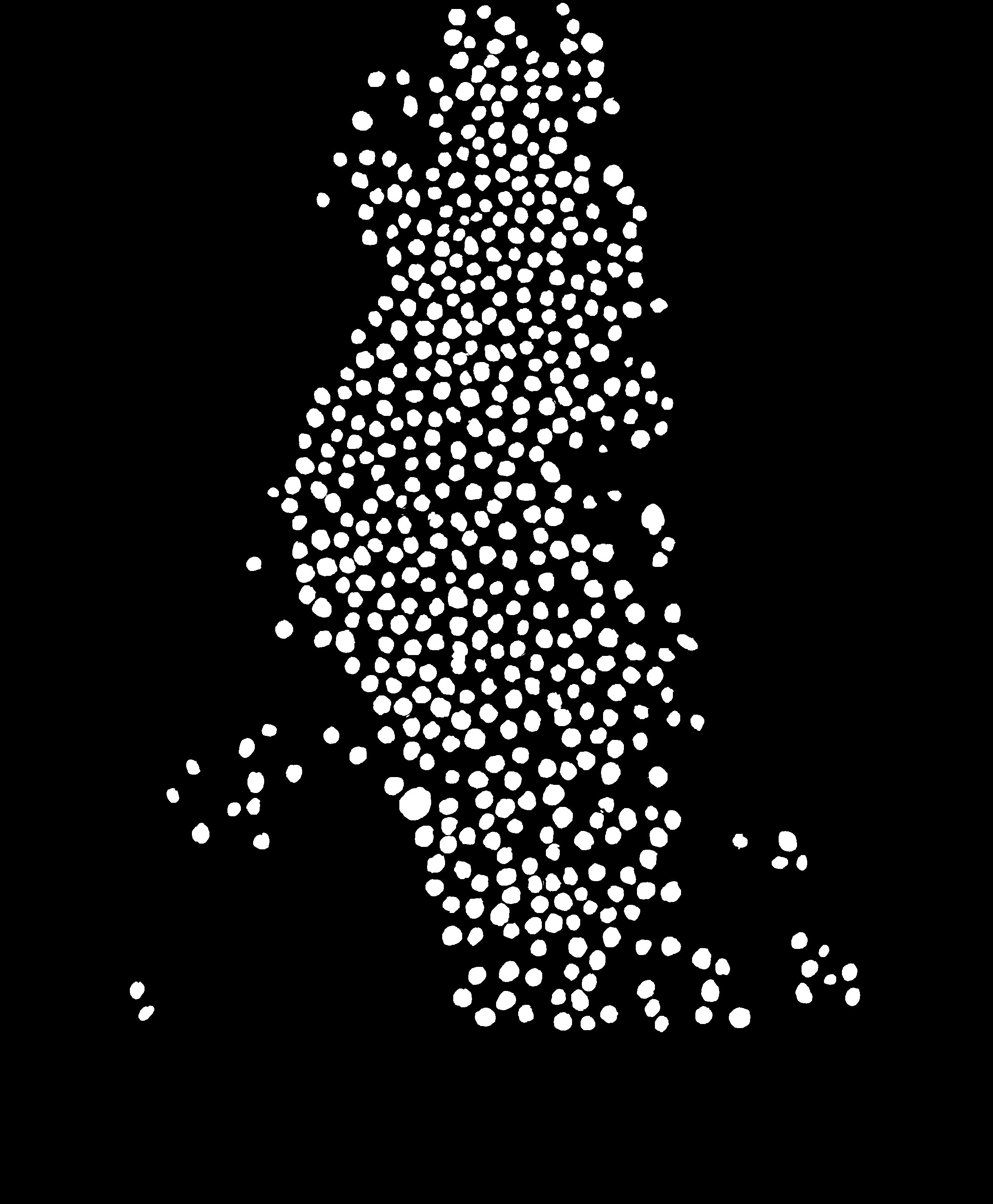

Supplement: S1 Dataset — (ZIP) [file pone.0312196.s002.zip › S2 Dataset/Ground Truth masks/GT_S2_B1_21Feb24_010_16_fibrils.ome_3.jpg]

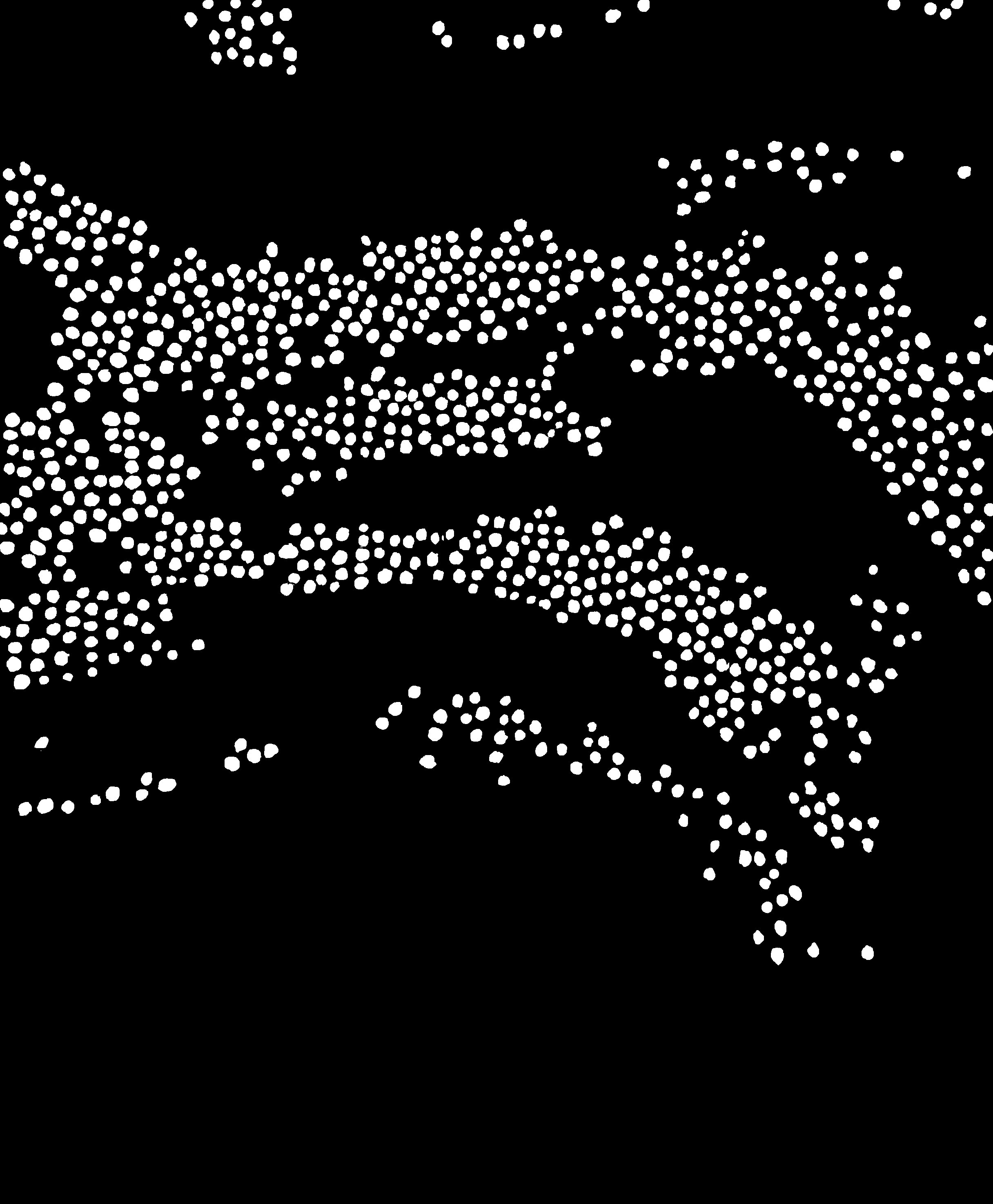

Supplement: S1 Dataset — (ZIP) [file pone.0312196.s002.zip › S2 Dataset/Ground Truth masks/GT_S3_C1_21Feb24_013_16_fibrils.ome_3.jpg]

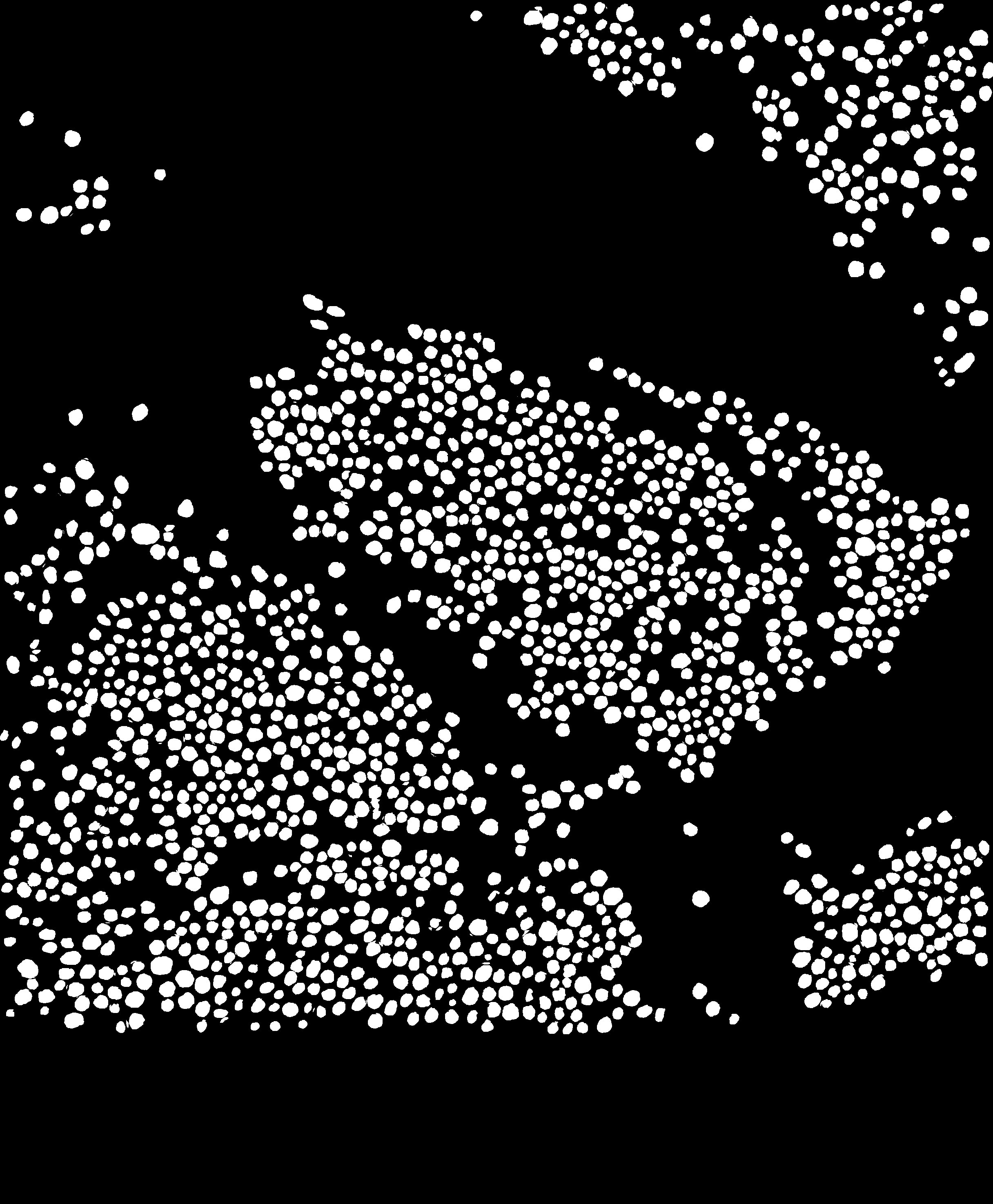

Supplement: S1 Dataset — (ZIP) [file pone.0312196.s002.zip › S2 Dataset/Ground Truth masks/GT_S5_E1_21Feb24_011_16_Fibrils.ome_2.jpg]

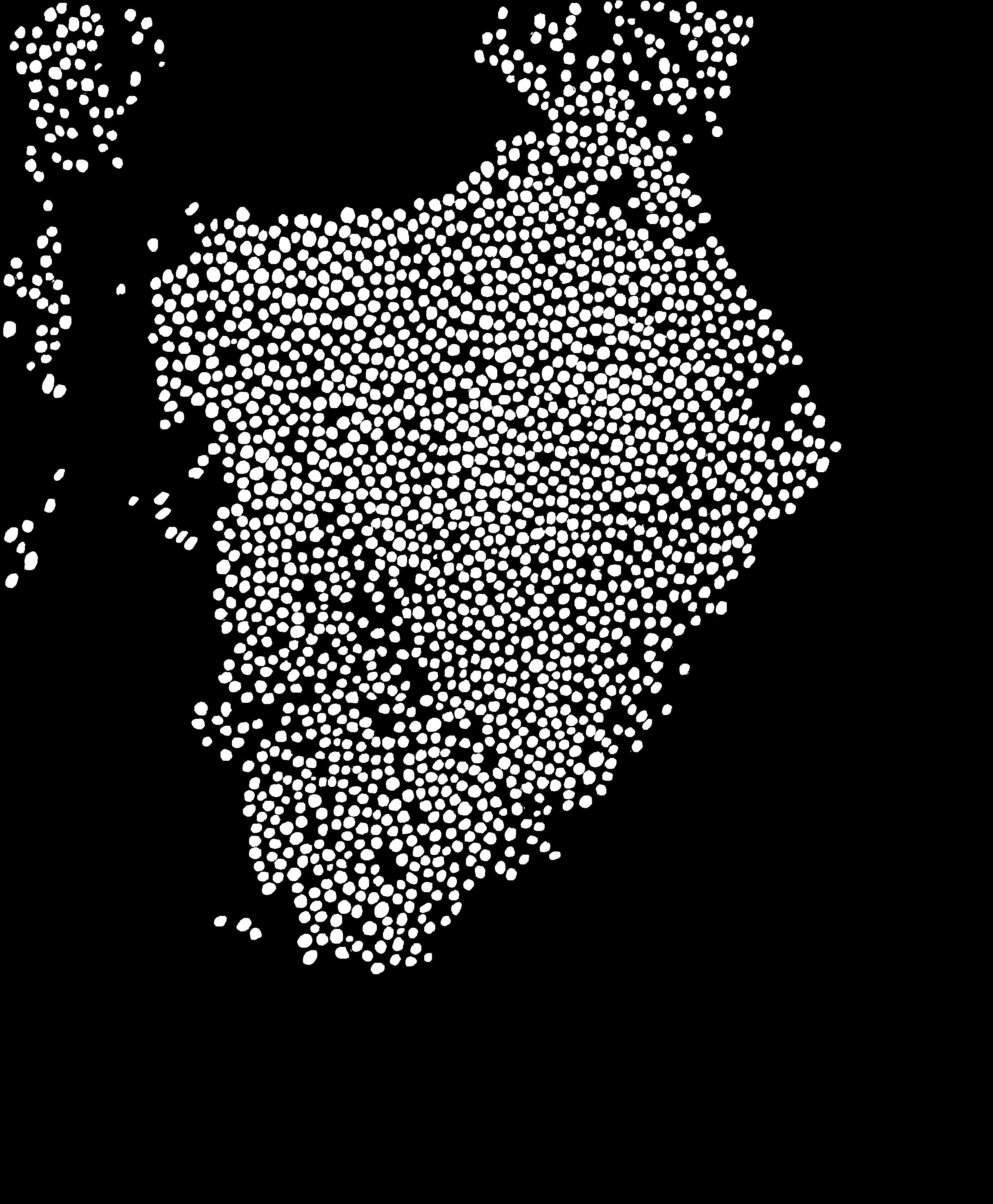

Supplement: S1 Dataset — (ZIP) [file pone.0312196.s002.zip › S2 Dataset/Ground Truth masks/GT_S6_A1_020224_011_16_fibrils.ome_3.jpg]

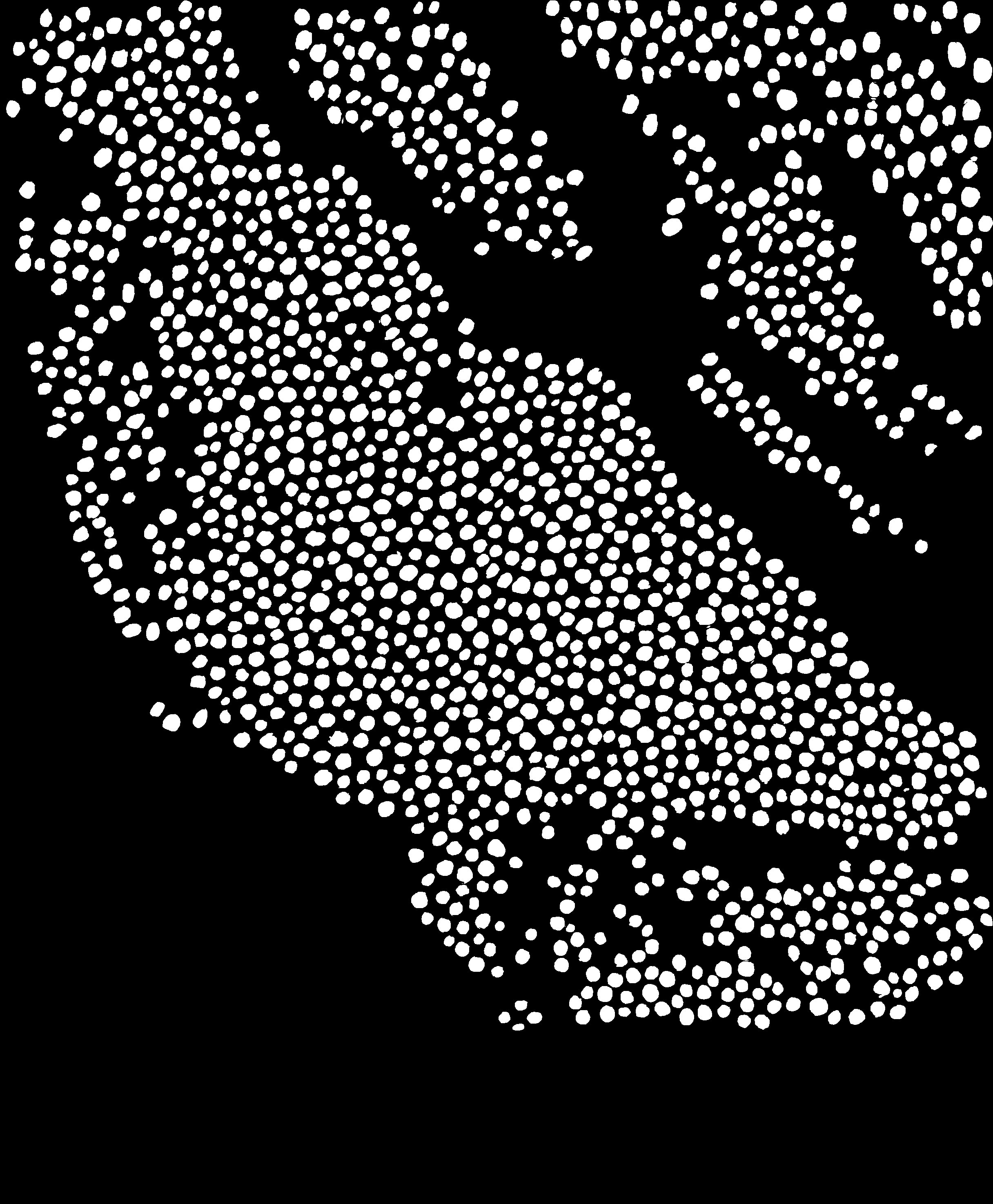

Supplement: S1 Dataset — (ZIP) [file pone.0312196.s002.zip › S2 Dataset/Ground Truth masks/GT_S8_C6_21Feb24_010_16_fibrils.ome_3.jpg]

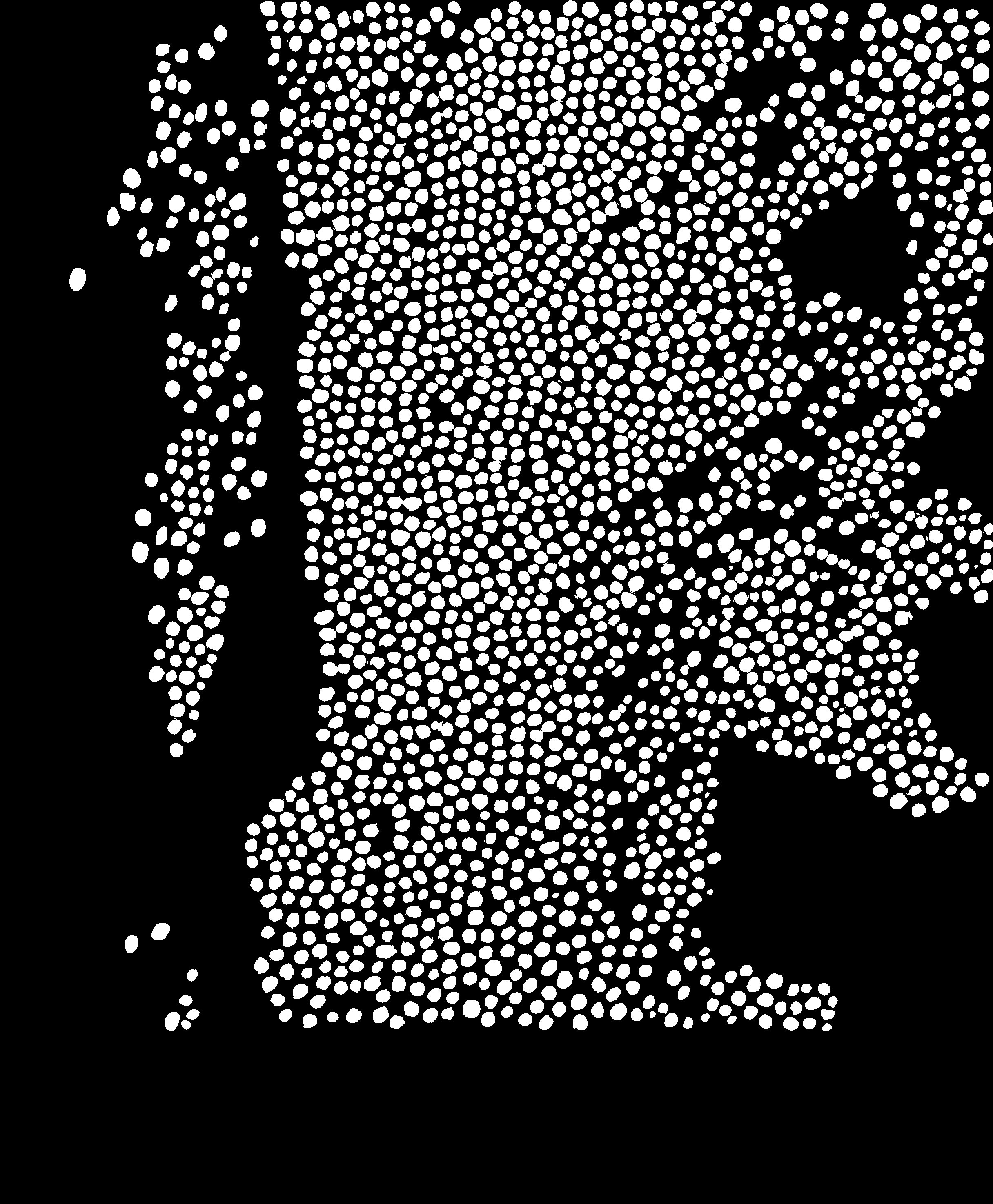

Supplement: S1 Dataset — (ZIP) [file pone.0312196.s002.zip › S2 Dataset/Ground Truth masks/GT_S9_D6_020224_007_16_Fibrils.ome_2.jpg]

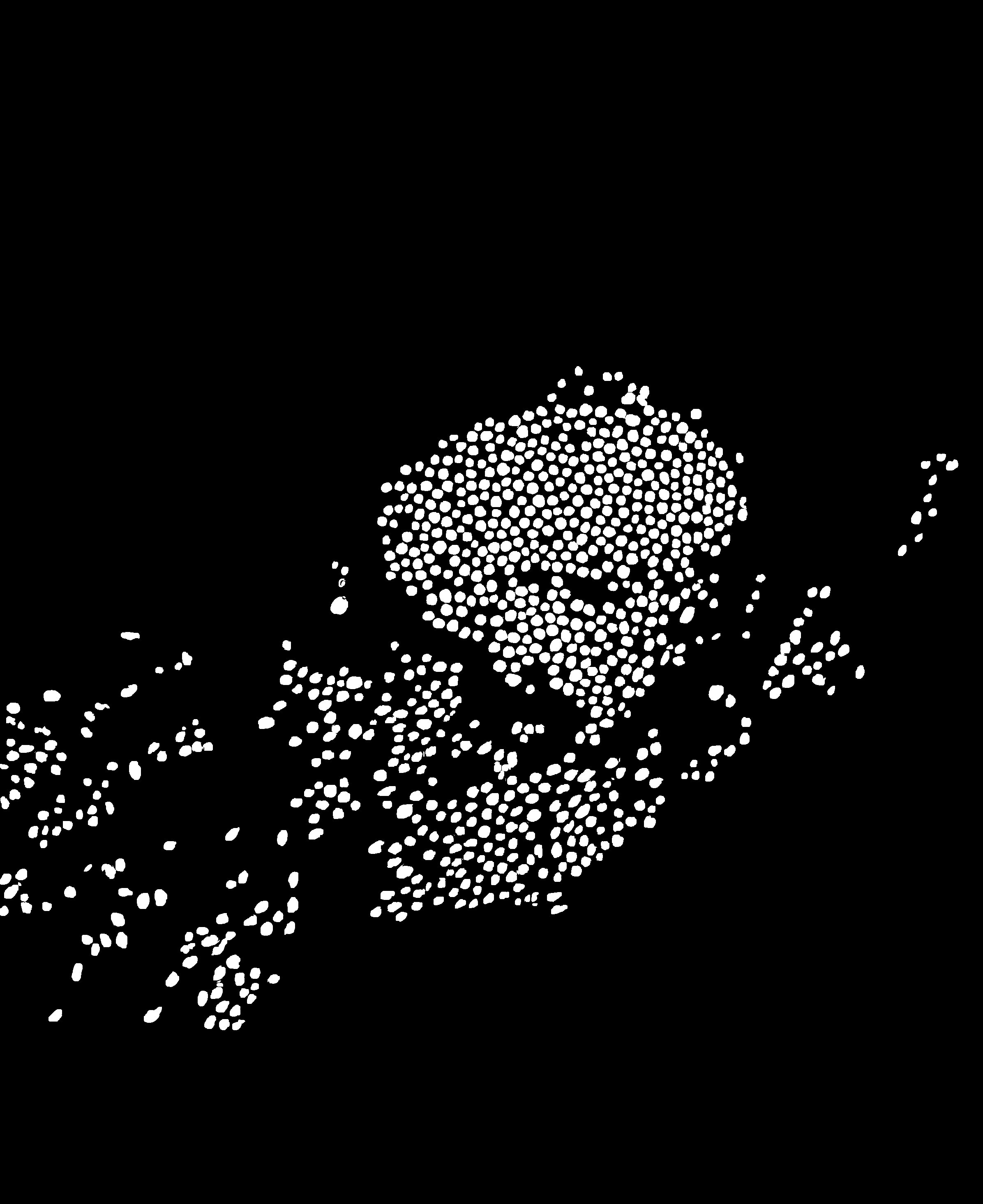

Supplement: S1 Dataset — (ZIP) [file pone.0312196.s002.zip › S2 Dataset/Ground Truth masks/Result of Result of Result of NTG_E8_6_3_2024_008_16_background.ome_1.jpg]

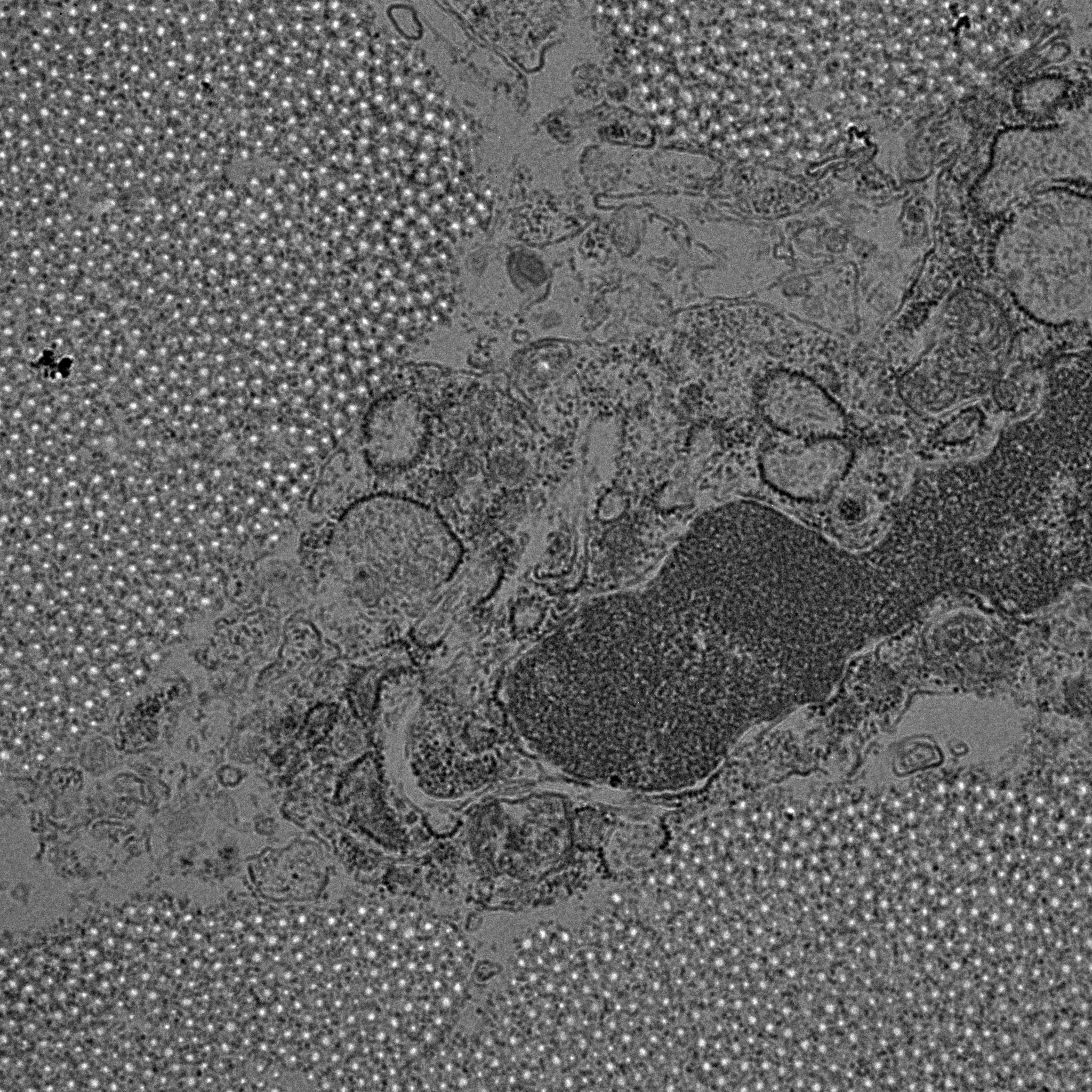

Supplement: S2 Dataset — (ZIP) [file pone.0312196.s003.zip › S3 Dataset/Testing Images/Image1.jpg]

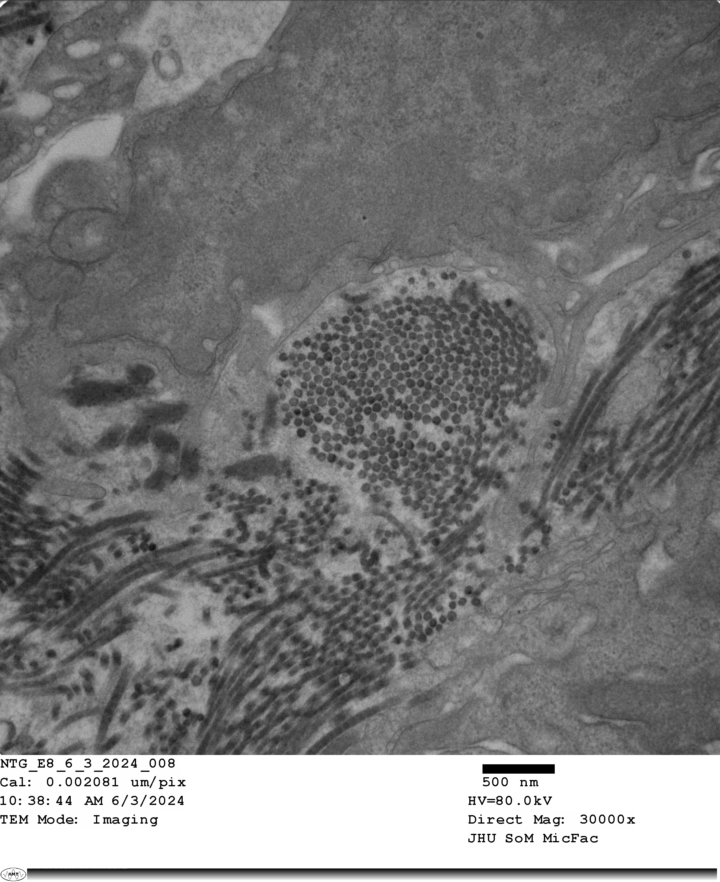

Supplement: S2 Dataset — (ZIP) [file pone.0312196.s003.zip › S3 Dataset/Testing Images/Image10.TIF]

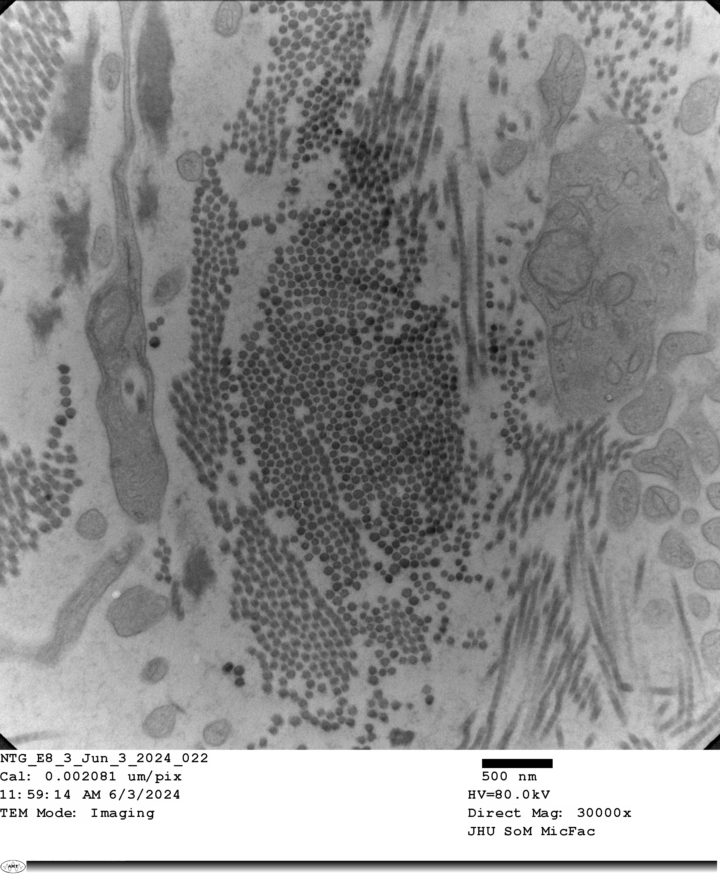

Supplement: S2 Dataset — (ZIP) [file pone.0312196.s003.zip › S3 Dataset/Testing Images/Image11.TIF]

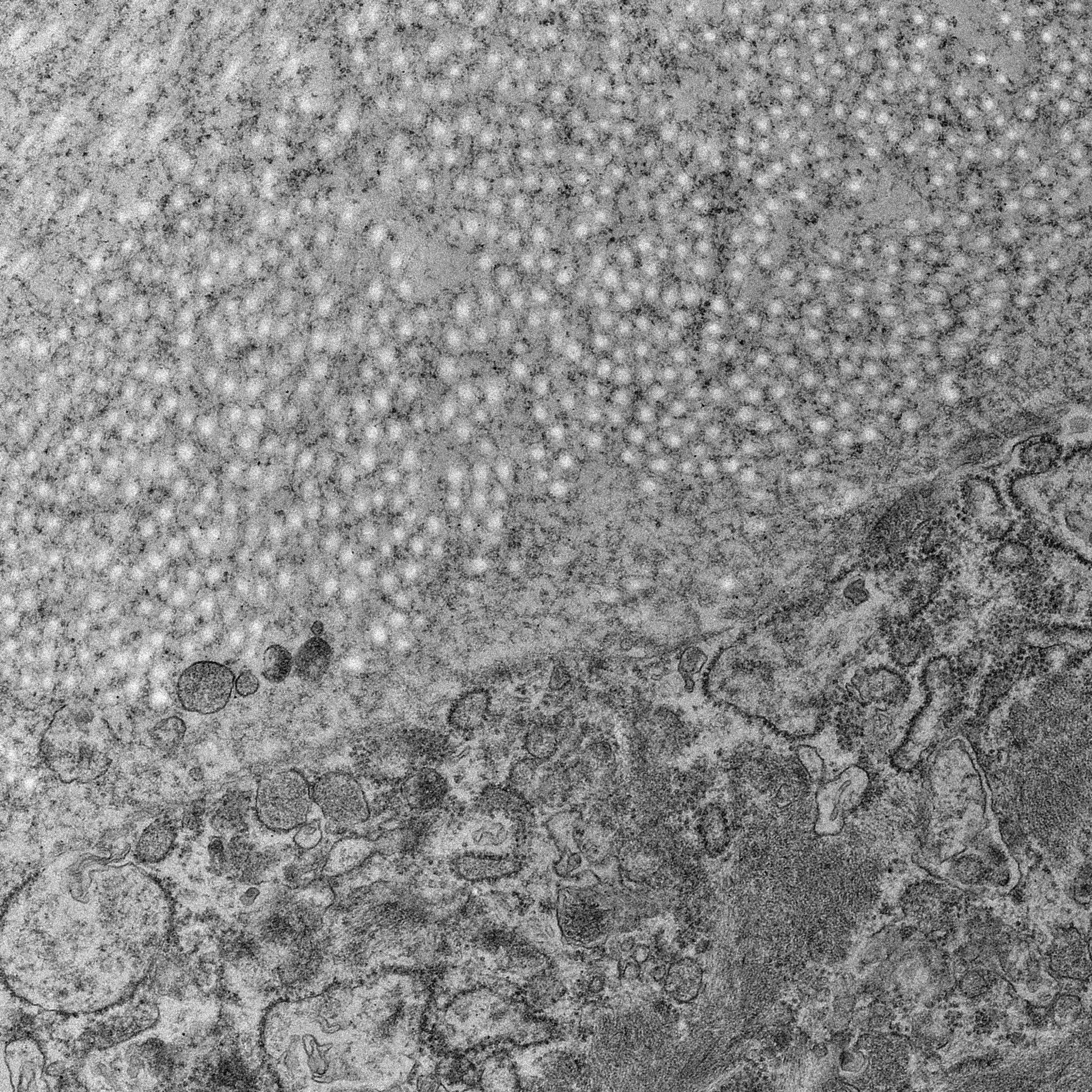

Supplement: S2 Dataset — (ZIP) [file pone.0312196.s003.zip › S3 Dataset/Testing Images/Image2.jpg]

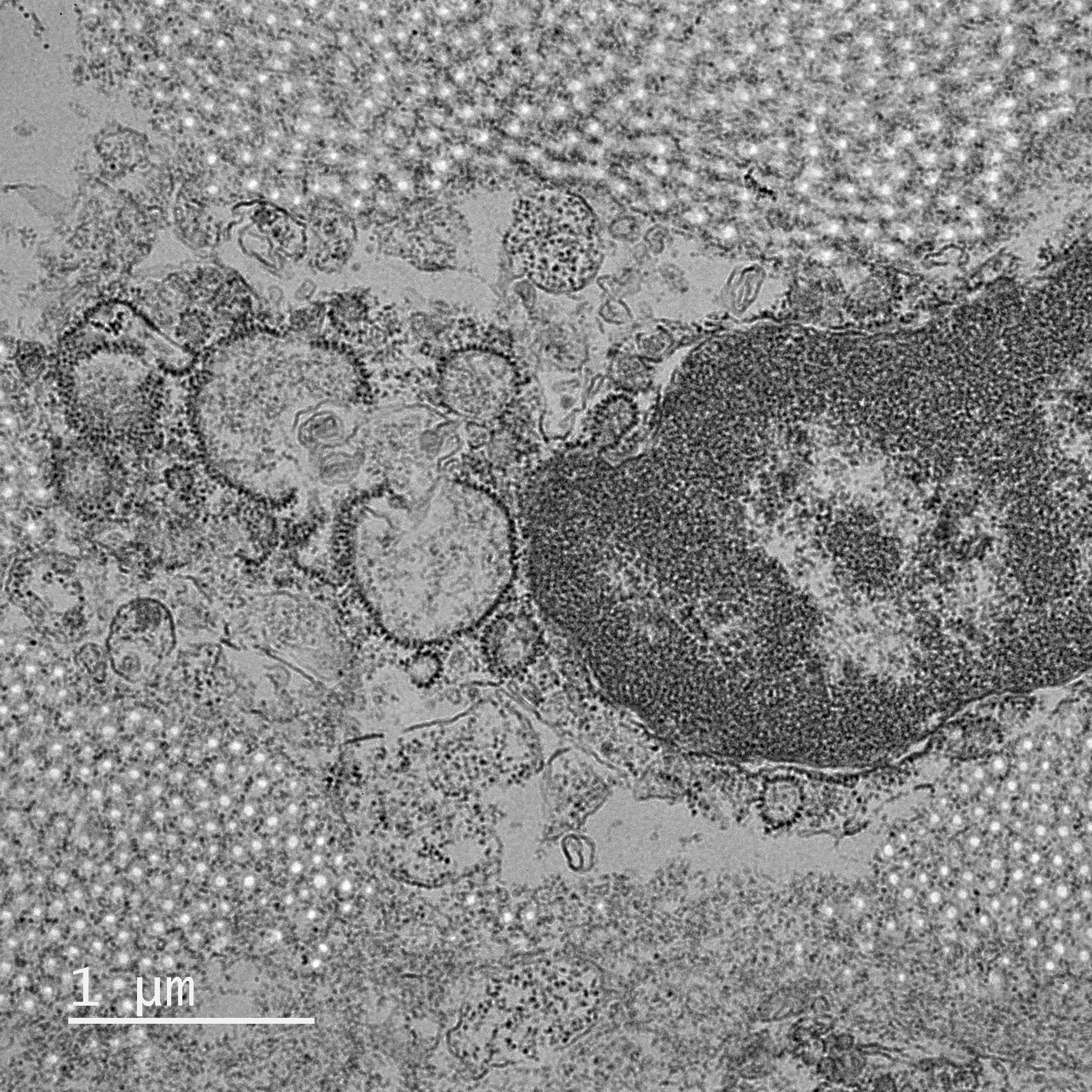

Supplement: S2 Dataset — (ZIP) [file pone.0312196.s003.zip › S3 Dataset/Testing Images/Image3.jpg]

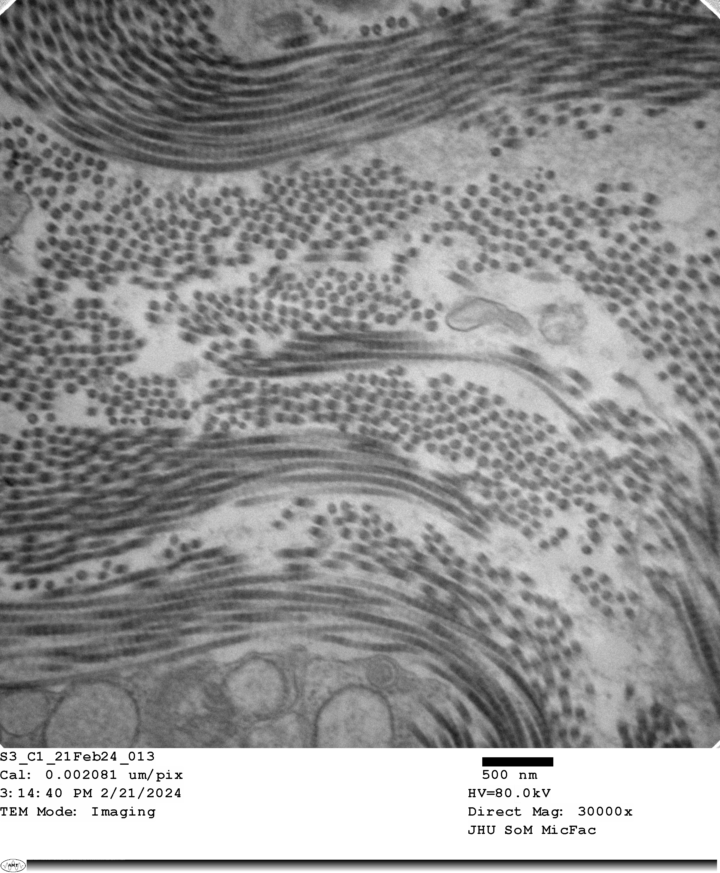

Supplement: S2 Dataset — (ZIP) [file pone.0312196.s003.zip › S3 Dataset/Testing Images/Image4.TIF]

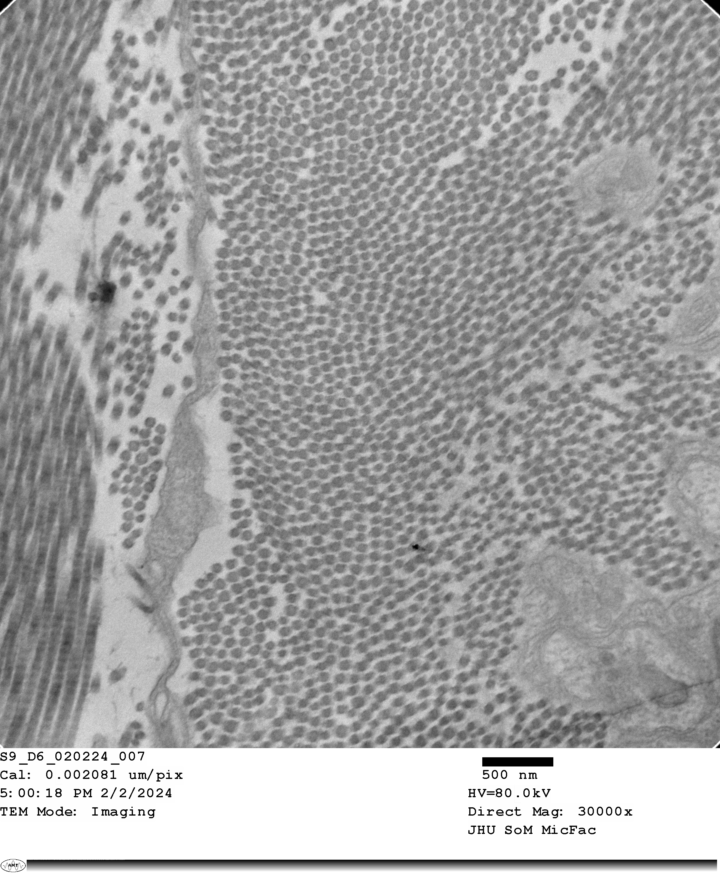

Supplement: S2 Dataset — (ZIP) [file pone.0312196.s003.zip › S3 Dataset/Testing Images/Image5.TIF]

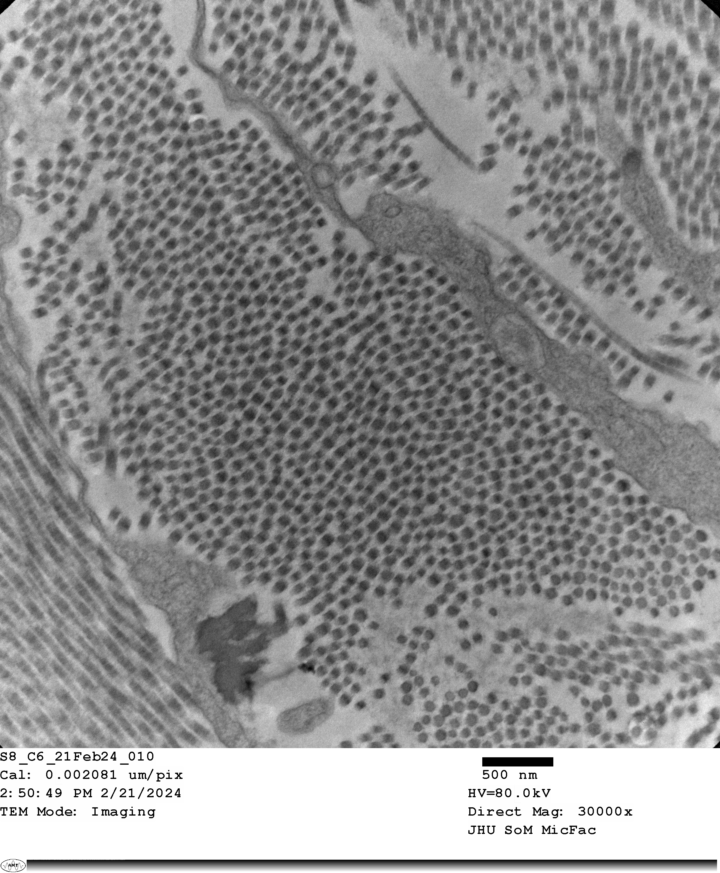

Supplement: S2 Dataset — (ZIP) [file pone.0312196.s003.zip › S3 Dataset/Testing Images/Image6.TIF]

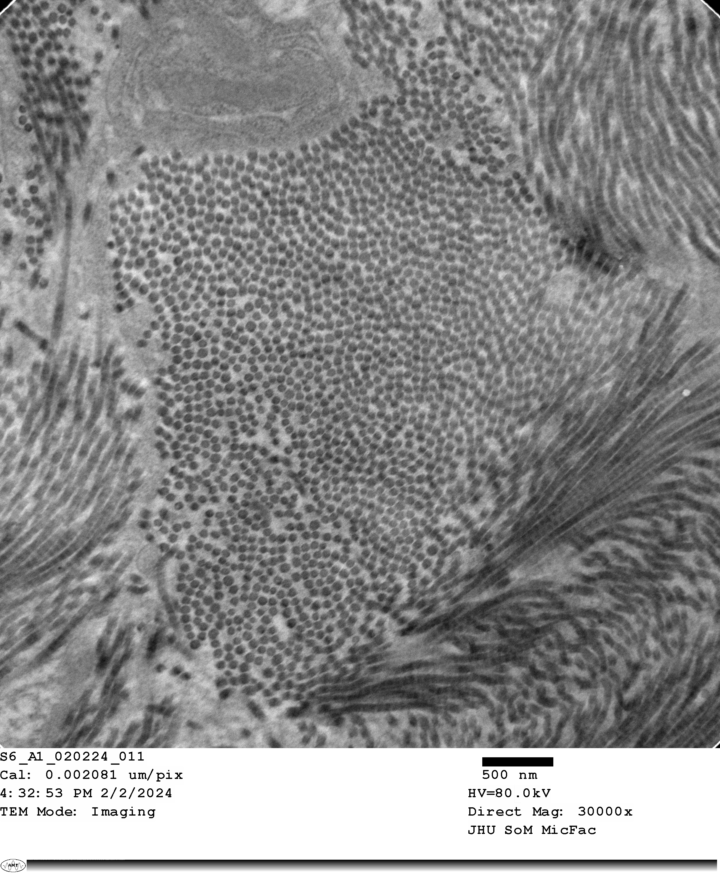

Supplement: S2 Dataset — (ZIP) [file pone.0312196.s003.zip › S3 Dataset/Testing Images/Image7.TIF]

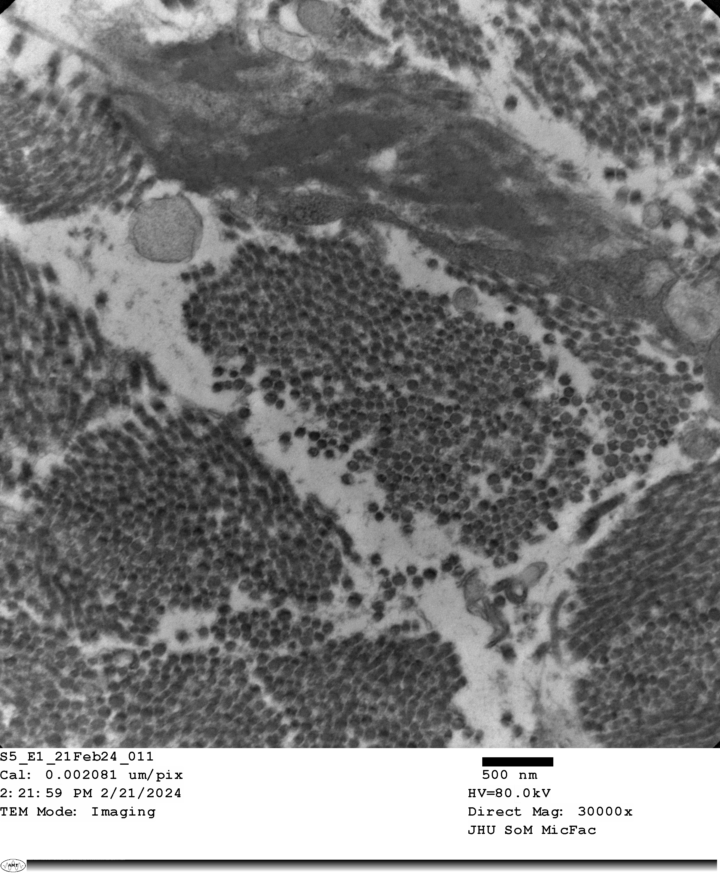

Supplement: S2 Dataset — (ZIP) [file pone.0312196.s003.zip › S3 Dataset/Testing Images/Image8.TIF]

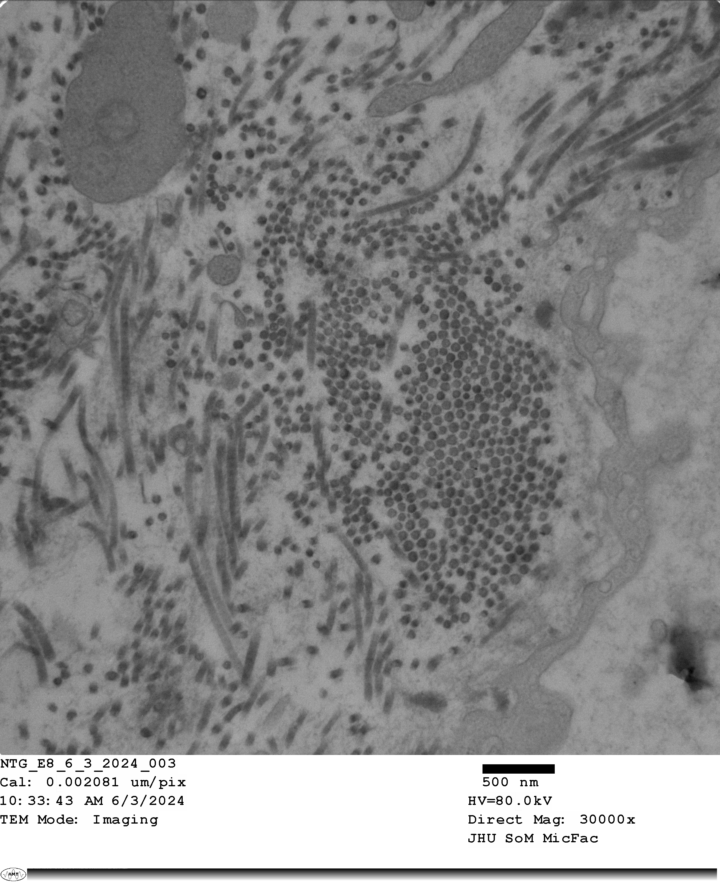

Supplement: S2 Dataset — (ZIP) [file pone.0312196.s003.zip › S3 Dataset/Testing Images/Image9.TIF]

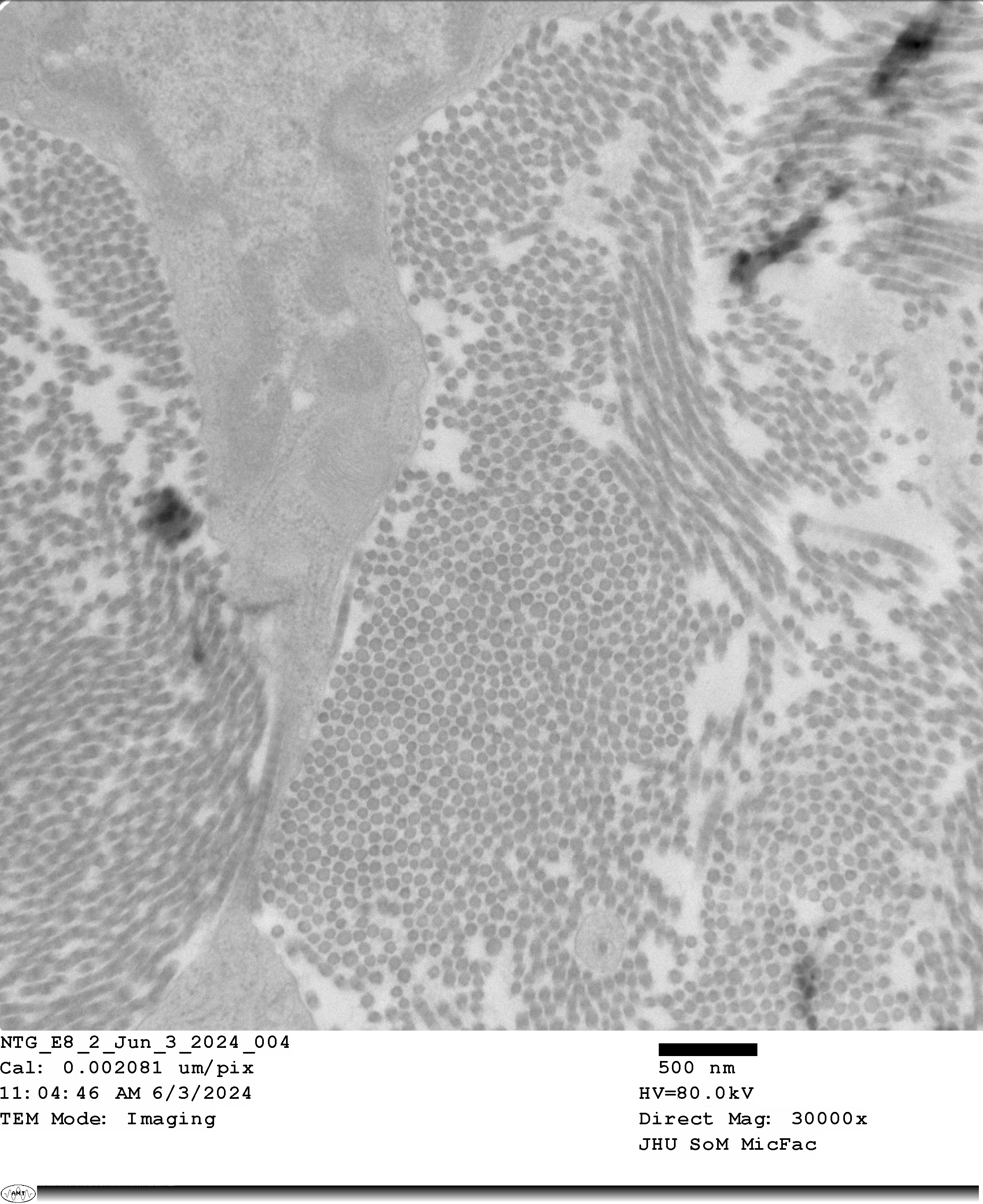

Supplement: S2 Dataset — (ZIP) [file pone.0312196.s003.zip › S3 Dataset/Training Images/NTG_E8_2_Jun_3_2024_004_16.TIF]

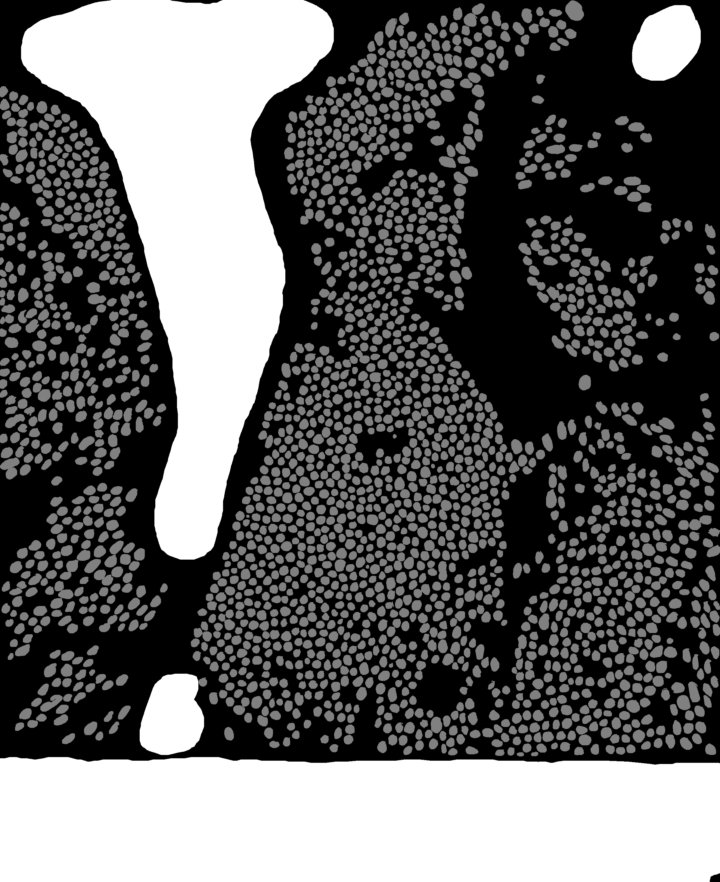

Supplement: S2 Dataset — (ZIP) [file pone.0312196.s003.zip › S3 Dataset/Training Images/NTG_E8_2_Jun_3_2024_004_16_background.ome.jpg]

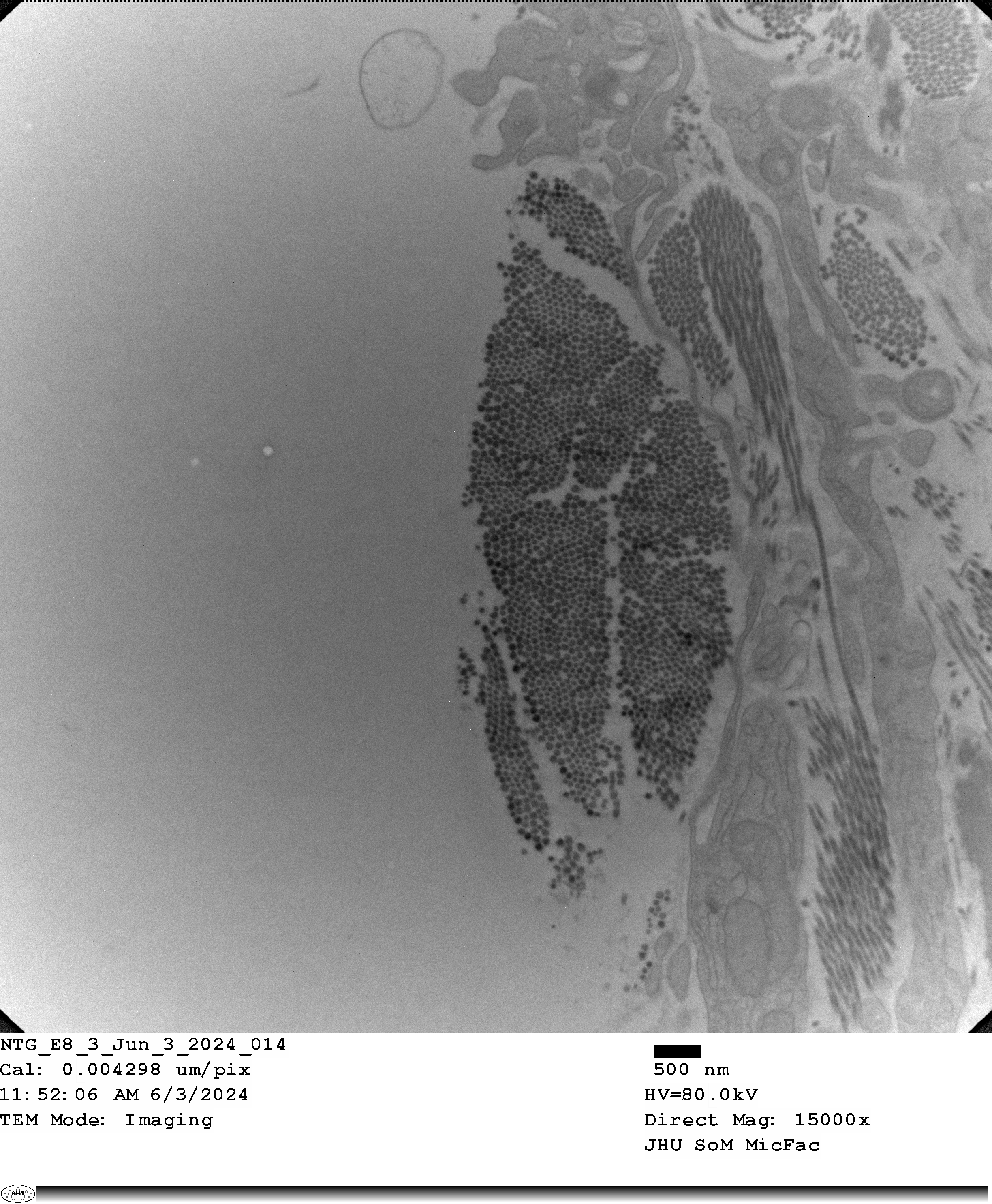

Supplement: S2 Dataset — (ZIP) [file pone.0312196.s003.zip › S3 Dataset/Training Images/NTG_E8_3_Jun_3_2024_014_16.TIF]

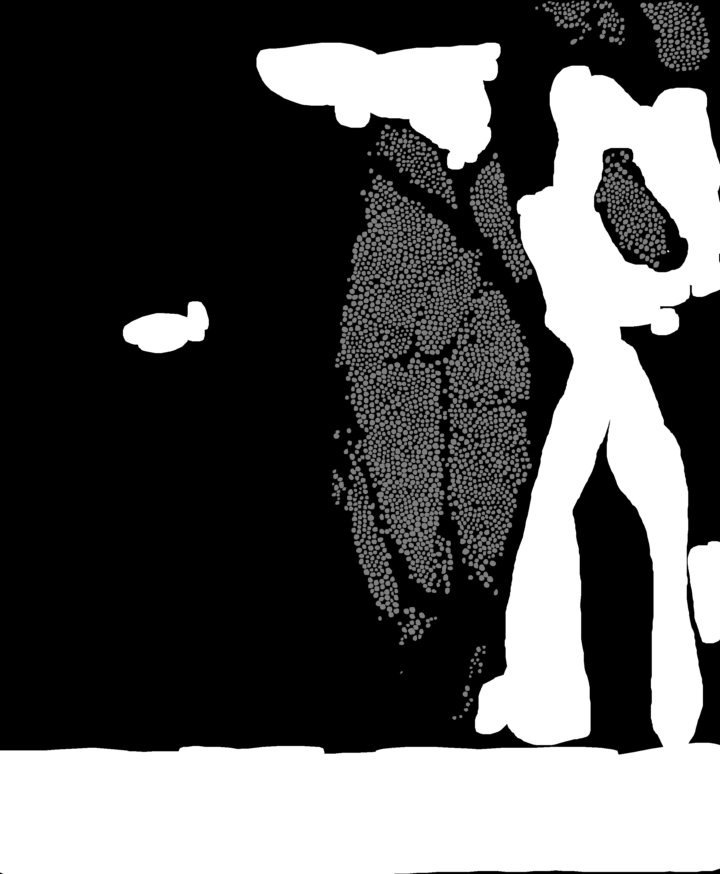

Supplement: S2 Dataset — (ZIP) [file pone.0312196.s003.zip › S3 Dataset/Training Images/NTG_E8_3_Jun_3_2024_014_16_Fibrils.ome.jpg]

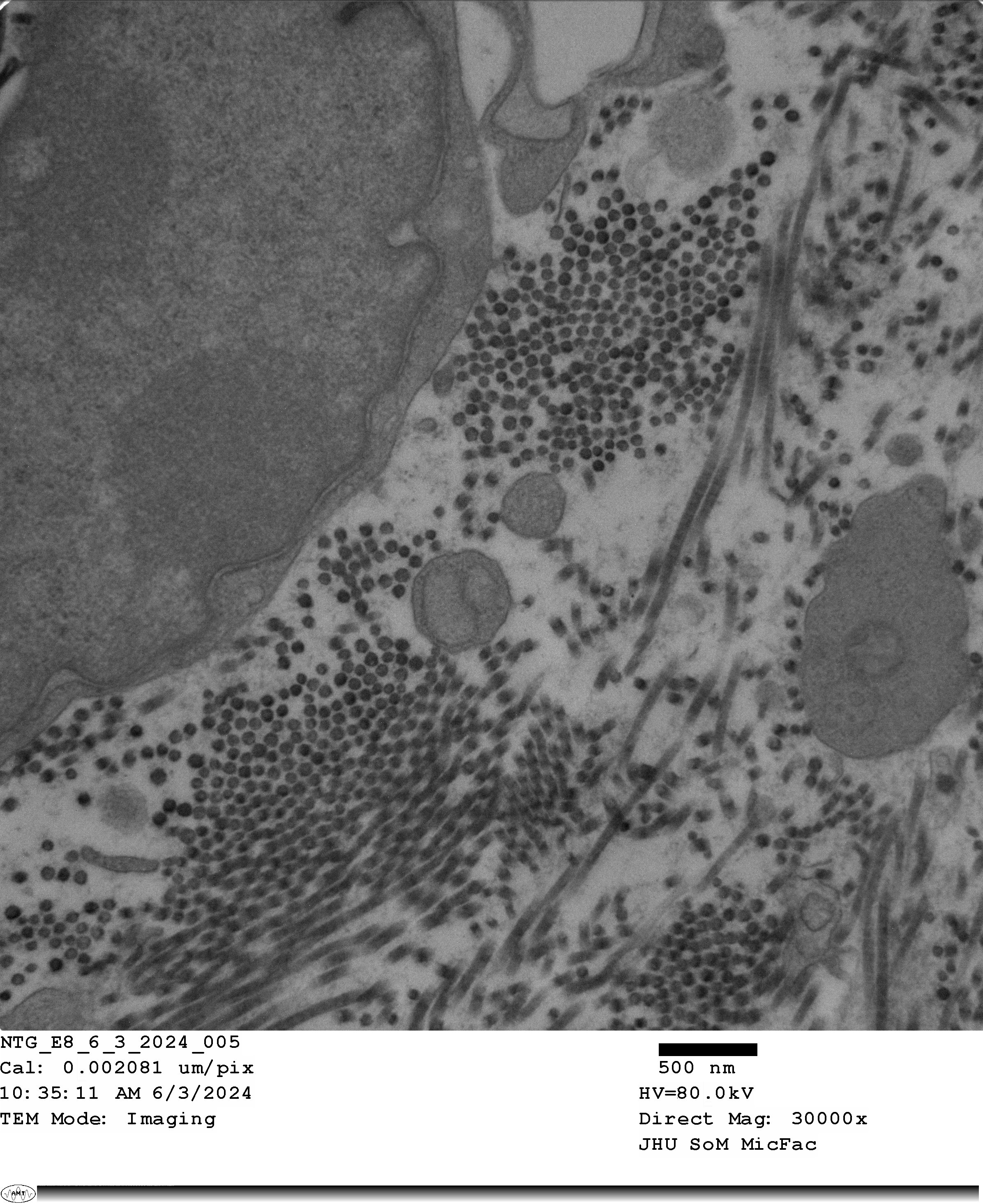

Supplement: S2 Dataset — (ZIP) [file pone.0312196.s003.zip › S3 Dataset/Training Images/NTG_E8_6_3_2024_005_16.TIF]

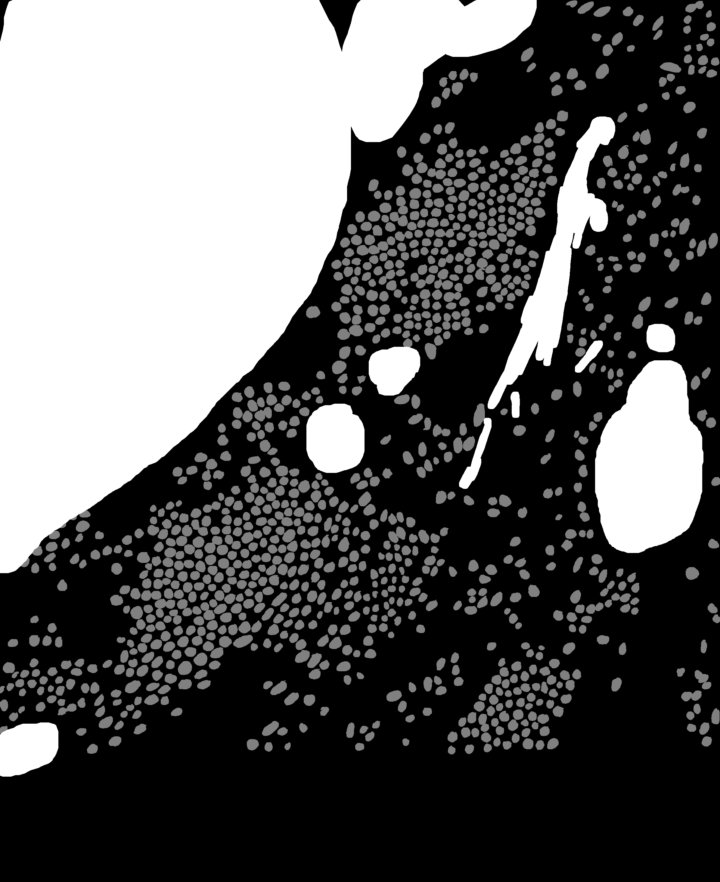

Supplement: S2 Dataset — (ZIP) [file pone.0312196.s003.zip › S3 Dataset/Training Images/NTG_E8_6_3_2024_005_16_background.ome.jpg]

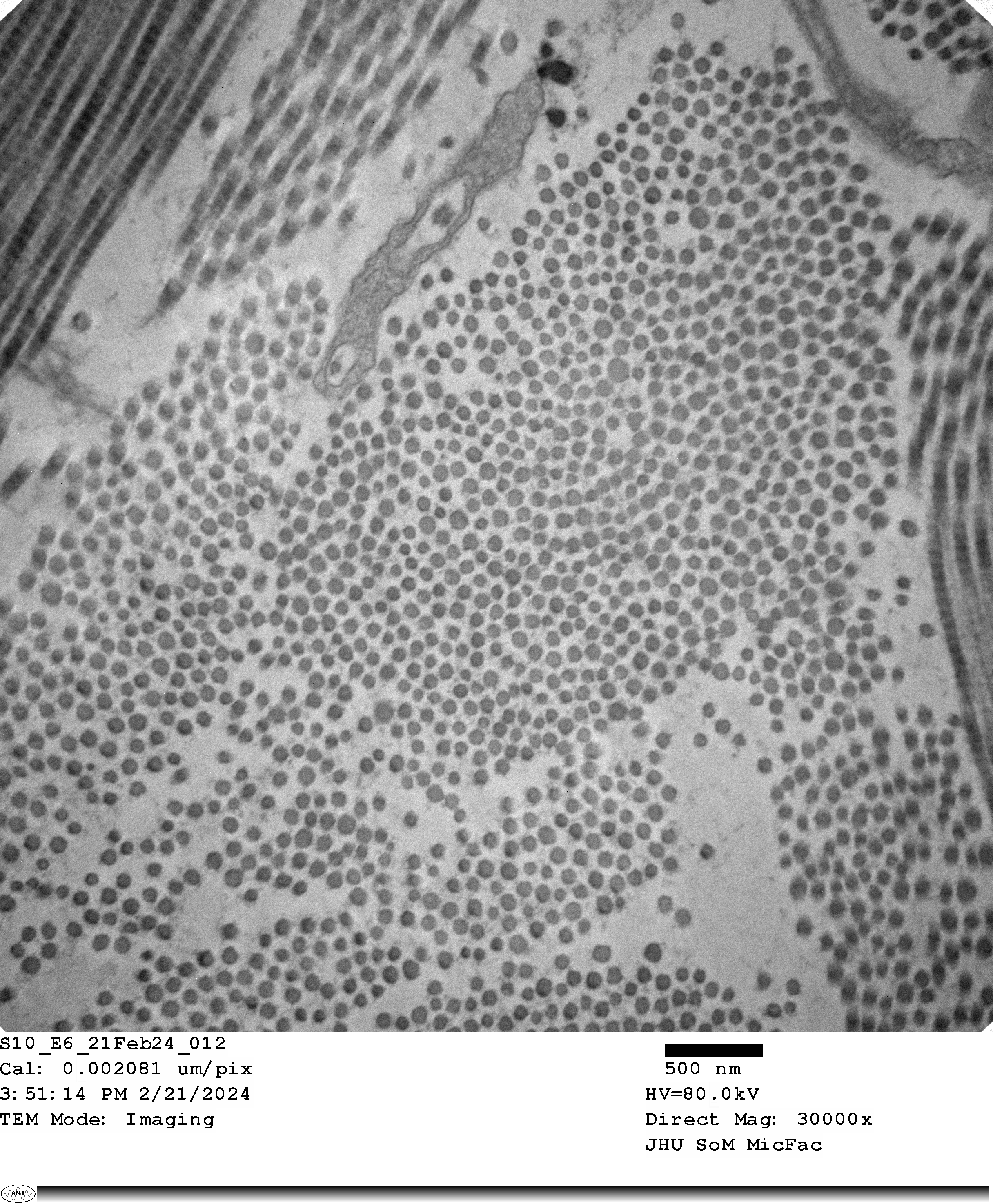

Supplement: S2 Dataset — (ZIP) [file pone.0312196.s003.zip › S3 Dataset/Training Images/S10_E6_21Feb24_012_16.TIF]

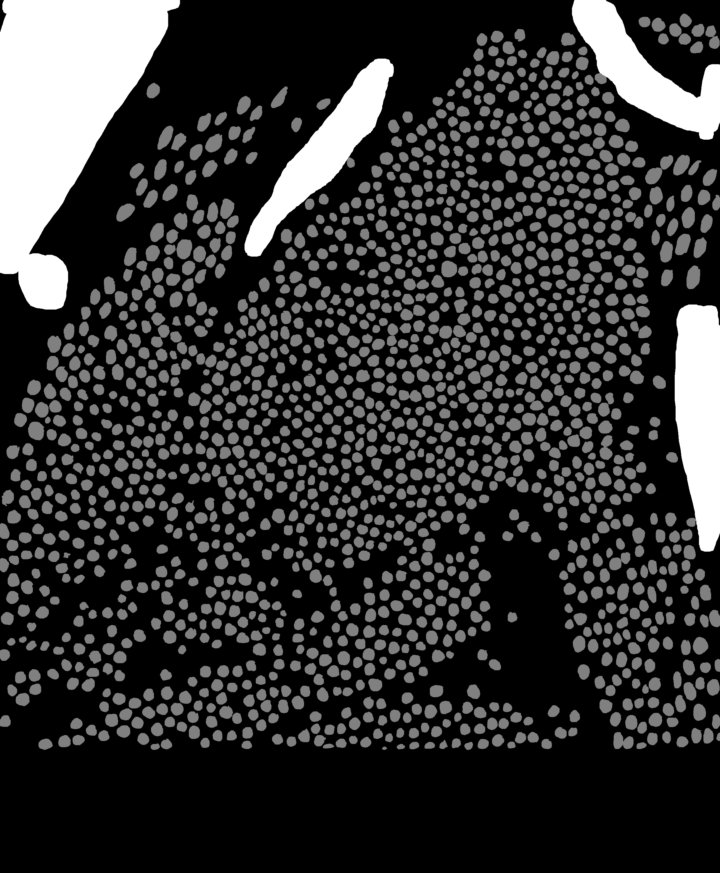

Supplement: S2 Dataset — (ZIP) [file pone.0312196.s003.zip › S3 Dataset/Training Images/S10_E6_21Feb24_012_16_background.ome.jpg]

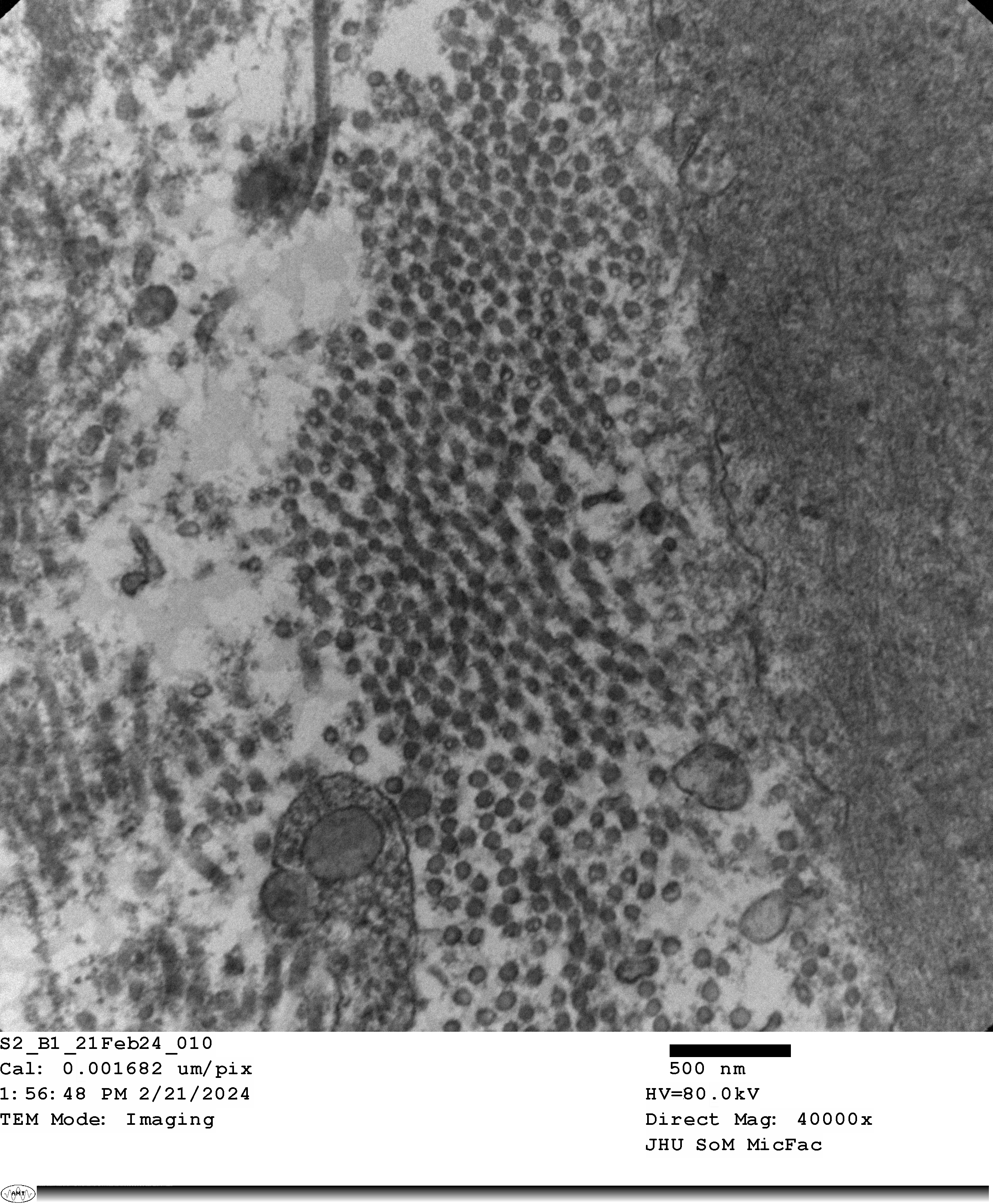

Supplement: S2 Dataset — (ZIP) [file pone.0312196.s003.zip › S3 Dataset/Training Images/S2_B1_21Feb24_010_16.TIF]

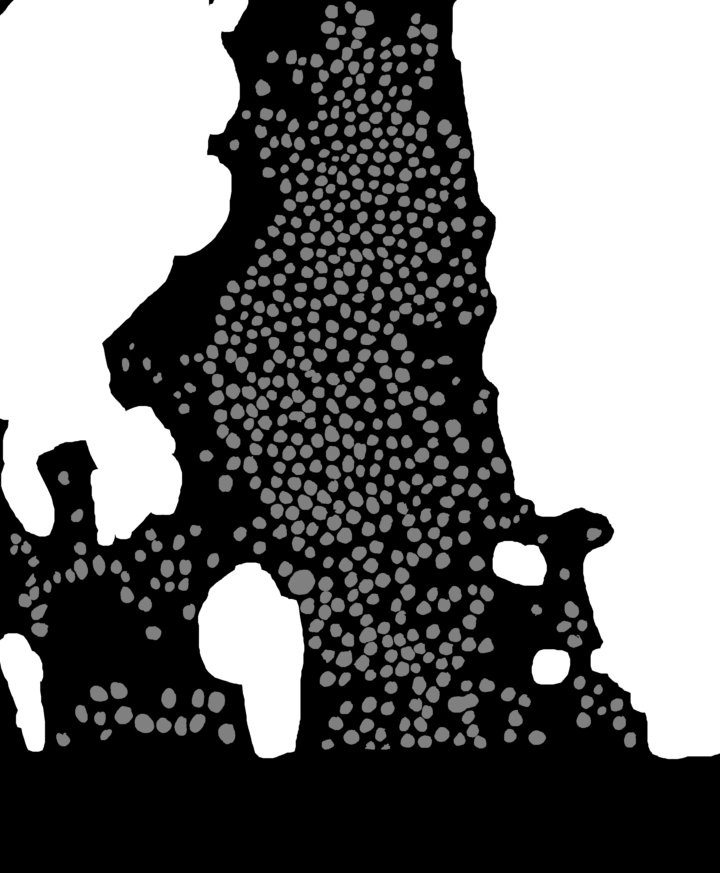

Supplement: S2 Dataset — (ZIP) [file pone.0312196.s003.zip › S3 Dataset/Training Images/S2_B1_21Feb24_010_16_background.ome.jpg]
